# Synthesis and Biological Evaluation of Novel C-28 Chloroacetamide-Modified Ursolic Acid Derivatives as Antibacterial Agents

**DOI:** 10.3390/microorganisms14081685

**Published:** 2026-07-31

**Authors:** Nan Cai, Tian Luan, Junchao Zhang, Ning Li, Xiu Zhang, Peng Gao, Jiaxuan Li, Hongyu Zhan, Fanhao Meng, Dajun Zhang

**Affiliations:** 1 Shenyang Key Laboratory for Phenomics, Liaoning Province Key Laboratory for Phenomics of Human Ethnic Specificity and Critical Illness, Shenyang Medical College, Shenyang 110034, China; ladycainan@126.com (N.C.);; 2 School of Pharmacy, China Medical University, Shenyang 110122, China; 3 Faculty of Dentistry, The University of Hong Kong, Hong Kong 999077, China

**Keywords:** ursolic acid, antibacterial activity, structure modification, SarA, biofilm inhibition

## Abstract

The escalating crisis of bacterial antimicrobial resistance necessitates the discovery of novel antibacterial agents with distinct mechanisms of action. Ursolic acid (UA), a naturally abundant pentacyclic triterpenoid, serves as a promising scaffold for structural modification. In this study, a series of 26 novel UA derivatives (**A1**–**A7** and **B1**–**B19**) were designed and synthesized by introducing various substituents at the C-28 carboxyl group via a chloroacetyl chloride linker. Their antibacterial activities were evaluated against *S. aureus*, *S. epidermidis*, *E. coli*, and *P. aeruginosa* using the microbroth dilution method. Among them, compound **B1** exhibited the most potent activity, with a minimum inhibitory concentration (MIC) of 37.5 μg/mL against *S. aureus* and 75 μg/mL against *E. coli*. Antibacterial kinetic and time-kill curve assays confirmed the sustained bactericidal effect of **B1**. Furthermore, **B1** significantly inhibited biofilm formation in both *S. aureus* and *E. coli* in a time-dependent manner. Molecular docking studies revealed that **B1** binds spontaneously to the *S. aureus* SarA protein through three hydrogen bonds and π-π stacking interactions, providing a structural basis for its antibacterial and anti-biofilm activities. This study demonstrates that piperazine-modified UA derivative **B1** is a promising antibacterial candidate and offers a new strategy for the structural optimization of ursolic acid.

## 1. Introduction

The relentless rise in bacterial resistance to conventional antibiotics has emerged as one of the most pressing global public health threats of the 21st century [1,2]. The overuse and misuse of antibiotics have accelerated this process, leading to the emergence of multidrug-resistant pathogens, which significantly compromise the efficacy of current therapeutic regimens [3,4,5]. A seminal report estimated that bacterial antimicrobial resistance was directly responsible for approximately 1.27 million deaths worldwide in 2019 [3]. Without the development of novel and effective antimicrobial strategies, this crisis is projected to escalate, potentially causing 10 million annual deaths by 2050 and becoming a leading cause of mortality globally [6]. This alarming trend underscores an urgent and unmet need for the discovery and development of new classes of antibacterial agents [7].

In the quest for new antibiotics, natural products have historically been, and continue to be, an invaluable source of inspiration and lead compounds [8]. Their unparalleled structural diversity and unique biological activities make them privileged scaffolds for drug discovery [9,10,11,12]. Among these, pentacyclic triterpenes have garnered significant attention due to their broad spectrum of pharmacological properties, including anti-inflammatory, antitumor, and antimicrobial activities [13]. Ursolic acid (UA), a ubiquitous pentacyclic triterpenoid found in various medicinal herbs and edible plants, exemplifies this potential [13]. UA and its derivatives have demonstrated promising antibacterial effects against a range of clinically relevant pathogens [14,15,16,17]. Its core structure, featuring a C-3 hydroxyl group, a C-12/C-13 olefinic bond, and a C-28 carboxylic acid group, offers multiple strategic sites for chemical modification to enhance its bioactivity and physicochemical properties [18]. Previous structure-activity relationship (SAR) studies have shown that modifications at the C-28 position can significantly influence the biological activity of UA derivatives [19,20]. Furthermore, the incorporation of nitrogen-containing heterocycles, such as piperazine and aniline moieties, has proven to be a successful strategy in medicinal chemistry, as these moieties themselves often possess excellent antibacterial activity [21,22,23,24,25].

**Figure 1:**
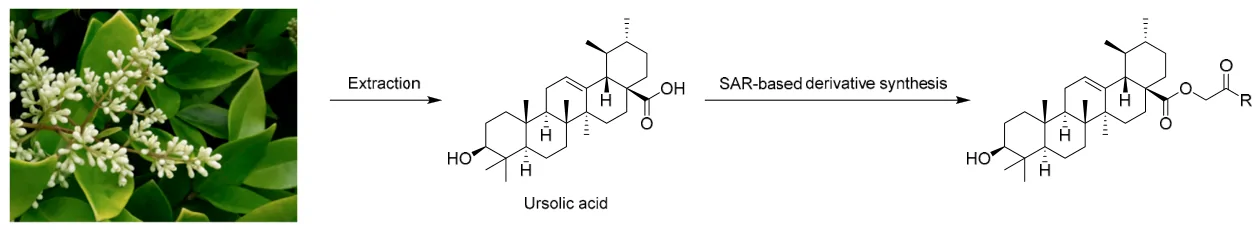
Design strategy for the target compounds.

Despite these advances, the antibacterial potential of UA derivatives incorporating a chloroacetamide linker at the C-28 position with various substituents remains a rich area for exploration. The rational design and synthesis of novel UA hybrids that combine the triterpene scaffold with potent antibacterial pharmacophores could yield compounds with improved efficacy. Therefore, this study aimed to design and synthesize a series of novel C-28 chloroacetamide-substituted ursolic acid derivatives by introducing three distinct classes of functional groups: 1,3,4-thiadiazole amides, piperazines, and aniline/aromatic heterocycles (Figure 1). All synthesized compounds were characterized by NMR and MS, and their in vitro antibacterial activities were evaluated against representative Gram-positive (*S. aureus* and *S. epidermidis*) and Gram-negative (*E. coli* and *P. aeruginosa*) bacteria. The most promising derivative, **B1**, was further investigated for its antibacterial kinetics, anti-biofilm activity, and potential molecular mechanism through molecular docking studies targeting the SarA protein. This work provides new insights into the structural modification of UA and identifies a potential lead compound for the development of novel antibacterial therapies.

## 2. Methods and Materials

### 2.1. Chemistry

#### 2.1.1. Synthesis of 1,3,4-Thiadiazole Intermediates **2a**–**2g**

The intermediates **2a**–**2g** were synthesized according to the method reported in reference [26]. At room temperature, 1.15 g (8.65 mmol) of 2-amino-5-mercapto-1,3,4-thiadiazole was added to a reaction flask, followed by 20 mL of ethanol as the solvent under magnetic stirring. An aqueous solution of potassium hydroxide (12.5 mol/L) was added dropwise via a dropping funnel over a period of 10 min. Subsequently, 1.50 g (9 mmol) of substituted benzyl chloride was slowly added dropwise to the mixture. After the addition, the reaction mixture was stirred for 2–3 h and monitored by thin-layer chromatography until completion. Upon completion, a large volume of water was added, and the mixture was stirred for 30 min. The precipitated solid was collected by suction filtration, washed, and dried to afford the intermediates **2a**–**2g**.

#### 2.1.2. Synthesis of 1,3,4-Thiadiazole Amide Intermediates **3a**–**3g**

The intermediates **3a**–**3g** were synthesized following the procedure described in reference [27]. The 1,3,4-thiadiazole intermediates **2a**–**2g** (4.14 mmol) were placed in a 250 mL three-necked flask, followed by the addition of 30 mL of dichloromethane as the solvent. The reaction mixture was cooled to −5 °C. After complete dissolution, triethylamine (6.22 mmol) was slowly added, and the mixture was stirred for 10 min. Subsequently, a solution of chloroacetyl chloride (4.97 mmol) in dichloromethane (10 mL) was added dropwise, and the reaction was allowed to proceed for 3–5 h. The reaction progress was monitored by TLC. Upon completion, the reaction mixture was washed thoroughly with saturated brine, and the organic layer was collected, dried over anhydrous sodium sulfate, and concentrated under reduced pressure. The resulting crude product was further purified by column chromatography to afford the intermediates **3a**–**3g**.

#### 2.1.3. Synthesis of the Target Compounds **A1**–**A7**

The target compounds **A1**–**A7** were synthesized according to the method reported in reference [28]. Specifically, ursolic acid (0.5 g, 1.10 mmol), anhydrous potassium carbonate (234.15 mg, 1.69 mmol), and acetonitrile (40 mL) were successively added to a 100 mL reaction flask. The mixture was stirred at 80 °C for 30 min. Then, the intermediates **3a**–**3g** (1.36 mmol) were added, and the reaction was continued for 8–12 h. The reaction progress was monitored by TLC. After completion, the solid was removed by suction filtration, and the solvent was evaporated under reduced pressure to obtain the crude product. The crude product was purified by column chromatography to afford the target compounds **A1**–**A7**.

2-((5-((4-(Trifluoromethyl)benzyl)thio)-1,3,4-thiadiazol-2-yl)amino)ethyl(1S,2R,4aS,6aS,6bR,8aR,10S,12aR,12bR,14bS)-10-hydroxy-1,2,6a,6b,9,9,12a-heptamethyl-1,3,4,5,6,6a,6b,7,8,8a,9,10,11,12,12a,12b,13,14b-octadecahydropicene-4a(2H)-carboxylate (**A1**)

White solid, yield: 35%; ^1^H NMR (400 MHz, DMSO-*d*_6_) *δ* 12.81 (s, 1H), 7.79–7.51 (m, 4H), 5.16 (s, 1H), 4.65 (d, *J* = 58.4 Hz, 4H), 4.27 (s, 1H), 3.00 (s, 1H), 2.25–2.11 (m, 1H), 1.94–1.70 (m, 4H), 1.55 (d, *J* = 75.2 Hz, 10H), 1.33–1.21 (m, 4H), 0.98 (d, *J* = 49.4 Hz, 10H), 0.75 (d, *J* = 66.6 Hz, 15H); ^13^C NMR (101 MHz, DMSO-*d*_6_) *δ* 175.88, 166.23, 158.51, 157.78, 141.92, 137.58, 129.68, 128.17, 127.85, 125.34, 125.30, 125.08, 122.77, 76.77, 61.40, 54.71(2C), 52.31, 47.40, 46.93, 41.54, 38.33, 38.25, 38.20, 36.69, 36.44, 36.00(2C), 32.57, 29.96, 28.19, 27.41, 26.95, 23.72, 23.18, 22.81, 20.92, 17.92, 16.87, 16.62, 16.01; HRMS (ESI) *m*/*z*: calculated C_42_H_57_F_3_N_3_O_4_S_2_ [M + H]^+^ 788.3738, found: 788.3731.

2-((5-((4-Methoxybenzyl)thio)-1,3,4-thiadiazol-2-yl)amino)ethyl(1S,2R,4aS,6aS,6bR,8aR,10S,12aR,12bR,14bS)-10-hydroxy-1,2,6a,6b,9,9,12a-heptamethyl-1,3,4,5,6,6a,6b,7,8,8a,9,10,11,12,12a,12b,13,14b-octadecahydropicene-4a(2H)-carboxylate (**A2**)

White solid, yield: 27%; ^1^HNMR (400 MHz, DMSO-*d*_6_) *δ* 12.77 (s, 1H), 7.35–7.28 (m, 2H), 6.93–6.85 (m, 2H), 5.16 (t, *J* = 3.7 Hz, 1H), 4.80–4.66 (m, 2H), 4.43 (s, 2H), 4.27 (d, *J* = 5.0 Hz, 1H), 3.73 (s, 3H), 3.00 (dt, *J* = 10.5, 5.3 Hz, 1H), 2.17 (d, *J* = 11.2 Hz, 1H), 2.09–1.72 (m, 4H), 1.70–1.42 (m, 10H), 1.30 (dd, *J* = 17.1, 9.9 Hz, 4H), 1.06–0.65 (m, 25H); ^13^C NMR (101 MHz, DMSO-*d*_6_) *δ* 175.85, 166.15, 158.68, 158.48, 158.23, 137.58, 130.17(2C), 128.21, 125.08, 113.91(2C), 76.77, 61.41, 55.02(2C), 54.72, 52.32, 47.40, 46.94, 41.54, 38.33, 38.26, 38.20, 37.22, 37.15, 36.44, 36.01, 32.58, 29.98, 28.20, 27.42, 26.95, 23.74, 23.19, 22.82, 20.93, 17.93, 16.88, 16.63, 16.02, 15.16; HRMS (ESI) *m*/*z*: calculated C_42_H_60_N_3_O_5_S_2_ [M + H]^+^ 750.3970, found: 750.3962.

2-((5-((4-(Trifluoromethoxy)benzyl)thio)-1,3,4-thiadiazol-2-yl)amino)ethyl (1S,2R,4aS,6aS,6bR,8aR,10S,12aR,12bR,14bS)-10-hydroxy-1,2,6a,6b,9,9,12a-heptamethyl-1,3,4,5,6,6a,6b,7,8,8a,9,10,11,12,12a,12b,13,14b-octadecahydropicene-4a(2H)-carboxylate (**A3**)

White solid, yield: 37%; ^1^H NMR (400 MHz, DMSO-*d*_6_) *δ* 12.80 (s, 1H), 7.53 (d, *J* = 8.2 Hz, 2H), 7.32 (d, *J* = 8.2 Hz, 2H), 5.17 (d, *J* = 3.4 Hz, 1H), 4.80–4.66 (m, 2H), 4.52 (s, 2H), 4.27 (d, *J* = 5.0 Hz, 1H), 3.00 (dt, *J* = 10.4, 5.4 Hz, 1H), 2.16 (d, *J* = 11.2 Hz, 1H), 2.09–1.75 (m, 4H), 1.68–1.42 (m, 10H), 1.35–1.25 (m, 4H), 1.05–0.64 (m, 25H); ^13^C NMR (101 MHz, DMSO-*d*_6_*) δ* 175.87, 166.21, 158.48, 157.92, 147.58, 137.58, 136.42, 130.83(2C), 125.08, 121.27, 121.00, 118.71, 76.77, 61.40, 54.72(2C), 52.32, 47.40, 46.94, 41.55, 38.33, 38.26, 38.20, 36.50(2C), 36.44, 36.00, 32.58, 29.97, 28.19, 27.41, 26.94, 23.73, 23.18, 22.81, 20.92, 17.92, 16.87, 16.62, 16.00, 15.15; HRMS (ESI) *m*/*z*: calculated C_42_H_57_F_3_N_3_O_5_S_2_ [M + H]^+^ 804.3686 found: 804.3684.

2-((5-((4-Bromobenzyl)thio)-1,3,4-thiadiazol-2-yl)amino)ethyl (1S,2R,4aS,6aS,6bR,8aR,10S,12aR,12bR,14bS)-10-hydroxy-1,2,6a,6b,9,9,12a-heptamethyl-1,3,4,5,6,6a,6b,7,8,8a,9,10,11,12,12a,12b,13,14b-octadecahydropicene-4a(2H)-carboxylate (**A4**)

White solid, yield: 42%; ^1^H NMR (400 MHz, DMSO-*d*_6_) *δ* 12.78 (s, 1H), 7.43 (dd, *J* = 63.5, 8.0 Hz, 4H), 5.16 (s, 1H), 4.74 (t, *J* = 10.3 Hz, 2H), 4.46 (s, 2H), 4.33–4.21 (m, 1H), 3.00 (s, 1H), 2.01 (d, *J* = 14.8 Hz, 1H), 1.78 (d, *J* = 36.5 Hz, 4H), 1.68–1.40 (m, 10H), 1.34–1.19 (m, 4H), 1.16–0.56 (m, 25H); ^13^C NMR (101 MHz, DMSO-*d*_6_) *δ* 175.86, 166.20, 158.41, 157.94, 137.56, 136.36, 131.35(2C), 131.07(2C), 125.07, 120.6, 76.77, 61.40, 54.71(2C), 52.31, 47.39, 46.93, 41.54, 38.32, 38.23, 38.21, 36.71(2C), 36.44, 36.00, 32.58, 29.96, 28.20, 27.41, 26.95, 23.73, 23.19, 22.82, 20.93, 17.93, 16.87, 16.62, 16.02, 15.16; HRMS (ESI) *m*/*z*: calculated C_42_H_57_BrN_3_O_4_S_2_ [M + H]^+^ 800.2949, found: 800.2951.

2-((5-((4-Fluorobenzyl)thio)-1,3,4-thiadiazol-2-yl)amino)ethyl(1S,2R,4aS,6aS,6bR,8aR,10S,12aR,12bR,14bS)-10-hydroxy-1,2,6a,6b,9,9,12a-heptamethyl-1,3,4,5,6,6a,6b,7,8,8a,9,10,11,12,12a,12b,13,14b-octadecahydropicene-4a(2H)-carboxylate (**A5**)

White solid, yield: 40%; ^1^H NMR (400 MHz, DMSO-*d*_6_) *δ* 12.78 (s, 1H), 7.48–7.40 (m, 2H), 7.15 (t, *J* = 8.8 Hz, 2H), 5.16 (t, *J* = 3.6 Hz, 1H), 4.80–4.66 (m, 2H), 4.48 (s, 2H), 4.27 (d, *J* = 5.1 Hz, 1H), 3.00 (dt, *J* = 10.4, 5.4 Hz, 1H), 2.03 (dt, *J* = 13.9, 7.1 Hz, 1H), 1.94–1.67 (m, 4H), 1.64–1.34 (m, 10H), 1.28 (dd, *J* = 26.6, 13.9 Hz, 4H), 1.11–0.66 (m, 25H); ^13^C NMR (101 MHz, DMSO-*d*_6_) *δ* 175.87, 166.21, 162.68, 160.25, 158.39, 137.57, 132.98, 130.99, 130.91, 125.08, 115.38, 115.17, 76.77, 61.41, 54.72(2C), 52.31, 47.40, 46.94, 41.54, 38.33, 38.26, 38.20, 36.68(2C), 6.44, 36.00, 32.58, 29.97, 28.20, 27.41, 26.94, 23.73, 23.19, 22.82, 20.93, 17.92, 16.87, 16.63, 16.01, 15.16; HRMS (ESI) *m*/*z*: calculated C_41_H_57_FN_3_O_4_S_2_ [M + H]^+^ 738.3770, found: 738.3772.

2-((5-((3-Bromobenzyl)thio)-1,3,4-thiadiazol-2-yl)amino)ethyl(1S,2R,4aS,6aS,6bR,8aR,10S,12aR,12bR,14bS)-10-hydroxy-1,2,6a,6b,9,9,12a-heptamethyl-1,3,4,5,6,6a,6b,7,8,8a,9,10,11,12,12a,12b,13,14b-octadecahydropicene-4a(2H)-carboxylate (**A6**)

White solid, yield: 37%; ^1^H NMR (400 MHz, DMSO-*d*_6_) *δ* 12.80 (s, 1H), 7.62 (s, 1H), 7.43 (dd, *J* = 26.2, 7.8 Hz, 2H), 7.28 (t, *J* = 7.8 Hz, 1H), 5.16 (s, 1H), 4.74 (t, *J* = 10.4 Hz, 2H), 4.49 (s, 2H), 4.27 (d, *J* = 5.0 Hz, 1H), 3.00 (dt, *J* = 10.2, 5.5 Hz, 1H), 2.02 (d, *J* = 12.6 Hz, 1H), 1.80 (dd, *J* = 25.6, 12.6 Hz, 4H), 1.65–1.37 (m, 10H), 1.33–1.19 (m, 4H), 1.08–0.65 (m, 25H; ^13^C NMR (101 MHz, DMSO-*d*_6_) *δ* 175.86, 166.21, 158.46, 157.87, 139.70, 137.57, 131.62, 130.56, 130.33, 127.95, 125.07, 121.5, 76.77, 61.40, 54.71(2C), 52.31, 47.40, 46.93, 41.54, 38.32, 38.23, 38.21, 36.61(2C), 36.44, 36.00, 32.58, 29.97, 28.20, 27.41, 26.94, 23.73, 23.19, 22.82, 20.93, 17.93, 16.87, 16.63, 16.02, 15.16; HRMS (ESI) *m*/*z*: calculated C_41_H_57_BrN_3_O_4_S_2_ [M + H]^+^ 800.2949, found: 800.2955.

2-((5-((3-(Trifluoromethyl)benzyl)thio)-1,3,4-thiadiazol-2-yl)amino)ethyl(1S,2R,4aS,6aS,6bR,8aR,10S,12aR,12bR,14bS)-10-hydroxy-1,2,6a,6b,9,9,12a-heptamethyl-1,3,4,5,6,6a,6b,7,8,8a,9,10,11,12,12a,12b,13,14b-octadecahydropicene-4a(2H)-carboxylate (**A7**)

White solid, yield: 42%; ^1^H NMR (400 MHz, DMSO-*d*_6_) *δ* 12.80 (s, 1H), 7.84–7.51 (m, 4H), 5.16 (d, *J* = 3.6 Hz, 1H), 4.82–4.67 (m, 2H), 4.59 (s, 2H), 4.27 (d, *J* = 5.0 Hz, 1H), 3.00 (dt, *J* = 10.1, 5.3 Hz, 1H), 2.02 (dt, *J* = 13.6, 6.8 Hz, 1H), 1.91–1.65 (m, 4H), 1.64–1.34 (m, 10H), 1.33–1.19 (m, 4H), 1.07–0.64 (m, 25H); ^13^CNMR (101 MHz, DMSO-*d*_6_) *δ* 175.87, 166.20, 158.52, 157.71, 138.56, 137.71, 133.02, 129.52, 129.27, 128.95, 125.59, 125.37, 124.17, 76.77, 61.40, 54.71(2C), 52.31, 47.40, 46.93, 41.54, 38.32, 38.25, 38.20, 36.67(2C), 36.44, 35.99, 32.57, 29.9, 28.19, 27.41, 26.94, 23.72, 23.18, 22.81, 20.92, 17.92, 16.87, 16.61, 16.00, 15.14; HRMS (ESI) *m*/*z*: calculated C_42_H_57_F_3_N_3_O_4_S_2_ [M + H]^+^ 788.3738, found: 788.3737.

#### 2.1.4. Synthesis of Substituted Piperazine Polyheterocyclic Intermediates (**5a**–**5l**)

The intermediates **5a**–**5l** were synthesized according to the method reported in reference [27]. Substituted piperazines (**4a**–**4l**) (1.00 g, 5.20 mmol) were weighed, and triethylamine (7.80 mmol, 1.5 equiv) and chloroacetyl chloride (6.24 mmol, 1.2 equiv) were added successively. Following the synthetic procedure for intermediates **3a**–**3g**, intermediates **5a**–**5l** were obtained.

#### 2.1.5. Synthesis of the Target Compounds **B1**–**B12**

Intermediates **5a**–**5l** were treated with ursolic acid (0.50 g, 1.10 mmol) following the synthetic procedure described for target compounds **A1**–**A7** [28], affording the piperazine-containing ursolic acid derivatives **B1**–**B12**.

2-(4-(4-Methoxyphenyl)piperazin-1-yl)-2-oxoethyl(1S,2R,4aS,6aS,6bR,8aR,10S,12aR,12bR,14bS)-10-hydroxy-1,2,6a,6b,9,9,12a-heptamethyl-1,3,4,5,6,6a,6b,7,8,8a,9,10,11,12,12a,12b,13,14b-octadecahydropicene-4a(2H)-carboxylate (**B1**)

White solid, yield: 62%; ^1^H NMR (400 MHz, DMSO-*d*_6_*) δ* 6.91 (d, *J* = 9.1 Hz, 2H), 6.83 (d, *J* = 9.1 Hz, 2H), 5.15 (s, 1H), 4.77 (d, *J* = 14.5 Hz, 1H), 4.68 (d, *J* = 14.5 Hz, 1H), 4.28 (d, *J* = 5.1 Hz, 1H), 3.68 (s, 3H), 3.51 (dt, *J* = 10.3, 5.2 Hz, 4H), 2.99 (dd, *J* = 13.9, 8.8 Hz, 5H), 2.17 (d, *J* = 11.2 Hz, 1H), 2.07–1.75 (m, 4H), 1.74–1.43 (m, 10H), 1.35–1.19 (m, 4H), 1.04–0.64 (m, 25H); ^13^CNMR (101 MHz, DMSO-*d*_6_) *δ* 175.59, 164.78, 153.33, 145.07, 137.81, 124.87(2C), 118.04(2C), 114.25, 76.78, 60.71, 55.13(2C), 54.74, 52.37, 50.13, 49.69, 47.40, 46.98, 43.87, 41.56, 41.21, 38.44, 38.34, 38.22, 36.46(2C), 36.15, 32.63, 30.09, 28.22, 27.43, 26.97, 23.81, 23.22, 22.86, 21.00, 17.95, 16.96, 16.80, 16.06, 15.20; HRMS (ESI) *m*/*z*: calculated C_43_H_65_N_2_O_5_ [M + H]^+^ 689.4889, found: 689.4825.

2-Oxo-2-(4-(pyrimidin-2-yl)piperazin-1-yl)ethyl(1S,2R,4aS,6aS,6bR,8aR,10S,12aR,12bR,14bS)-10-hydroxy-1,2,6a,6b,9,9,12a-heptamethyl-1,3,4,5,6,6a,6b,7,8,8a,9,10,11,12,12a,12b,13,14b-octadecahydropicene-4a(2H)-carboxylate (**B2**)

White solid, yield: 27%; ^1^H NMR (400 MHz, DMSO-*d*_6_) *δ* 8.38 (d, *J* = 4.8 Hz, 2H), 6.66 (t, *J* = 4.7 Hz, 1H), 5.15 (t, *J* = 3.7 Hz, 1H), 4.78 (d, *J* = 14.6 Hz, 1H), 4.70 (d, *J* = 14.5 Hz, 1H), 4.29 (d, *J* = 5.1 Hz, 1H), 3.75 (q, *J* = 6.6 Hz, 3H), 3.68 (s, 1H), 3.32 (s, 4H), 3.00 (dt, J = 10.6, 5.4 Hz, 1H), 2.17 (d, *J* = 11.2 Hz, 1H), 2.08–1.74 (m, 4H), 1.71–1.42 (m, 10H), 1.36–1.23 (m, 4H), 1.06–0.67 (m, 25H); ^13^C NMR (101 MHz, CDCl_3_) *δ* 176.98, 165.63, 161.41, 157.86(2C), 138.11, 125.72, 110.65, 79.06, 61.03, 55.27, 52.83, 48.37, 47.62, 44.56, 43.68, 43.49, 42.13, 41.76, 39.61, 39.17, 38.82(2C), 38.69, 37.04, 36.67, 33.06, 30.75, 28.21, 28.09, 27.30, 24.31, 23.59, 23.37, 21.27, 18.39, 17.18, 17.09, 15.71, 15.54; HRMS (ESI) *m*/*z*: calculated C_43_H_61_N_4_O_4_ [M + H]^+^ 661.4688, found: 661.4627.

2-(4-(2-Methoxyphenyl)piperazin-1-yl)-2-oxoethyl(1S,2R,4aS,6aS,6bR,8aR,10S,12aR,12bR,14bS)-10-hydroxy-1,2,6a,6b,9,9,12a-heptamethyl-1,3,4,5,6,6a,6b,7,8,8a,9,10,11,12,12a,12b,13,14b-octadecahydropicene-4a(2H)-carboxylate (**B3**)

White solid, yield: 53%; ^1^H NMR (400 MHz, DMSO-*d*_6_) *δ* 7.01–6.92 (m, 2H), 6.91–6.85 (m, 2H), 5.15 (t, *J* = 3.6 Hz, 1H), 4.77 (d, *J* = 14.5 Hz, 1H), 4.68 (d, *J* = 14.5 Hz, 1H), 4.29 (d, *J* = 5.1 Hz, 1H), 3.79 (s, 3H), 3.63–3.43 (m, 4H), 3.06–2.90 (m, 4H), 2.89 (s, 1H), 2.17 (d, *J* = 11.2 Hz, 1H), 2.06–1.74 (m, 4H), 1.73–1.39 (m, 10H), 1.37–1.22 (m, 4H), 1.13–0.62 (m, 25H); ^13^C NMR (101 MHz, CDCl_3_) *δ* 176.99, 165.35, 152.32, 140.50, 138.16, 125.70, 123.76, 121.15, 118.53, 111.42, 79.06, 61.03, 55.52, 55.27, 52.82, 50.89, 50.56, 48.36, 47.62, 45.03, 42.13(2C), 39.62 39.17, 38.82(2C), 38.68, 37.05, 36.67, 33.07, 30.76, 28.21, 28.11, 27.29, 24.31, 23.60, 23.38, 21.28, 18.39, 17.17, 17.10, 15.71, 15.54; HRMS (ESI) *m*/*z*: calculated C_43_H_65_N_3_O_5_ [M + H]^+^ 689.4889, found: 689.4818.

2-(4-(4-Chlorophenyl)piperazin-1-yl)-2-oxoethyl(1S,2R,4aS,6aS,6bR,8aR,10S,12aR,12bR,14bS)-10-hydroxy-1,2,6a,6b,9,9,12a-heptamethyl-1,3,4,5,6,6a,6b,7,8,8a,9,10,11,12,12a,12b,13,14b-octadecahydropicene-4a(2H)-carboxylate (**B4**)

White solid, yield: 42%;^1^ H NMR (400 MHz, DMSO-*d*_6_) *δ* 7.25 (d, *J* = 8.6 Hz, 2H), 6.96 (d, *J* = 8.5 Hz, 2H), 5.14 (s, 1H), 4.69 (d, *J* = 14.3 Hz, 2H), 4.29 (d, *J* = 5.0 Hz, 1H), 3.32 (s, 4H), 3.12 (d, *J* = 18.7 Hz, 4H), 2.99 (s, 1H), 2.16 (d, *J* = 11.2 Hz, 1H), 1.91 (dd, *J* = 72.6, 13.3 Hz, 4H), 1.56 (d, *J* = 85.8 Hz, 10H), 1.25 (s, 4H), 1.06–0.68 (m, 25H); ^13^C NMR (101 MHz, CDCl_3_) *δ* 177.02, 165.43, 149.35, 138.13, 129.25(2C), 125.99, 125.71, 118.14(2C), 79.06, 60.87, 55.27, 52.84, 49.80, 49.51, 48.38, 47.61, 44.56, 42.14, 41.69, 39.62, 39.17, 38.83(2C), 38.68, 37.05, 36.68, 33.07, 30.75, 28.21, 28.10, 27.29, 24.32, 23.59, 23.38, 21.27, 18.39, 17.19, 17.10, 15.71, 15.55; HRMS (ESI) *m*/*z*: calculated C_42_H_62_ClN_2_O_4_ [M + H]^+^ 693.4393, found: 693.4343.

2-(4-(4-Fluorophenyl)piperazin-1-yl)-2-oxoethyl(1S,2R,4aS,6aS,6bR,8aR,10S,12aR,12bR,14bS)-10-hydroxy-1,2,6a,6b,9,9,12a-heptamethyl-1,3,4,5,6,6a,6b,7,8,8a,9,10,11,12,12a,12b,13,14b-octadecahydropicene-4a(2H)-carboxylate (**B5**)

White solid, yield: 54%; ^1^H NMR (400 MHz, DMSO-*d*_6_) *δ* 7.06 (t, *J* = 8.9 Hz, 2H), 6.97 (dd, *J* = 9.2, 4.7 Hz, 2H), 5.14 (d, *J* = 3.8 Hz, 1H), 4.82–4.65 (m, 2H), 4.29 (d, *J* = 5.0 Hz, 1H), 3.52 (dt, *J* = 10.8, 5.4 Hz, 4H), 3.15–2.95 (m, 5H), 2.17 (d, *J* = 11.3 Hz, 1H), 2.06–2.00 (m, 1H), 1.92–1.72 (m, 3H), 1.71–1.40 (m, 10H), 1.40–1.21 (m, 4H), 1.120.59 (m, 25H); ^13^C NMR (101 MHz, CDCl_3_) *δ* 177.03, 165.43, 138.13, 125.73, 125.70(2C), 119.05, 116.03, 115.81(2C), 70.03, 60.85, 55.26, 52.83, 51.00, 50.71, 48.36, 47.60, 44.61, 42.13, 41.77, 39.62, 39.16, 38.82(2C), 38.68, 37.04, 36.67, 33.06, 30.74, 28.21, 28.09, 27.28, 24.31, 23.58, 23.37, 21.26, 18.38, 17.18, 17.09, 15.71, 15.54; HRMS (ESI) *m*/*z*: calculated C_42_H_62_FN_2_O_4_ [M + H]^+^ 677.4689, found: 677.4625.

2-(4-(2,3-Dichlorophenyl)piperazin-1-yl)-2-oxoethyl(1S,2R,4aS,6aS,6bR,8aR,10S,12aR,12bR,14bS)-10-hydroxy-1,2,6a,6b,9,9,12a-heptamethyl-1,3,4,5,6,6a,6b,7,8,8a,9,10,11,12,12a,12b,13,14b-octadecahydropicene-4a(2H)-carboxylate (**B6**)

White solid, yield: 33%; ^1^H NMR (400 MHz, DMSO-*d*6) *δ* 7.33 (d, *J* = 4.8 Hz, 2H), 7.22–7.02 (m, 1H), 5.15 (s, 1H), 4.88–4.60 (m, 2H), 4.28 (d, *J* = 5.1 Hz, 1H), 3.55 (d, *J* = 15.5 Hz, 4H), 2.96 (d, *J* = 14.6 Hz, 5H), 2.17 (d, *J* = 11.2 Hz, 1H), 2.03–1.82 (m, 4H), 1.74–1.46 (m, 10H), 1.24 (s, 4H), 0.95–0.68 (m, 25H); ^13^C NMR (101 MHz, CDCl_3_) *δ* 177.02, 165.52, 150.33, 138.15, 134.36, 127.84, 127.70, 125.71, 125.55, 118.96, 79.07, 60.94, 55.27, 52.84, 51.55, 51.11, 48.37, 47.62, 44.98, 42.14, 42.09, 39.63, 39.18, 38.83, 38.68(2C), 37.05, 36.67, 33.07, 30.76, 28.21, 28.11, 27.29, 24.32, 23.60, 23.38, 21.28, 18.39, 17.19, 17.10, 15.71, 15.55; HRMS (ESI) *m*/*z*: calculated C_42_H_61_Cl_2_N_2_O_4_ [M + H]^+^ 727.4004, found: 727.3959.

2-oxo-2-(4-Phenylpiperazin-1-yl)ethyl(1S,2R,4aS,6aS,6bR,8aR,10S,12aR,12bR,14bS)-10-hydroxy-1,2,6a,6b,9,9,12a-heptamethyl-1,3,4,5,6,6a,6b,7,8,8a,9,10,11,12,12a,12b,13,14b-octadecahydropicene-4a(2H)-carboxylate (**B7**)

White solid, yield: 47%; ^1^HNMR (400 MHz, DMSO-*d*_6_) *δ* 7.23 (dd, *J* = 8.5, 7.0 Hz, 2H), 6.95 (d, *J* = 8.2 Hz, 2H), 6.81 (t, *J* = 7.3 Hz, 1H), 5.15 (t, *J* = 3.6 Hz, 1H), 4.83–4.64 (m, 2H), 4.29 (d, *J* = 5.1 Hz, 1H), 3.52 (d, *J* = 6.3 Hz, 4H), 3.09 (s, 4H), 3.00 (dt, *J* = 10.4, 5.4 Hz, 1H), 2.17 (d, *J* = 11.2 Hz, 1H), 2.07–1.74 (m, 4H), 1.72–1.40 (m, 10H), 1.38–1.22 (m, 4H), 1.22–0.59 (m, 25H); ^13^C NMR (101 MHz, CDCl_3_) *δ* 177.02, 165.43, 138.14, 129.41(5C), 125.72, 117.00, 79.07, 60.93, 55.28, 52.84, 49.90,49.59, 48.38, 47.62, 44.65, 42.15, 41.76, 39.63, 39.18, 38.84(2C), 38.69, 37.06, 36.68, 33.08, 30.76, 28.22, 28.11, 27.30, 24.33, 23.60, 23.39, 21.28, 18.40, 17.20, 17.10, 15.72, 15.55; HRMS (ESI) *m*/*z*: calculated C_42_H_63_N_2_O_4_ [M + H]^+^ 659.4783, found: 659.4724.

2-(4-(3-Methoxyphenyl)piperazin-1-yl)-2-oxoethyl(1S,2R,4aS,6aS,6bR,8aR,10S,12aR,12bR,14bS)-10-hydroxy-1,2,6a,6b,9,9,12a-heptamethyl-1,3,4,5,6,6a,6b,7,8,8a,9,10,11,12,12a,12b,13,14b-octadecahydropicene-4a(2H)-carboxylate (**B8**)

White solid, yield: 56%; ^1^H NMR (400 MHz, DMSO-*d*_6_) *δ* 7.12 (t, *J* = 8.2 Hz, 1H), 6.53 (dd, *J* = 8.2, 2.3 Hz, 1H), 6.46 (t, *J* = 2.3 Hz, 1H), 6.39 (dd, *J* = 8.2, 2.3 Hz, 1H), 5.14 (d, *J* = 3.9 Hz, 1H), 4.77 (d, *J* = 14.5 Hz, 1H), 4.69 (d, *J* = 14.5 Hz, 1H), 4.28 (d, *J* = 5.0 Hz, 1H), 3.71 (s, 3H), 3.56–3.47 (m, 4H), 3.11 (d, *J* = 17.5 Hz, 4H), 3.01–2.94 (m, 1H), 2.16 (d, *J* = 11.2 Hz, 1H), 1.96–1.70 (m, 4H), 1.69–1.42 (m, 10H), 1.28 (q, *J* = 13.5, 12.8 Hz, 4H), 1.03–0.65 (m, 25H); ^13^C NMR (101 MHz, DMSO-*d*_6_) *δ* 175.0, 164.81, 160.17, 152.06, 137.80, 129.64, 124.8, 108.40, 104.64, 101.98, 76.78, 60.71, 54.85(2C), 54.75, 52.37, 48.48, 48.14, 47.40, 46.98, 43.63, 41.55, 41.01, 38.43, 38.34, 38.22, 36.46, 36.15(2C), 32.63, 30.09, 28.21, 27.44, 26.96, 23.81, 23.22, 22.85, 21.00, 17.95, 16.95, 16.78, 16.05, 15.19; HRMS (ESI) *m*/*z*: calculated C_43_H_65_N_2_O_5_ [M + H]^+^ 689.4889, found: 689.4824.

2-oxo-2-(4-Phenylpiperazin-1-yl)ethyl(1S,2R,4aS,6aS,6bR,8aR,10S,12aR,12bR,14bS)-10-hydroxy-1,2,6a,6b,9,9,12a-heptamethyl-1,3,4,5,6,6a,6b,7,8,8a,9,10,11,12,12a,12b,13,14b-octadecahydropicene-4a(2H)-carboxylate (**B9**)

White solid, yield: 50%; ^1^H NMR (400 MHz, DMSO-*d*_6_) *δ* 8.16–8.09 (m, 1H), 7.55 (ddd, *J* = 8.9, 7.1, 2.0 Hz, 1H), 6.85 (d, *J* = 8.6 Hz, 1H), 6.67 (dd, *J* = 7.1, 4.9 Hz, 1H), 5.15 (t, *J* = 3.6 Hz, 1H), 4.79 (d, *J* = 14.6 Hz, 1H), 4.70 (d, *J* = 14.5 Hz, 1H), 4.29 (d, *J* = 5.1 Hz, 1H), 3.51 (q, *J* = 8.8, 7.7 Hz, 8H), 3.00 (dt, *J* = 10.6, 5.4 Hz, 1H), 2.17 (d, *J* = 11.3 Hz, 1H), 2.06–1.75 (m, 4H), 1.72–1.41 (m, 10H), 1.37–1.21 (m, 4H), 1.07–0.67 (m, 25H); ^13^C NMR (101 MHz, CDCl_3_) *δ* 176.95, 165.57, 158.88, 147.96, 138.10, 137.85, 125.70, 114.09, 107.33, 79.03, 60.96, 55.26(2C), 52.81, 48.35, 47.60, 45.20, 45.05, 44.44, 42.11, 41.48, 39.60, 39.15, 38.80, 38.67, 37.03, 36.65, 33.05, 30.73, 28.20, 28.08, 27.28, 24.29, 23.58, 23.36, 21.26, 18.37, 17.16, 17.07, 15.70, 15.53; HRMS (ESI) *m*/*z*: calculated C_41_H_62_N_3_O_4_ [M + H]^+^ 660.4736, found: 660.4675.

2-(4-(4-Nitrophenyl)piperazin-1-yl)-2-oxoethyl(1S,2R,4aS,6aS,6bR,8aR,10S,12aR,12bR,14bS)-10-hydroxy-1,2,6a,6b,9,9,12a-heptamethyl-1,3,4,5,6,6a,6b,7,8,8a,9,10,11,12,12a,12b,13,14b-octadecahydropicene-4a(2H)-carboxylate (**B10**)

Yellow solid yield: 13%; ^1^H NMR (400 MHz, DMSO-*d*_6_) *δ* 8.12–8.03 (m, 2H), 7.07–6.98 (m, 2H), 5.14 (d, *J* = 3.9 Hz, 1H), 4.79 (d, *J* = 14.6 Hz, 1H), 4.71 (d, *J* = 14.6 Hz, 1H), 4.29 (d, *J* = 5.1 Hz, 1H), 3.64–3.43 (m, 8H), 3.00 (dt, *J* = 10.4, 5.3 Hz, 1H), 2.16 (d, *J* = 11.3 Hz, 1H), 2.07–1.99 (m, 1H), 1.95–1.76 (m, 3H), 1.71–1.42 (m, 10H), 1.37–1.22 (m, 4H), 1.05–0.65 (m, 25H); ^13^C NMR (101 MHz, CDCl_3_) *δ* 177.07, 165.71, 154.27, 139.43, 138.10, 126.03(2C), 125.73, 113.28(2C), 79.04, 60.72, 55.24, 52.84, 48.39, 47.59, 47.04(2C), 44.00, 42.14, 41.18, 39.62, 39.16, 38.81(2C), 38.67, 37.04, 36.68, 33.06, 30.73, 28.20, 28.09, 27.27, 24.33, 23.58, 23.37, 21.26, 18.38, 17.18, 17.09, 15.72, 15.54; HRMS (ESI) *m*/*z*: calculated C_42_H_62_N_3_O_6_ [M + H]^+^ 704.4634, found: 704.4623.

2-(4-(4-Cyanophenyl)piperazin-1-yl)-2-oxoethyl(1S,2R,4aS,6aS,6bR,8aR,10S,12aR,12bR,14bS)-10-hydroxy-1,2,6a,6b,9,9,12a-heptamethyl-1,3,4,5,6,6a,6b,7,8,8a,9,10,11,12,12a,12b,13,14b-octadecahydropicene-4a(2H)-carboxylate (**B11**)

White solid, yield: 16%; ^1^H NMR (400 MHz, DMSO-*d*_6_) *δ* 7.60 (d, *J* = 8.7 Hz, 2H), 7.02 (d, *J* = 8.8 Hz, 2H), 5.14 (t, *J* = 3.6 Hz, 1H), 4.78 (d, *J* = 14.6 Hz, 1H), 4.70 (d, *J* = 14.6 Hz, 1H), 4.29 (d, *J* = 5.1 Hz, 1H), 3.61–3.48 (m, 4H), 3.45–3.32 (m, 4H), 3.00 (dt, *J* = 10.5, 5.4 Hz, 1H), 2.16 (d, *J* = 11.2 Hz, 1H), 2.08–2.00 (m, 1H), 1.80 (tt, *J* = 16.0, 8.1 Hz, 3H), 1.70–1.40 (m, 10H), 1.38–1.22 (m, 4H), 1.07–0.67 (m, 25H); ^13^C NMR (101 MHz, CDCl_3_) *δ* 177.05, 165.60, 152.82, 138.10, 133.70(2C), 125.71, 119.68, 114.83(2C), 101.70, 79.03, 60.74, 55.24, 52.83, 48.37, 47.58, 47.39,47.25, 44.01, 42.13, 41.28, 39.61, 39.15, 38.82(2C), 38.67, 37.03, 36.67, 33.05, 30.73, 28.20, 28.08, 27.27, 24.32, 23.58, 23.37, 21.25, 18.38, 17.17, 17.08, 15.71, 15.54; HRMS (ESI) *m*/*z*: calculated C_43_H_62_N_3_O_4_ [M + H]^+^ 684.4736, found: 684.4712.

2-Oxo-2-(4-(*p*-tolyl)piperazin-1-yl)ethyl(1S,2R,4aS,6aS,6bR,8aR,10S,12aR,12bR,14bS)-10-hydroxy-1,2,6a,6b,9,9,12a-heptamethyl-1,3,4,5,6,6a,6b,7,8,8a,9,10,11,12,12a,12b,13,14b-octadecahydropicene-4a(2H)-carboxylate (**B12**)

White solid, yield: 54%; ^1^H NMR (400 MHz, DMSO-*d*_6_) *δ* 7.04 (d, *J* = 8.2 Hz, 2H), 6.86 (d, *J* = 8.6 Hz, 2H), 5.15 (t, *J* = 3.5 Hz, 1H), 4.78 (d, *J* = 14.6 Hz, 1H), 4.69 (d, *J* = 14.5 Hz, 1H), 4.29 (d, *J* = 5.0 Hz, 1H), 3.52 (dt, J = 10.4, 5.0 Hz, 4H), 3.03 (dd, *J* = 16.7, 11.5 Hz, 5H), 2.21 (s, 3H), 2.01 (dt, *J* = 13.6, 7.0 Hz, 1H), 1.89–1.68 (m, 4H), 1.67–1.40 (m, 10H), 1.36–1.20 (m, 4H), 1.07–0.66 (m, 25H); ^13^C NMR (101 MHz, CDCl_3_) *δ* 176.99, 165.38, 138.14, 130.57, 129.89(4C), 125.71, 117.28, 79.07, 60.96, 55.28, 52.83, 50.37, 50.03, 48.37, 47.62,44.72, 42.14, 41.87, 39.62, 39.17, 38.83(2C), 38.69, 37.05, 36.67, 33.07, 30.75, 28.21, 28.10, 27.30, 24.31, 23.60, 23.38, 21.27, 20.55, 18.39, 17.18, 17.10, 15.71, 15.54; HRMS (ESI) *m*/*z*: calculated C_43_H_65_N_2_O_4_ [M + H]^+^ 673.4938, found: 673.4871.

#### 2.1.6. Synthesis of Substituted Aniline/Aromatic Heterocyclic Intermediates (**5m**–**5s**)

The intermediates **5m**–**5s** were synthesized according to the method reported in reference [27]. Substituted aniline/aromatic heterocyclic starting materials (**4m**–**4s**) (1.00 g, 7.45 mmol) were weighed, and triethylamine (10.76 mmol, 1.5 equiv) and chloroacetyl chloride (9.48 mmol, 1.2 equiv) were added successively. Following the synthetic procedure for intermediates **3a**–**3g**, intermediates **5m**–**5s** were obtained.

#### 2.1.7. Synthesis of the Target Compounds **B13**–**B19**

Intermediates **5m**–**5s** were treated with ursolic acid (0.50 g, 1.10 mmol) following the synthetic procedure described for target compounds **A1**–**A7** [28], affording the substituted aniline/aromatic heterocyclic ursolic acid derivatives (target compounds **B13**–**B19**).

2-((2-Chlorophenyl)amino)-2-oxoethyl(1S,2R,4aS,6aS,6bR,8aR,10S,12aR,12bR,14bS)-10-hydroxy-1,2,6a,6b,9,9,12a-heptamethyl-1,3,4,5,6,6a,6b,7,8,8a,9,10,11,12,12a,12b,13,14b-octadecahydropicene-4a(2H)-carboxylate (**B13**)

White solid, yield: 60%; ^1^H NMR (400 MHz, DMSO-*d*_6_) *δ* 9.47 (s, 1H), 7.79 (d, *J* = 9.7 Hz, 1H), 7.51 (d, *J* = 9.5 Hz, 1H), 7.34 (t, *J* = 8.2 Hz, 1H), 7.20 (t, *J* = 8.5 Hz, 1H), 5.19 (d, *J* = 3.7 Hz, 1H), 4.74–4.56 (m, 2H), 4.29 (d, *J* = 5.1 Hz, 1H), 3.00 (dt, *J* = 10.4, 5.4 Hz, 1H), 2.21 (d, *J* = 11.2 Hz, 1H), 2.09–1.72 (m, 4H), 1.70–1.42 (m, 10H), 1.29 (q, *J* = 11.4, 10.5 Hz, 4H), 1.06–0.62 (m, 25H); ^13^C NMR (101 MHz, CDCl_3_) *δ* 175.69, 165.56, 138.07, 133.81, 129.20, 128.02, 126.20, 125.36, 123.04, 121.80, 79.06, 63.37, 55.27, 53.15, 48.59, 47.54, 42.14, 39.59, 39.15, 39.04, 38.81, 38.65, 37.01, 36.88, 32.95, 30.59, 28.20, 28.10, 27.28, 24.48, 23.74, 23.35, 21.20, 18.33, 17.12, 17.05, 15.67, 15.44; HRMS (ESI) *m*/*z*: calculated C_38_H_53_ClNO_4_ [M − H]^−^ 622.3667, found: 622.3630.

2-((2,5-Difluorophenyl)amino)-2-oxoethyl(1S,2R,4aS,6aS,6bR,8aR,10S,12aR,12bR,14bS)-10-hydroxy-1,2,6a,6b,9,9,12a-heptamethyl-1,3,4,5,6,6a,6b,7,8,8a,9,10,11,12,12a,12b,13,14b-octadecahydropicene-4a(2H)-carboxylate (**B14**)

White solid, yield: 58%; ^1^H NMR (400 MHz, DMSO-*d*_6_) *δ* 10.03 (s, 1H), 7.87 (ddd, *J* = 10.0, 6.2, 3.2 Hz, 1H), 7.32 (td, *J* = 9.8, 5.1 Hz, 1H), 6.97 (tt, *J* = 7.6, 3.3 Hz, 1H), 5.16 (d, *J* = 3.7 Hz, 1H), 4.71 (d, *J* = 15.0 Hz, 1H), 4.64 (d, *J* = 14.9 Hz, 1H), 4.29 (d, *J* = 5.1 Hz, 1H), 3.00 (dt, *J* = 10.5, 5.5 Hz, 1H), 2.18 (d, *J* = 11.3 Hz, 1H), 2.08–1.72 (m, 4H), 1.70–1.42 (m, 10H), 1.29 (td, *J* = 17.7, 14.8, 6.8 Hz, 4H), 1.07–0.67 (m, 25H); ^13^C NMR (101 MHz, CDCl_3_) *δ* 175.78, 165.67, 138.22, 126.32, 126.06, 115.49, 115.37, 110.88, 109.22, 108.92, 79.05, 63.13, 55.26, 53.28, 48.59, 47.52, 42.17, 39.57, 39.17, 38.97, 38.81, 38.64, 36.99, 36.86, 32.93, 30.58, 28.19, 27.98, 27.26, 24.56, 23.71, 23.32, 21.18, 18.33, 17.08, 17.01, 15.67, 15.40; HRMS (ESI) *m*/*z*: calculated C_38_H_52_F_2_NO_4_ [M − H]^−^ 624.3868, found: 624.3832.

2-((4-Methoxyphenyl)amino)-2-oxoethyl(1S,2R,4aS,6aS,6bR,8aR,10S,12aR, 12bR,14bS)-10-hydroxy-1,2,6a,6b,9,9,12a-heptamethyl-1,3,4,5,6,6a,6b,7,8,8a,9,10,11,12,12a,12b,13,14b-octadecahydropicene-4a(2H)-carboxylate (**B15**)

White solid, yield: 47%; ^1^H NMR (400 MHz, DMSO-*d*_6_) *δ* 9.84 (s, 1H), 7.49–7.40 (m, 2H), 6.99–6.76 (m, 2H), 5.17 (t, *J* = 3.6 Hz, 1H), 4.60–4.46 (m, 2H), 4.28 (d, *J* = 5.1 Hz, 1H), 3.71 (s, 3H), 2.99 (dt, *J* = 10.4, 5.4 Hz, 1H), 2.19 (d, *J* = 11.3 Hz, 1H), 2.09–2.00 (m, 1H), 1.90–1.72 (m, 3H), 1.67–1.39 (m, 10H), 1.37–1.20 (m, 4H), 1.05–0.58 (m, 25H); ^13^C NMR (101 MHz, CDCl_3_) *δ* 175.95, 165.58, 157.16, 139.58, 129.58, 125.53, 122.81(2C), 114.40(2C), 79.00, 63.09, 55.59, 55.22, 53.15, 48.54, 47.51, 42.31, 39.55, 39.23, 38.95, 38.79, 38.56, 36.96, 36.88, 32.88, 30.58, 28.18, 27.89, 27.22, 24.60, 23.74, 23.15, 21.19, 18.28, 17.14, 17.04, 15.65, 15.27; HRMS (ESI) *m*/*z*: calculated C_39_H_56_NO_5_ [M − H]^−^ 618.4162, found: 618.4145.

2-((3-Chlorophenyl)amino)-2-oxoethyl(1S,2R,4aS,6aS,6bR,8aR,10S,12aR,12bR,14bS)-10-hydroxy-1,2,6a,6b,9,9,12a-heptamethyl-1,3,4,5,6,6a,6b,7,8,8a,9,10,11,12,12a,12b,13,14b-octadecahydropicene-4a(2H)-carboxylate (**B16**)

White solid, yield: 54%; ^1^H NMR(400 MHz, DMSO-*d*_6_) *δ* 10.22 (s, 1H), 7.76 (t, *J* = 2.0 Hz, 1H), 7.35 (dt, *J* = 15.9, 8.3 Hz, 2H), 7.11 (dt, *J* = 7.6, 1.7 Hz, 1H), 5.16 (t, *J* = 3.7 Hz, 1H), 4.64–4.50 (m, 2H), 4.28 (d, *J* = 5.1 Hz, 1H), 2.99 (dt, *J* = 10.3, 5.5 Hz, 1H), 2.18 (d, *J* = 11.3 Hz, 1H), 2.07–1.73 (m, 4H), 1.68–1.42 (m, 10H), 1.35–1.21 (m, 4H), 1.04–0.65 (m, 25H); ^13^C NMR (101 MHz, CDCl_3_) *δ* 176.03, 165.69, 139.40, 137.85, 134.90, 130.23, 125.60, 125.28, 120.76, 118.54, 79.04, 63.17, 55.23, 53.17, 48.61, 47.51, 42.32, 39.56, 39.24, 38.95, 38.80, 38.59, 36.98, 36.93, 32.89, 30.58, 28.19, 27.94, 27.24, 24.63, 23.72, 23.18, 21.19, 18.29, 17.16, 17.06, 15.65, 15.30; HRMS (ESI) *m*/*z*: calculated C_38_H_53_ClNO_4_ [M − H]^−^ 622.3667, found: 622.3610.

2-((4-Cyanophenyl)amino)-2-oxoethyl(1S,2R,4aS,6aS,6bR,8aR,10S,12aR,12bR,14bS)-10-hydroxy-1,2,6a,6b,9,9,12a-heptamethyl-1,3,4,5,6,6a,6b,7,8,8a,9,10,11,12,12a,12b,13,14b-octadecahydropicene-4a(2H)-carboxylate (**B17**)

White solid, yield: 48%; ^1^HNMR (400 MHz, DMSO-*d*_6_) *δ* 10.49 (s, 1H), 7.77 (d, *J* = 8.9 Hz, 2H), 7.72 (d, *J* = 8.9 Hz, 2H), 5.16 (t, *J* = 3.6 Hz, 1H), 4.67–4.54 (m, 2H), 4.28 (d, *J* = 5.1 Hz, 1H), 2.99 (dt, *J* = 10.2, 5.4 Hz, 1H), 2.18 (d, *J* = 11.3 Hz, 1H), 2.08–2.00 (m, 1H), 1.89–1.71 (m, 3H), 1.69–1.41 (m, 10H), 1.27 (dd, *J* = 24.6, 11.5 Hz, 4H), 1.05–0.65 (m, 25H); ^13^CNMR (101 MHz, DMSO-*d*_6)_ *δ* 175.95, 166.26, 142.69, 137.71, 133.33(2C), 125.01, 119.08, 118.93(2C), 105.15, 76.77, 62.18, 54.72, 52.31, 47.34, 46.94, 41.55, 38.38, 38.34, 38.28, 38.19, 36.45, 36.02, 32.56, 30.01, 28.21, 27.43, 26.96, 26.32, 23.74, 23.25, 22.82, 20.97, 17.94, 16.92, 16.59, 16.05, 15.15; HRMS (ESI) *m*/*z*: calculated C_39_H_53_N_2_O_4_ [M − H]^−^ 613.4009, found: 613.3991.

2-((4-Methylpyridin-2-yl)amino)-2-oxoethyl(1S,2R,4aS,6aS,6bR,8aR,10S,12aR, 12bR,14bS)-10-hydroxy-1,2,6a,6b,9,9,12a-heptamethyl-1,3,4,5,6,6a,6b,7,8,8a,9,10,11, 12,12a,12b,13,14b-octadecahydropicene-4a(2H)-carboxylate (**B18**)

White solid, yield: 56%; ^1^H NMR (400 MHz, DMSO-*d*_6_) *δ* 10.42 (s, 1H), 8.16 (d, *J* = 5.0 Hz, 1H), 7.85 (s, 1H), 6.95 (dd, *J* = 5.0, 1.5 Hz, 1H), 5.18 (t, *J* = 3.6 Hz, 1H), 4.71–4.57 (m, 2H), 4.29 (d, *J* = 5.1 Hz, 1H), 3.00 (dt, *J* = 10.5, 5.5 Hz, 1H), 2.30 (s, 3H), 2.19 (d, *J* = 11.2 Hz, 1H), 2.12–1.74 (m, 4H), 1.73–1.42 (m, 10H), 1.35–1.22 (m, 4H), 1.07–0.67 (m, 25H); ^13^C NMR (101 MHz, CDCl_3_) *δ* 176.22, 166.10, 150.52, 150.18, 147.52, 138.46, 126.42, 121.65, 114.96, 79.13, 63.08, 55.30, 52.94, 48.62, 47.67, 42.22, 39.64, 39.32, 38.94, 38.87, 38.68, 37.07, 36.90, 32.93, 30.71, 28.26, 28.07, 27.33, 24.66, 23.80, 23.22, 21.53, 21.29, 18.40, 17.16, 17.06, 15.73, 15.42; HRMS (ESI) *m*/*z*: calculated C_38_H_57_N_2_O_4_ [M + H]^+^ 605.4314, found: 605.4260.

2-Oxo-2-(thiazol-2-ylamino)ethyl(1S,2R,4aS,6aS,6bR,8aR,10S,12aR,12bR,14bS)-10-hydroxy-1,2,6a,6b,9,9,12a-heptamethyl-1,3,4,5,6,6a,6b,7,8,8a,9,10,11,12,12a,12b,13,14b-octadecahydropicene-4a(2H)-carboxylate (**B19**)

White solid, yield: 58%; ^1^H NMR (400 MHz, DMSO-*d*_6_) *δ* 12.23 (s, 1H), 7.47 (d, *J* = 3.6 Hz, 1H), 7.24 (d, *J* = 3.5 Hz, 1H), 5.17 (d, *J* = 3.8 Hz, 1H), 4.77–4.63 (m, 2H), 4.29 (d, *J* = 5.1 Hz, 1H), 3.00 (dt, *J* = 10.5, 5.4 Hz, 1H), 2.18 (d, *J* = 11.2 Hz, 1H), 2.10–1.76 (m, 4H), 1.72–1.42 (m, 10H), 1.36–1.20 (m, 4H), 1.07–0.67 (m, 25H); ^13^C NMR (101 MHz, CDCl_3_), *δ* 176.22, 165.34, 157.35, 138.98, 137.22, 126.16, 114.26, 79.08, 62.38, 55.23, 52.86, 48.59, 47.61, 42.23, 39.56, 39.26, 38.87, 38.81, 38.59, 37.04, 36.78, 32.85, 30.59, 28.20, 27.97, 27.27, 24.54, 23.73, 23.22, 21.21, 18.33, 17.09, 17.06, 15.67, 15.36; HRMS (ESI) *m*/*z*: calculated C_35_H_52_N_2_O_4_S [M + H]^+^ 597.3721, found: 597.3667.

### 2.2. Bioassay

All bacterial strains used in this study, including *Staphylococcus aureus* ATCC29213, *Staphylococcus epidermidis* ATCC12228, *Escherichia coli* ATCC25922, and *Pseudomonas aeruginosa* CGMCC10104, were purchased from the American Type Culture Collection (ATCC) and maintained in our laboratory.

#### 2.2.1. Measurement of Minimum Inhibitory Concentrations (MICs)

The MICs were determined according to a previously reported method with minor modifications [29]. Test compounds were dissolved in dimethyl sulfoxide (DMSO), and the final DMSO concentration was maintained below 1% (*v*/*v*). Tetracycline and ursolic acid were used as positive controls, while 1% DMSO served as a blank control. Stock solutions were sterilized by filtration through a 0.22 μm membrane filter. In a sterile 96-well plate, culture medium and test compound solutions were sequentially added to each well to a total volume of 100 μL. Subsequently, 100 μL of bacterial suspension (1.5 × 10^6^ CFU/mL) was added to each well and mixed thoroughly. The final concentrations of the test compounds in the wells ranged from 0.59 to 150 μg/mL (150, 75, 37.5, 18.75, 9.38, 4.69, 2.34, 1.17, and 0.59 μg/mL). The plates were incubated at 37 °C for 24 h, after which the optical density at 600 nm was measured using a microplate reader. All experiments were performed in triplicate.

#### 2.2.2. Assessment of Time-Dependent Bactericidal Activity

Time-kill assays were performed according to a previously described protocol with minor modifications [30]. Single colonies of *S. aureus* and *E. coli* were cultured in Luria–Bertani (LB) broth at 37 °C with shaking (200 rpm) for 16–18 h, then adjusted to 1.5 × 10^6^ CFU/mL. Compound **B1** and tetracycline were dissolved in DMSO and diluted with distilled water to 150 μg/mL. Negative controls received 1% DMSO alone. In sterile 5 mL tubes, 1 mL of bacterial suspension was mixed with 1 mL of either LB broth (negative control), **B1**, or tetracycline (positive control). Tubes were incubated at 37 °C. At 0, 0.5, 1, 2, 4, 8, and 24 h, 100 μL aliquots were removed, serially diluted (10^−1^–10^−7^) in LB broth, and plated on agar. After 24 h at 37 °C, colonies were counted (30–300 CFU/plate). All experiments were performed in triplicate.

#### 2.2.3. Crystal Violet-Based Quantification of Biofilm Formation Inhibition

The inhibitory effect of compound **B1** on biofilm formation was evaluated using a crystal violet staining method based on previously reported protocols with minor modifications [29]. Overnight cultures of *S. aureus* and *E. coli* were adjusted to an appropriate density. In a 96-well plate, 100 μL of bacterial suspension was mixed with 100 μL of compound **B1** at a final concentration of 150 μg/mL. Sterile LB broth containing 1% DMSO served as a blank control. Each condition was set up in triplicate. The plate was incubated at 37 °C for 4, 20, and 24 h. After each incubation period, planktonic cells were gently removed by aspiration. The wells were washed three times with sterile distilled water to eliminate non-adherent bacteria. The attached biofilm was then fixed with 200 μL of 99% methanol for 15 min, followed by air-drying. Subsequently, the biofilm was stained with 1% (*w*/*v*) crystal violet solution for 15 min. Excess dye was removed by washing three times with distilled water, and the plate was air-dried. The crystal violet bound to the biofilm was solubilized with 30% (*v*/*v*) acetic acid, and the absorbance was measured at 570 nm using a microplate reader. All experiments were performed in triplicate (*n* = 3), and the data was expressed as mean ± standard error of the mean (SEM).

#### 2.2.4. Molecular Docking

Molecular docking was performed using AutoDock Vina (version 1.5.6). The three-dimensional structure of compound **B1** was first subjected to energy minimization to achieve a stable low-energy conformation using Discovery Studio (DS, version 2019). The minimized ligand structure was saved in PDB format. The target protein was prepared by importing its structure into PyMol (version 3.1), where non-essential ligands and water molecules were removed. The resulting protein structure was also saved in PDB format. Hydrogen atoms and partial charges were then added to the protein using AutoDockTools, and the prepared protein was designated as the receptor and saved in PDBQT format. Similarly, the ligand (compound **B1**) was processed by adding hydrogen atoms and charges, assigned as the ligand, and its torsional bonds and rotatable bonds were checked before being saved in PDBQT format. A grid box covering the entire protein was defined to allow comprehensive ligand binding. Following grid parameter configuration, 50 independent docking runs were carried out. After docking, the conformation with the highest occurrence frequency and the most favorable binding affinity was selected as the representative result. Visualization and analysis of the docking poses, including three-dimensional structures and surface models, were conducted using PyMol 3.1 and Discovery Studio 2019 [31].

#### 2.2.5. Molecular Dynamics Simulation Analysis

A 100 ns molecular dynamics (MD) simulation was conducted on the protein–ligand complex using GROMACS 2025 [32]. The AMBER99SB-ILDN force field was employed to describe the protein, whereas the ligand topology was generated using the GAFF2 force field. The resulting topologies for the protein and ligand were merged after ensuring consistency of atom types. Periodic boundary conditions were applied, and the complex was placed in a cubic box with a minimum distance of 1.2 nm between the solute and the box edges. The system was solvated with the TIP3P water model and neutralized by adding Na^+^ or Cl^−^ ions to reach a physiological concentration of 0.15 M.

Energy minimization was performed first, followed by a two-step equilibration procedure. The equilibration consisted of 1,000,000 steps each for NVT (constant number of particles, volume, and temperature) and NPT (constant number of particles, pressure, and temperature) ensembles, with each step corresponding to a 2 ns simulation and a coupling constant of 0.1 ps. Subsequently, the production MD run was carried out for 50,000,000 steps with a 2 fs time step, yielding a total simulation time of 100 ns under constant temperature (310 K) and pressure (1 bar). Trajectory data were saved every 1000 steps for subsequent analyses.

The binding free energy after system stabilization was calculated using the MM-PBSA method. The following properties were computed with GROMACS utilities: root-mean-square deviation (RMSD) for overall structural stability; root-mean-square fluctuation (RMSF) for residue flexibility; radius of gyration (Rg) for compactness; solvent-accessible surface area (SASA) for solvent exposure; the number of protein–ligand hydrogen bonds for interaction stability; two-dimensional and three-dimensional free energy landscapes; and MM-PBSA results, including binding free energy and per-residue energy decomposition.

### 2.3. Statistical Analysis

All experimental data are presented as the mean ± standard error of the mean (SEM). One-way ANOVA was conducted using GraphPad Prism 10.0 to evaluate statistical significance, with a threshold of *p* < 0.05 indicating a significant difference.

## 3. Results and Discussion

### 3.1. Chemistry

**Scheme 1:**
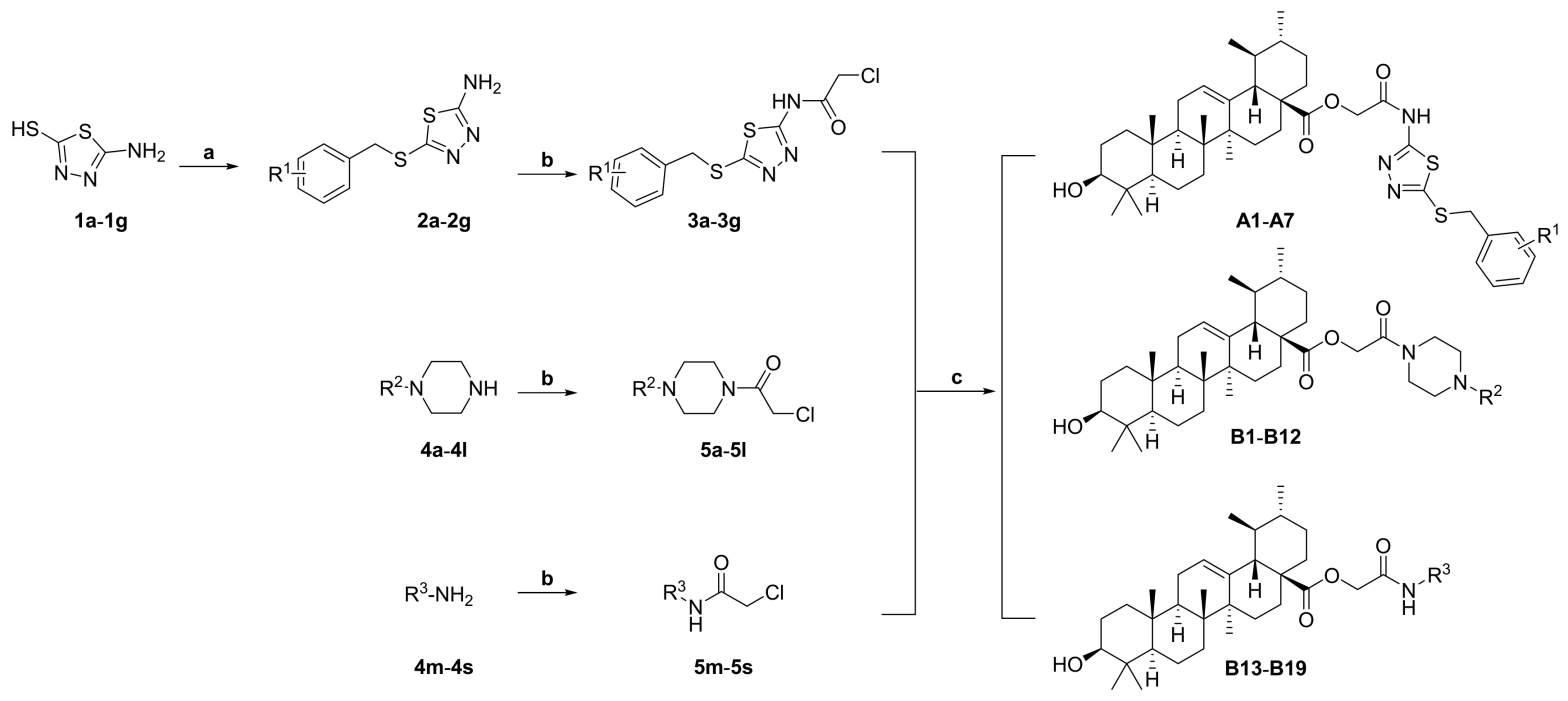
Synthetic route of target compounds **A1**–**A7** and **B1**–**B19**. Reagents and conditions: (a) substituted benzyl chloride, KOH, EtOH, r.t., 3 h; (b) chloroacetyl chloride, Et_3_N, CH_2_Cl_2_, 5 h, −5 °C; (c) ursolic acid, K_2_CO_3_, CH_3_CN, 80 °C, 12 h.

In this study, a series of 26 novel UA derivatives (**A1**–**A7** and **B1**–**B19**) were designed and synthesized by introducing three distinct classes of functional groups—1,3,4-thiadiazole amides, piperazines, and aniline/aromatic heterocycles—at the C-28 position of UA using chloroacetyl chloride as a linker (Scheme 1). The synthetic routes are illustrated in Figure 1. Three key classes of intermediates were first prepared. The 1,3,4-thiadiazole intermediates (**2a**–**2g**) were synthesized via coupling of 2-amino-5-mercapto-1,3,4-thiadiazole with various substituted benzyl chlorides, which were subsequently reacted with chloroacetyl chloride to afford the corresponding 1,3,4-thiadiazole amide intermediates (**3a**–**3g**). Coupling of intermediates **3a**–**3g** with the C-28 carboxyl group of UA yielded the target compounds **A1**–**A7**. Similarly, piperazine intermediates (**5a**–**5l**) were synthesized by amidation of various substituted piperazines with chloroacetyl chloride in the presence of triethylamine, followed by conjugation with UA to generate target compounds **B1**–**B12**. The substituted aniline/aromatic heterocycle intermediates (**5m**–**5s**) were prepared by reaction of the corresponding aniline or heterocyclic starting materials with chloroacetyl chloride, and subsequent coupling with UA afforded target compounds **B13**–**B19**. All target compounds were obtained in satisfactory yields and purified by column chromatography. The chemical structures of all synthesized derivatives were unequivocally confirmed by ^1^H NMR, ^13^C NMR, and HRMS, with the spectral data provided in the supporting data.

### 3.2. Bioassay

#### 3.2.1. Minimum Inhibitory Concentration (MIC) Testing

The MIC values of all synthesized compounds were determined against four bacterial strains. Compounds not listed in Table 1 exhibited MIC values >150 μg/mL against all four strains. As shown in Table 1, the 1,3,4-thiadiazole derivatives (**A1**–**A7**) did not exhibit significant antibacterial activity (MIC > 150 μg/mL). However, several piperazine derivatives (**B1**–**B12**) and aniline/aromatic heterocyclic derivatives (**B13**–**B19**) showed inhibitory effects. Among all derivatives, compound **B1** showed the most potent activity, with an MIC of 37.5 μg/mL against *S. aureus* and 75 μg/mL against *E. coli*. This suggests that the introduction of a 4-methoxyphenyl piperazine moiety at the C-28 position is beneficial for antibacterial activity, particularly against Gram-positive bacteria. The enhanced activity may be attributed to the basic nitrogen of the piperazine ring, which can form hydrogen bonds with bacterial target proteins, and the hydrophobic 4-methoxyphenyl group, which may improve membrane affinity [33]. Compound **B14**, bearing a 2,5-difluoroaniline group, also showed moderate activity against *S. aureus* (MIC 75 μg/mL), indicating that halogenated aniline moieties can also contribute to antibacterial efficacy.

#### 3.2.2. Antibacterial Kinetics and Time-Kill Assay

**Figure 2:**
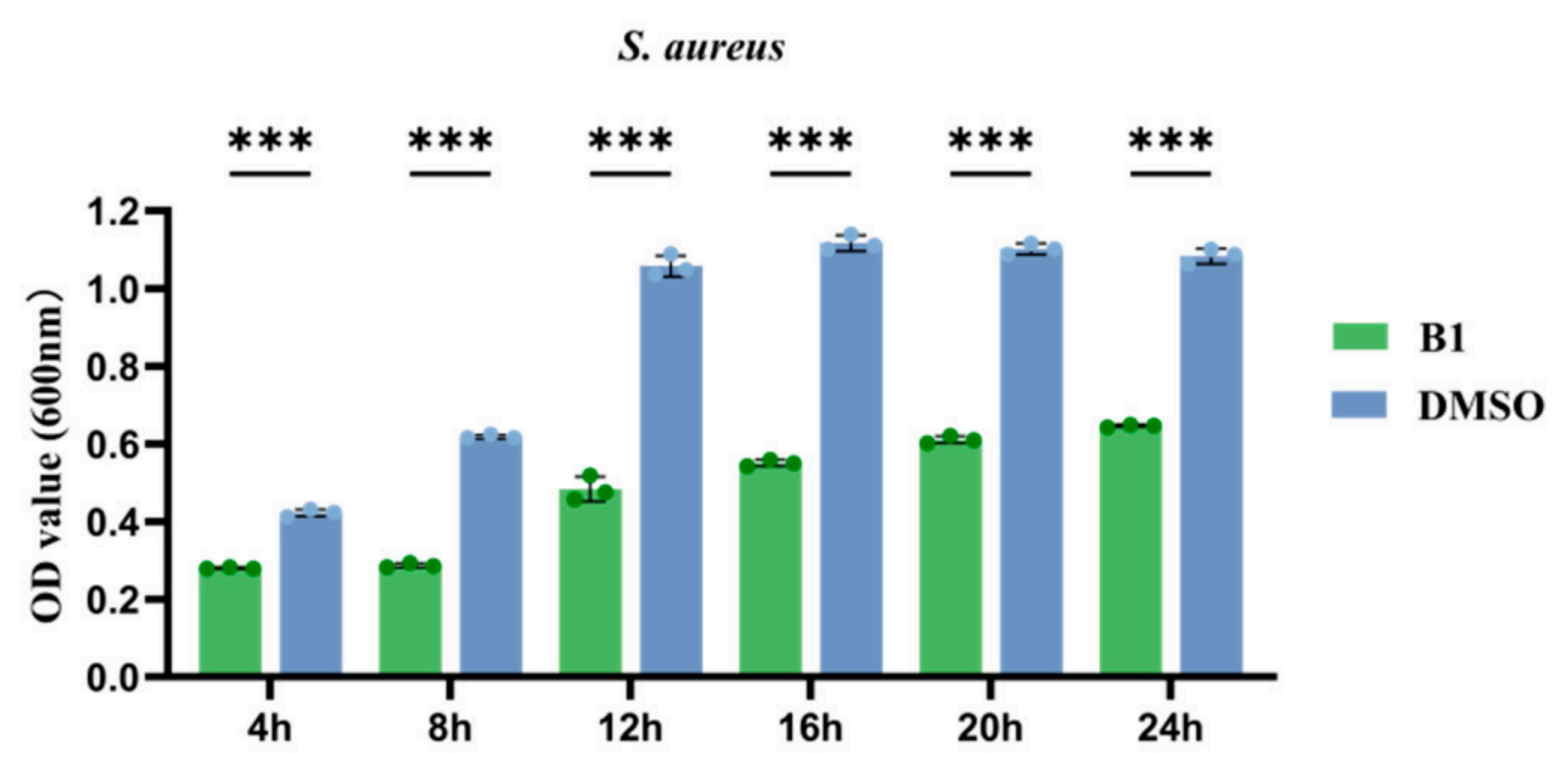
Time-dependent antibacterial activity of **B1** against *S. aureus*. *** *p* < 0.001.

**Figure 3:**
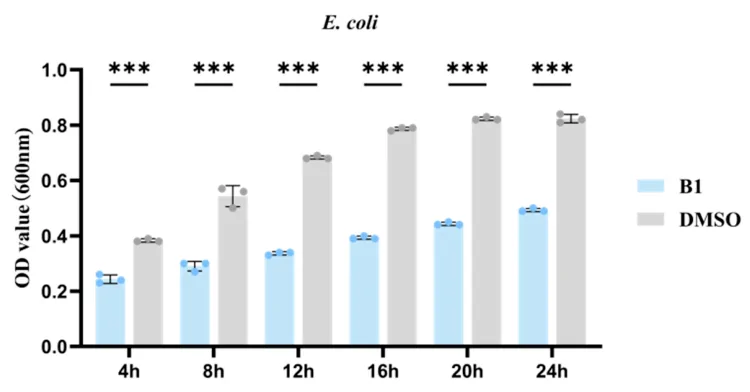
Time-dependent antibacterial activity of **B1** against *E. coli*. *** *p* < 0.001.

Given that compound **B1** exhibited the most potent antibacterial activity in the MIC assays (Table 1), its antibacterial kinetics were further investigated against *S. aureus* and *E. coli*. All experiments were carried out in triplicate (*n* = 3), and statistical significance was determined by one-way analysis of variance (ANOVA). As shown in Figure 2 and Figure 3, treatment with **B1** significantly suppressed bacterial growth over the entire 24 h observation period (*p* < 0.001). The maximum growth inhibition rates were 58.52% at 12 h for *S. aureus* and 52.46% at 16 h for *E. coli*, indicating a sustained bacteriostatic effect.

**Figure 4:**
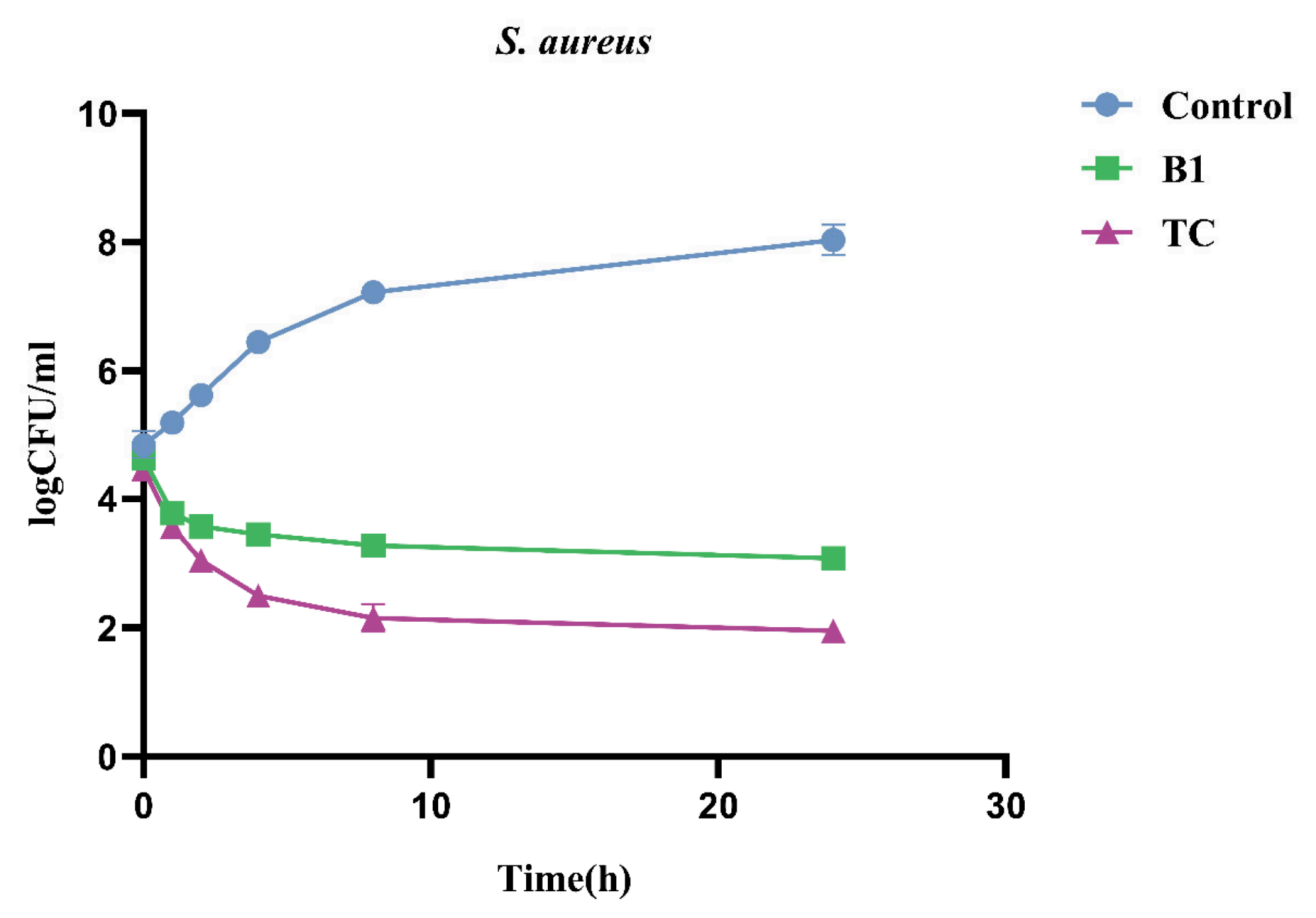
Time-kill curves of compound **B1** and tetracycline against *S. aureus*.

**Figure 5:**
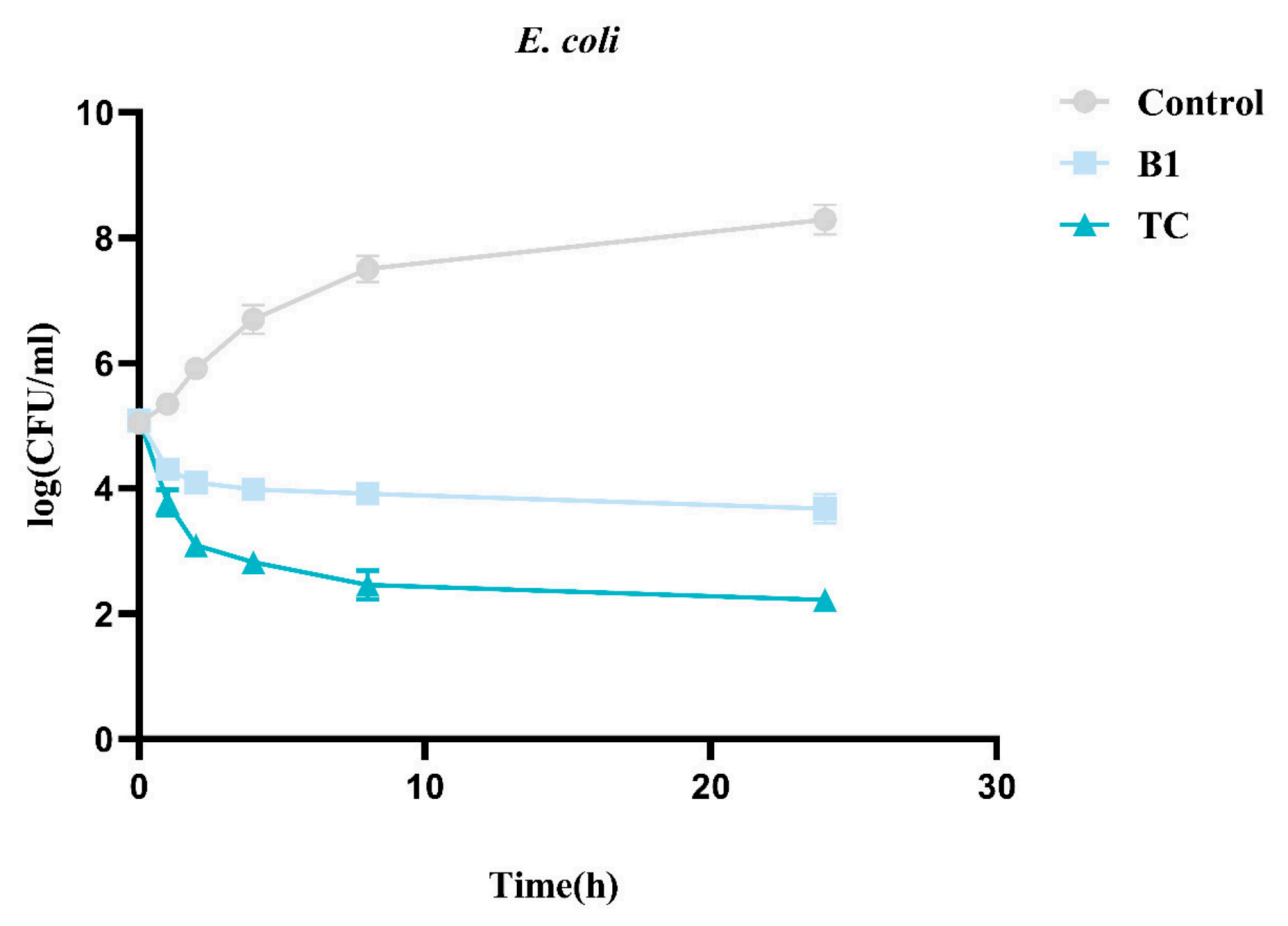
Time-kill curves of compound **B1** and tetracycline against *E. coli*.

To evaluate whether **B1** exerts a bactericidal rather than merely bacteriostatic action, a time-kill curve assay was performed at a higher concentration. As depicted in Figure 4 and Figure 5, **B1** produced a rapid and sustained reduction in viable counts. Within the first hour, the initial bacterial load decreased by 0.88 log_10_ CFU/mL for *S. aureus* and by 0.70 log_10_ CFU/mL for *E. coli*. After 24 h of exposure, the reductions reached 1.72 log_10_ CFU/mL and 1.33 log_10_ CFU/mL, respectively, with no evidence of regrowth. The time-dependent antibacterial and time-kill profiles of compound **B1** provide important insights into its pharmacodynamic characteristics. When compared to the positive control tetracycline, **B1** showed a slower killing rate within the first few hours (0.88 log_10_ CFU/mL reduction for *S. aureus* at 1 h vs. a faster reduction typically seen with tetracycline), yet the overall reduction after 24 h was comparable in magnitude. This delayed but sustained bactericidal effect may be advantageous in reducing the selective pressure associated with rapid-onset antibiotics, potentially lowering the risk of resistance development. Overall, the kinetic and time-kill data demonstrate that **B1** is a slow-acting but sustained bactericidal agent against both *S. aureus* and *E. coli*, with a profile that distinguishes it from conventional fast-acting antibiotics. These findings support further investigation into its mechanism of action and in vivo efficacy.

#### 3.2.3. Inhibition of Biofilm Formation

**Figure 6:**
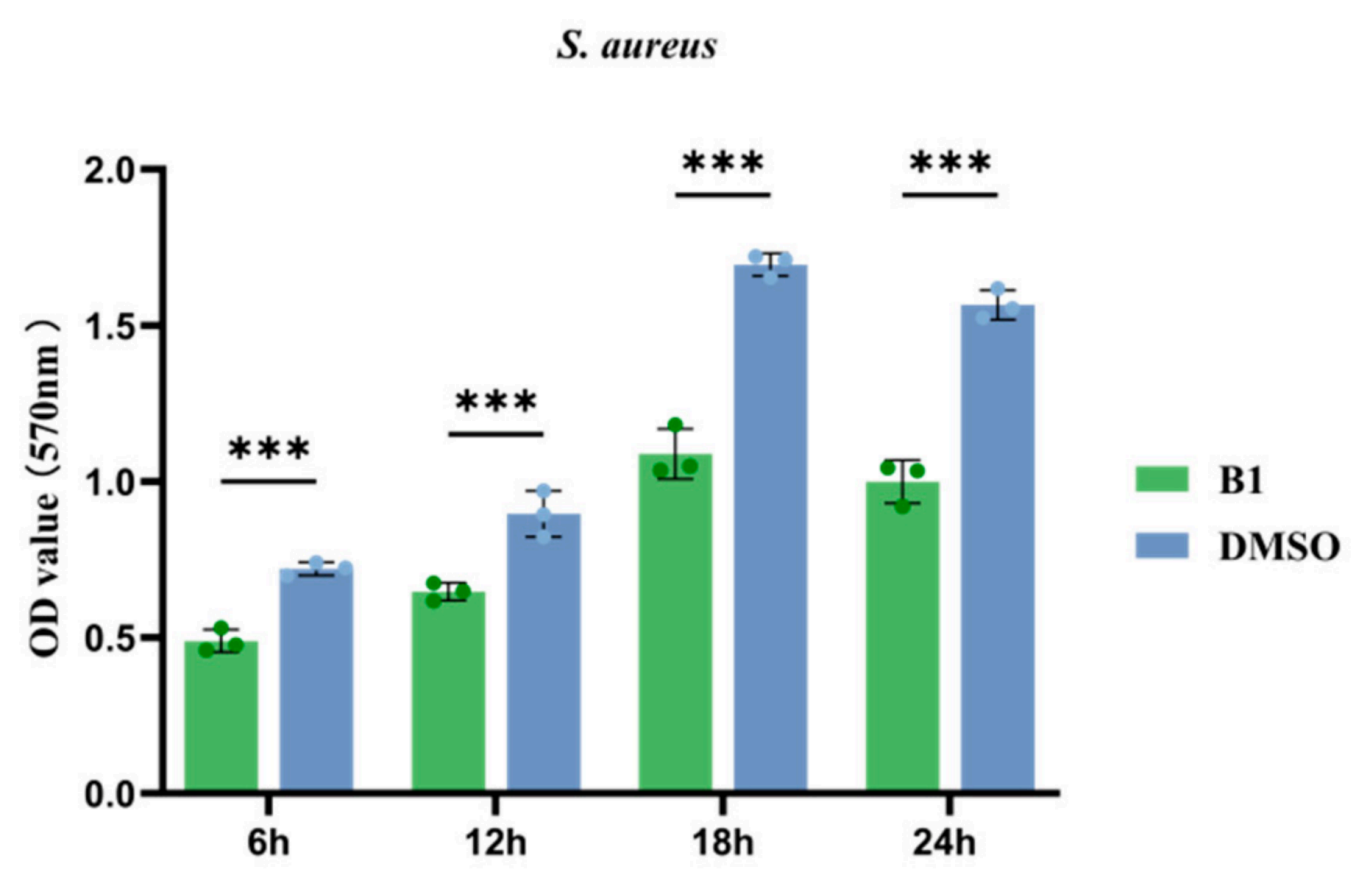
Inhibitory effect of compound **B1** on biofilm formation of *S. aureus*. *** *p* < 0.001.

**Figure 7:**
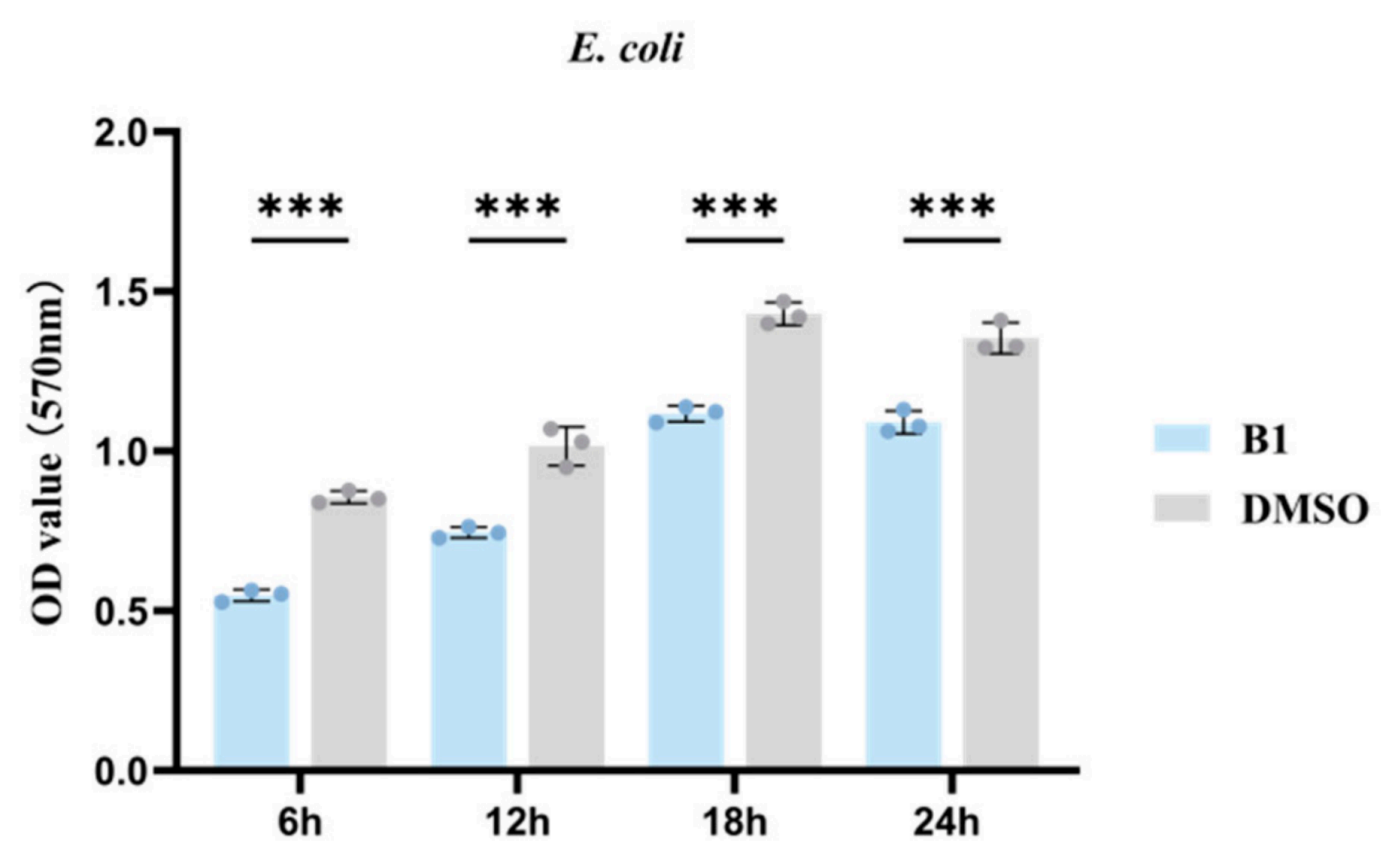
Inhibitory effect of compound **B1** on biofilm formation of *E. coli*. *** *p* < 0.001.

Given that compound **B1** exhibited potent and sustained bactericidal activity against planktonic *S. aureus* and *E. coli*, we next investigated its ability to interfere with biofilm formation—a key virulence factor associated with chronic infections and antibiotic tolerance. The effect of **B1** on biofilm formation was evaluated using the crystal violet staining method at different time points (6, 12, 18, and 24 h) under a concentration of 150 μg/mL. All experiments were performed in triplicate (*n* = 3), and data were analyzed using one-way ANOVA. As shown in Figure 6 and Figure 7, treatment with **B1** significantly reduced biofilm biomass in both bacterial strains at all tested time points compared to the untreated control (*p* < 0.001). The inhibitory effect was already evident at 6 h and persisted throughout the 24 h incubation period. These results indicate that **B1** not only suppresses the growth of planktonic bacteria but also effectively disrupts the establishment of biofilms. Given the critical role of the SarA protein in regulating biofilm formation in *S. aureus*, these findings prompted us to explore the molecular interaction between **B1** and SarA through docking studies.

#### 3.2.4. Molecular Docking

**Figure 8:**
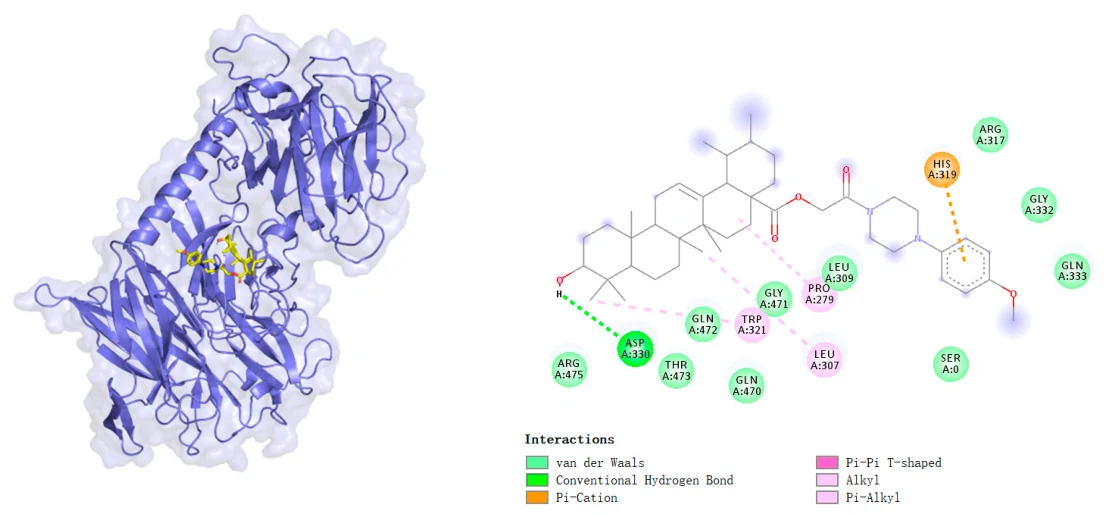
Interaction mode of target compound **B1** with SarA protein (PDB: 2FHR).

The staphylococcal accessory regulator A (SarA) is a well-established global transcriptional regulator that controls the expression of numerous virulence factors in *S. aureus*, including those essential for biofilm formation [34]. Given that compound **B1** significantly inhibited *S. aureus* biofilm formation, we hypothesized that SarA might be a potential molecular target of **B1**. To test this hypothesis and to gain structural insights into the binding mode, molecular docking was performed using AutoDock Vina. The crystal structure of SarA (PDB: 2FHR) was used as the receptor. As shown in Figure 8, **B1** spontaneously bound to the active pocket of SarA, with a calculated binding energy of −6.8 kcal/mol, indicating a favorable interaction. Detailed analysis of the docking pose revealed that the binding is stabilized by a hydrogen bond formed with ASP-330. Additionally, the benzene ring moiety of **B1** engaged in a π-cation interaction with HIS-319. Furthermore, the ursolic acid moiety of **B1** participated in alkyl and π-alkyl interactions with adjacent residues, further enhancing the stability of the complex. These specific and strong interactions provide a plausible structural basis for the observed anti-biofilm activity of **B1**. Moreover, the docking results suggest that **B1** may exert its antibacterial effect, at least in part, by modulating SarA function, thereby downregulating the expression of biofilm-associated genes. To further validate the stability of the protein–ligand complex and to explore the dynamic behavior of the interaction, a 100 ns molecular dynamics simulation was subsequently performed.

#### 3.2.5. Molecular Dynamics Simulation

**Figure 9:**
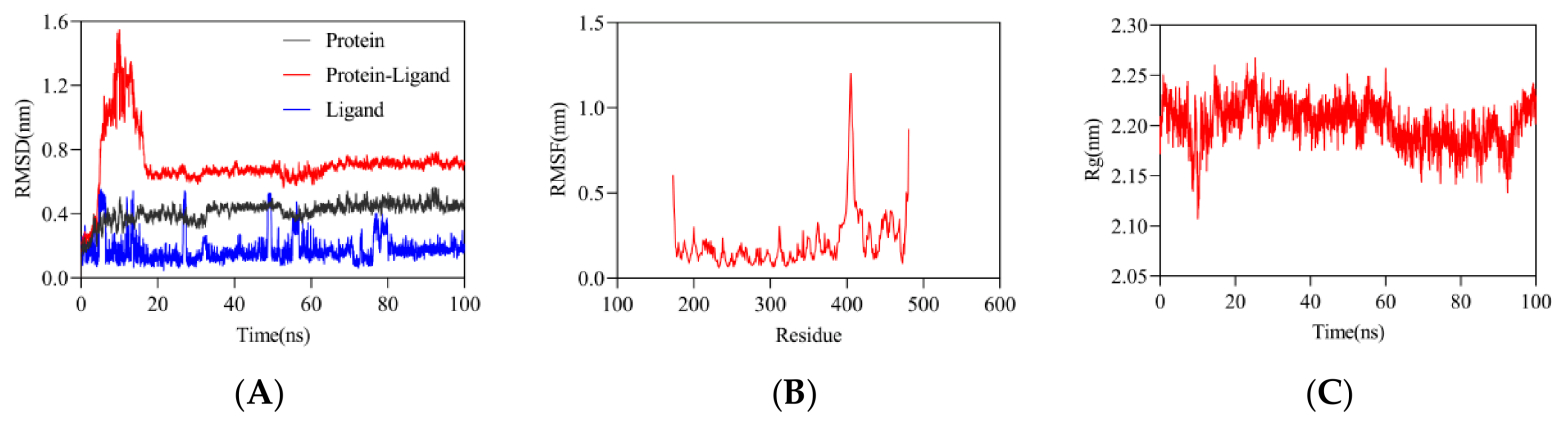

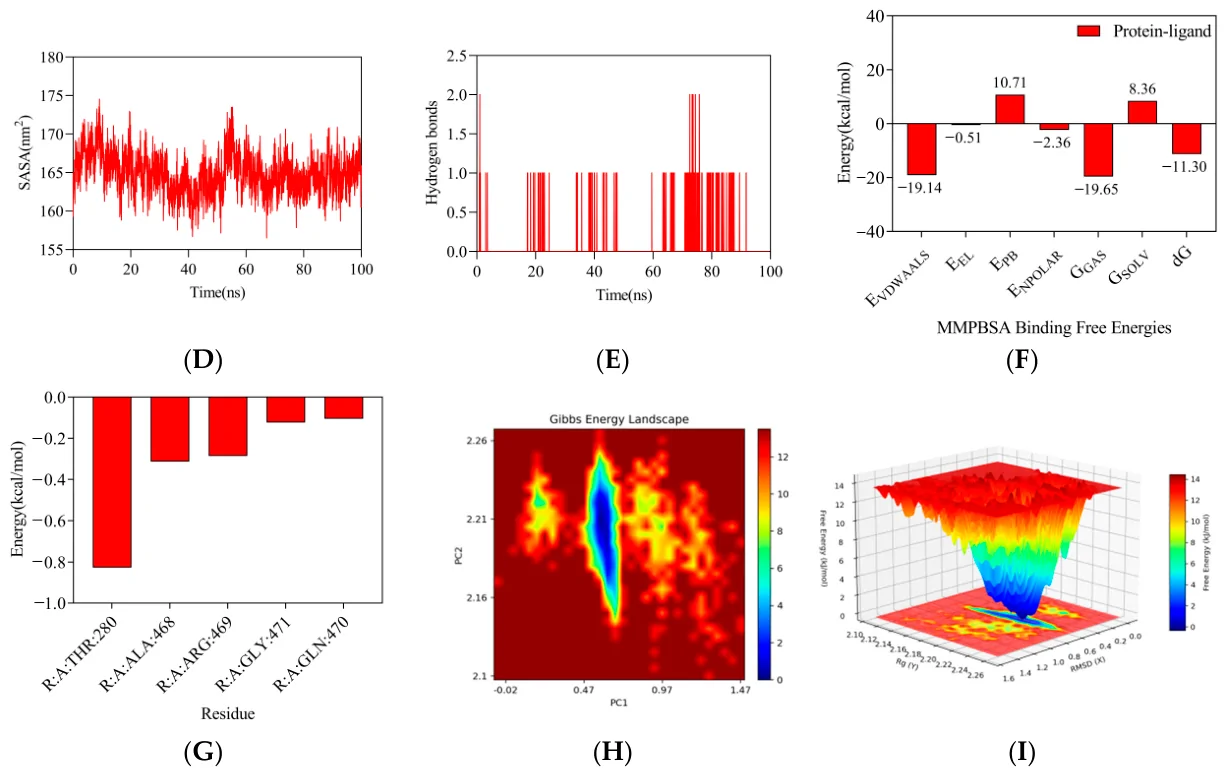
Binding stability and conformational dynamics of compound **B1** from molecular dynamics simulations. (**A**) Root-mean-square deviation (RMSD) of the protein backbone. (**B**) Residue-wise root-mean-square fluctuation (RMSF). (**C**) Radius of gyration (Rg) of the protein. (**D**) Solvent-accessible surface area (SASA). (**E**) Time course of protein–ligand hydrogen bonds. (**F**) Binding free energy calculated by the MM-PBSA method. (**G**) Binding energy decomposition for key residues. (**H**,**I**) Two-dimensional and three-dimensional conformational free energy landscapes.

Based on the molecular docking results, a 100 ns molecular dynamics (MD) simulation of the SarA (PDB: 2FRH)-ligand (**B1**) complex was conducted using GROMACS 2025. The RMSD values plateaued after 10 ns, indicating that the protein reached a stable state upon ligand binding. The absence of significant ligand movement within the binding site suggests a stable binding mode (Figure 9A). Most residues exhibited low RMSF values, reflecting the structural stability of the protein, whereas the RMSF at the C-terminus was higher, which may correspond to a flexible region of the protein (Figure 9B). After 10 ns, the radius of gyration (Rg) decreased and subsequently plateaued, suggesting that the protein adopted a more compact conformation upon ligand binding (Figure 9C). The decrease in solvent-accessible surface area (SASA) after 10 ns indicated tighter packing of the protein around the ligand, further corroborating the stability of the complex (Figure 9D). After 10 ns, the number of hydrogen bonds plateaued, indicating consistent and favorable protein–ligand interactions throughout the simulation (Figure 9E). With a total binding free energy of −11.30 kcal/mol, the ligand exhibits spontaneous binding to the protein, and the complex is thermodynamically stable, providing an energetic basis for further investigation of the biological functions of this ligand-protein complex (Figure 9F). Residues such as THR-280, ALA-468, and ARG-469 exhibited small negative energy values, indicating that these residues contribute relatively strongly to the stability of the protein–ligand interaction (Figure 9G). As shown in the free energy landscape, deep blue basins represented highly populated, low-energy conformational states of the bound complex. In contrast, yellow/red regions signified energy barriers or transition states between different conformational clusters, reflecting the dynamic bottlenecks encountered during the sampling process (Figure 9H,I). In summary, these data confirm that compound **B1** interacts with SarA with strong affinity, a conclusion supported by the persistent structural stability throughout the simulation and the energetically favorable binding.

### 3.3. Discussion

The results of this study demonstrate that structural modification of ursolic acid at the C-28 position via a chloroacetamide linker with piperazine moieties significantly enhances its antibacterial and anti-biofilm effects against both Gram-positive (*S. aureus*) and Gram-negative (*E. coli*) pathogens. Our synthetic strategy systematically explored three distinct classes of functional groups—1,3,4-thiadiazole amides (**A1**–**A7**), piperazines (**B1**–**B12**), and aniline/aromatic heterocycles (**B13**–**B19**)—revealing a clear structure–activity relationship (SAR) trend: piperazine-modified derivatives consistently outperformed the other two classes, with compound **B1** emerging as the most promising candidate. The strategic introduction of a chloroacetamide linker combined with a 4-methoxyphenyl piperazine moiety proved to be a productive approach for enhancing antibacterial activity, suggesting that the combination of a flexible spacer with a basic nitrogen-containing heterocycle at C-28 is particularly favorable for target engagement.

A comparison with alternative C-28 modification strategies in the field provides a clearer context for the novelty and value of our work. Khwaza et al. synthesized a series of amine-linked ursolic acid hybrids, with representative compounds exhibiting MIC values ranging from 15.3 to 31.25 μg/mL against various bacterial strains [16]. In contrast, our approach employed a chloroacetamide linker, which provides additional conformational flexibility and hydrogen-bonding capacity that may enhance target engagement. More recently, Sun et al. reported a series of UA derivatives incorporating benzenesulfonamide and indole pharmacophores at the C-28 position that exhibited potent anti-MRSA activity with MIC values as low as 1 μM, accompanied by significant biofilm inhibition and low hemolytic toxicity [35]. While these derivatives show superior direct antibacterial potency compared to our compound **B1**, our approach offers a distinct mechanism of action targeting SarA, a virulence regulator rather than essential cellular processes. This anti-virulence strategy aligns with recent paradigm shifts in antibacterial drug discovery that prioritize target specificity and resistance avoidance over direct bactericidal potency alone [34]. Furthermore, Liu et al. synthesized 1,3,4-thiadiazole derivatives containing sulfonylpiperazine structures and reported moderate to good antibacterial activities [21]. However, in our study, the 1,3,4-thiadiazole amide derivatives (**A1**–**A7**) did not exhibit significant antibacterial activity (MIC > 150 μg/mL), suggesting that the direct conjugation of this heterocycle to the UA scaffold via a chloroacetamide linker is less favorable for antibacterial efficacy compared to the piperazine modification. This discrepancy highlights the critical role of the piperazine pharmacophore in our system and underscores the importance of systematic SAR exploration.

The SAR analysis of our piperazine derivatives (**B1**–**B12**) reveals several important trends. First, the introduction of the piperazine ring itself appears to play a dual role: the basic nitrogen atoms can form hydrogen bonds with bacterial target proteins, while the *N*-aryl substituent contributes hydrophobic interactions that may enhance membrane affinity and target engagement [23]. Second, among the various *N*-aryl substituents evaluated, the 4-methoxyphenyl group in **B1** provided the most favorable antibacterial profile (MIC = 37.5 μg/mL against *S. aureus* and 75 μg/mL against *E. coli*). The markedly reduced activity observed for compounds lacking this methoxy substitution, such as **B7** (4-phenyl, MIC > 150 μg/mL), further supports the importance of this aryl moiety for optimal antibacterial efficacy. Third, the position and nature of substituents on the phenyl ring significantly influenced activity; compounds with electron-withdrawing groups such as nitro (**B10**) or cyano (**B11**) showed only modest activity, whereas the electron-donating methoxy group at the para position (**B1**) was most beneficial. This trend suggests that electronic effects play a critical role in modulating target interactions. Fourth, the aniline/aromatic heterocyclic derivatives (**B13**–**B19**) generally exhibited weaker activity compared to the piperazine series, with only **B14** (2,5-difluoroaniline) showing moderate activity against *S. aureus* (MIC = 75 μg/mL), indicating that the piperazine ring confers unique advantages over simple aniline substitutions.

Time-kill kinetics indicated that compound **B1** produced a sustained reduction in viable counts (1.72 log_10_ CFU/mL for *S. aureus* and 1.33 log_10_ CFU/mL for *E. coli*) over 24 h without regrowth. Although this reduction did not meet the conventional 3 log_10_ CFU/mL criterion for strict bactericidal classification, the consistent and progressive decline in bacterial viability, coupled with the absence of regrowth, suggests that **B1** may offer advantages over rapidly bactericidal agents by potentially reducing the selective pressure that drives resistance development. This sustained killing profile is reminiscent of some pentacyclic triterpenoid derivatives whose primary strength lies in inhibiting virulence and biofilm formation rather than causing rapid bactericidal effects [15]. The pronounced and consistent suppression of biofilm formation by **B1** across all tested time points (6, 12, 18, and 24 h) represents a significant finding. Its anti-biofilm efficacy compares favorably with other recently described natural product derivatives targeting staphylococcal biofilms, such as certain UA conjugates that act via membrane disruption [15], and hints at a potentially distinct mechanism. This feature is of considerable therapeutic value given the critical role of biofilms in chronic infections and antibiotic tolerance.

Mechanistically, our molecular docking and 100 ns molecular dynamics simulations provided compelling evidence that **B1** binds spontaneously to the staphylococcal accessory regulator A (SarA), a global transcriptional regulator that positively controls biofilm formation and virulence gene expression in *S. aureus* [34]. The calculated binding free energy of −11.30 kcal/mol, together with the sustained structural stability of the SarA-**B1** complex throughout the simulation (as evidenced by plateaued RMSD and Rg values), supports the hypothesis that **B1** exerts its anti-biofilm effect, at least in part, through direct modulation of SarA function. This finding is particularly significant, as SarA has emerged as a promising antivirulence target for combating drug-resistant staphylococcal infections [34]. Notably, recent studies on C-28 modified betulinic acid derivatives—another class of pentacyclic triterpenoids structurally related to UA—have similarly identified SarA as a key target for anti-biofilm activity [32], suggesting that C-28 functionalized triterpenoids may represent a general scaffold for developing SarA-targeted anti-biofilm agents. Our integrated computational studies propose a novel mechanism involving interference with SarA, a key virulence controller less commonly targeted by traditional triterpenoid-based antibacterials. This virulence-targeting mode could offer a strategic advantage by potentially reducing the selection pressure for conventional resistance development. To our knowledge, this is the first report that a C-28 piperazine-modified UA derivative can effectively engage SarA.

Nevertheless, the limited activity of our compound series against *P. aeruginosa* (MIC > 150 μg/mL) highlights a common limitation of many triterpenoid derivatives, likely attributable to the impermeable outer membrane and/or efficient efflux systems in Gram-negative pathogens [16,32]. This observation is consistent with previous reports on UA and betulinic acid derivatives, which typically exhibit preferential activity against Gram-positive bacteria. The lack of activity against Gram-negative strains underscores the challenge of modifying pentacyclic triterpenoids to achieve broad-spectrum activity and suggests that alternative strategies, such as combination with membrane-permeabilizing agents or formulation in nanoparticle delivery systems, may be required to overcome this limitation.

Looking forward, this study points to several promising directions. First, comprehensive in vitro cytotoxicity profiling against mammalian cell lines and hemolytic assays are essential next steps to establish a therapeutic window for lead compound **B1**. Second, given its anti-biofilm activity and the proposed SarA-targeting mechanism, combination therapy studies with conventional antibiotics (e.g., tetracycline, vancomycin) are warranted to explore potential synergy in eradicating established biofilms or treating persistent infections [34]. Third, the proposed mechanism of SarA inhibition requires further genetic validation—for example, using sarA-deletion mutant strains to confirm the specificity of the anti-biofilm effect or conducting transcriptomic analyses to assess downstream changes in virulence gene expression. Fourth, structural optimization could focus on improving potency and broadening the spectrum through systematic variation in the piperazine *N*-aryl substituents, exploration of additional heterocyclic scaffolds, and comprehensive in vivo efficacy and safety evaluations. The chloroacetamide linker strategy employed in this study provides a versatile platform for such optimization. Finally, advancing the most promising lead into in vivo efficacy models of biofilm-associated infection would represent a crucial milestone in preclinical development.

In summary, the C-28 chloroacetamide-modified piperazine functionalization strategy described herein offers a fruitful avenue for developing dual-action antibacterial and anti-biofilm agents. Compound **B1** serves as a valuable lead candidate, demonstrating potent activity against both *S. aureus* and *E. coli*, significantly inhibiting biofilm formation, and engaging SarA as a potential molecular target. Different structural modification strategies—piperazine incorporation, aniline substitution, thiadiazole conjugation—each possess distinct advantages; our work demonstrates that the piperazine-chloroacetamide combination is particularly effective within the UA scaffold. Collectively, these results establish C-28 piperazine-modified ursolic acid derivatives as a promising class of antibacterial agents warranting further preclinical development, and underscore the continued value of natural product structural modification in addressing the urgent global challenge of antimicrobial resistance [16,18].

## 4. Conclusions

In this study, we successfully designed and synthesized 26 novel ursolic acid derivatives. Among them, the piperazine derivative **B1** exhibited the most potent and sustained antibacterial activity against *S. aureus* and *E. coli*, significantly inhibited biofilm formation, and demonstrated favorable binding to the SarA protein in molecular docking. These findings highlight the potential of C-28 piperazine-modified UA derivatives as a new class of antibacterial agents and provide a promising lead for further development. The work also underscores the utility of structural modification of natural products in addressing the urgent challenge of antimicrobial resistance.

## Figures and Tables

**Table 1:** MIC values (μg/mL) of active UA derivatives against tested bacterial strains.

Compd.	*S. aureus*	*S. epidermidis*	*E. coli*	*P. aeruginosa*
	ATCC25923	ATCC12228	ATCC25922	ATCC27853
**B1**	37.5	>150	75	>150
**B10**	150	150	>150	>150
**B14**	75	>150	150	>150
Ursolic Acid	>150	>150	>150	>150
Tetracycline	1.17	18.75	2.34	18.75

## Data Availability

The original contributions presented in this study are included in the article/Supplementary Material. Further inquiries can be directed to the corresponding authors.

## Supplementary Materials

The following supporting information can be downloaded at: https://www.mdpi.com/article/10.3390/microorganisms14081685/s1, Figure S1: (1-1): ^1^H NMR spectrum of compound **A1**; (1-2): ^13^C NMR spectrum of compound **A1**; (1-3): HRMS spectrum of compound **A1**; Figure S2: (2-1): ^1^H NMR spectrum of compound **A2**; (2-2): ^13^C NMR spectrum of compound **A2**; (2-3): HRMS spectrum of compound **A2**; Figure S3: (3-1): ^1^H NMR spectrum of compound **A3**; (3-2): ^13^C NMR spectrum of compound **A3**; (3-3): HRMS spectrum of compound **A3**; Figure S4: (4-1): ^1^H NMR spectrum of compound **A4**; (4-2): ^13^C NMR spectrum of compound **A4**; (4-3): HRMS spectrum of compound **A4**; Figure S5: (5-1): ^1^H NMR spectrum of compound **A5**; (5-2): ^13^C NMR spectrum of compound **A5**; (5-3): HRMS spectrum of compound **A5**; Figure S6: (6-1): ^1^H NMR spectrum of compound **A6**; (6-2): ^13^C NMR spectrum of compound **A6**; (6-3): HRMS spectrum of compound **A6**; Figure S7: (7-1): ^1^H NMR spectrum of compound **A7**; (7-2): ^13^C NMR spectrum of compound **A7**; (7-3): HRMS spectrum of compound **A7**; Figure S8: (8-1): ^1^H NMR spectrum of compound **B1**; (8-2): ^13^C NMR spectrum of compound **B1**; (8-3): HRMS spectrum of compound **B1**; Figure S9: (9-1): ^1^H NMR spectrum of compound **B2**; (9-2): ^13^C NMR spectrum of compound **B2**; (9-3): HRMS spectrum of compound **B2**; Figure S10: (10-1): ^1^H NMR spectrum of compound **B3**; (10-2): ^13^C NMR spectrum of compound **B3**; (10-3): HRMS spectrum of compound **B3**; Figure S11: (11-1): ^1^H NMR spectrum of compound **B4**; (11-2): ^13^C NMR spectrum of compound **B4**; (11-3): HRMS spectrum of compound **B4**; Figure S12: (12-1): ^1^H NMR spectrum of compound **B5**; (12-2): ^13^C NMR spectrum of compound **B5**; (12-3): HRMS spectrum of compound **B5**; Figure S13: (13-1): ^1^H NMR spectrum of compound **B6**; (13-2): ^13^C NMR spectrum of compound **B6**; (13-3): HRMS spectrum of compound **B6**; Figure S14: (14-1): ^1^H NMR spectrum of compound **B7**; (14-2): ^13^C NMR spectrum of compound **B7**; (14-3): HRMS spectrum of compound **B7**; Figure S15: (15-1): ^1^H NMR spectrum of compound **B8**; (15-2): ^13^C NMR spectrum of compound **B8**; (15-3): HRMS spectrum of compound **B8**; Figure S16: (16-1): ^1^H NMR spectrum of compound **B9**; (16-2): ^13^C NMR spectrum of compound **B9**; (16-3): HRMS spectrum of compound **B9**; Figure S17: (17-1): ^1^H NMR spectrum of compound **B10**; (17-2): ^13^C NMR spectrum of compound **B10**; (17-3): HRMS spectrum of compound **B10**; Figure S18: (18-1): ^1^H NMR spectrum of compound **B11**; (18-2): ^13^C NMR spectrum of compound **B11**; (18-3): HRMS spectrum of compound **B11**; Figure S19: (19-1): ^1^H NMR spectrum of compound **B12**; (19-2): ^13^C NMR spectrum of compound **B12**; (19-3): HRMS spectrum of compound **B12**; Figure S20: (20-1): ^1^H NMR spectrum of compound **B13**; (20-2): ^13^C NMR spectrum of compound **B13**; (20-3): HRMS spectrum of compound **B13**; Figure S21: (21-1): ^1^H NMR spectrum of compound **B14**; (21-2): ^13^C NMR spectrum of compound **B14**; (21-3): HRMS spectrum of compound **B14**; Figure S22: (22-1): ^1^H NMR spectrum of compound **B15**; (22-2): ^13^C NMR spectrum of compound **B15**; (22-3): HRMS spectrum of compound **B15**; Figure S23: (23-1): ^1^H NMR spectrum of compound **B16**; (23-2): ^13^C NMR spectrum of compound **B16**; (23-3): HRMS spectrum of compound **B16**; Figure S24: (24-1): ^1^H NMR spectrum of compound **B17**; (24-2): ^13^C NMR spectrum of compound **B17**; (24-3): HRMS spectrum of compound **B17**; Figure S25: (25-1): ^1^H NMR spectrum of compound **B18**; (25-2): ^13^C NMR spectrum of compound **B18**; (25-3): HRMS spectrum of compound **B18**; Figure S26: (26-1): ^1^H NMR spectrum of compound **B19**; (26-2): ^13^C NMR spectrum of compound **B19**; (26-3): HRMS spectrum of compound **B19**.
