# Supplementary material for: Synthesis and Biological Evaluation of Novel C-28 Chloroacetamide-Modified Ursolic Acid Derivatives as Antibacterial Agents
Source: Microorganisms. 2026 Jul 31;14(8):1685. doi: 10.3390/microorganisms14081685 (PMC13515426; doi:10.3390/microorganisms14081685)
Supplement: Supplementary file 1 [file microorganisms-14-01685-s001.zip › microorganisms-4458977-supplementary.pdf]

Synthesis and Biological Evaluation of Novel C-28 Chloroacetamide-Modified Ursolic Acid Derivatives as Antibacterial Agents

Nan Cai [1, †], Tian Luan [1, †], Junchao Zhang [2], Ning Li [1], Xiu Zhang [1], Peng Gao [3], Jiaxuan Li [3], Hongyu Zhan [3], Fanhao Meng [2, *], Dajun Zhang [1, 2, *]

1. Shenyang Key Laboratory for Phenomics, Liaoning Province Key Laboratory for Phenomics of Human Ethnic Specificity and Critical Illness, Shenyang Medical College, Shenyang, China.
2. School of Pharmacy, China Medical University, Shenyang, China.
3. Faculty of Dentistry, The University of Hong Kong, Hong Kong.

Page 2-40: ^{1}H NMR, ^{13}C NMR, and HRMS spectra and formulas of target compounds **A1-A7** and **B1-B19**.

^{1}H NMR, ^{13}C NMR and HRMS spectra target compounds A1-A7 and B1-B19.

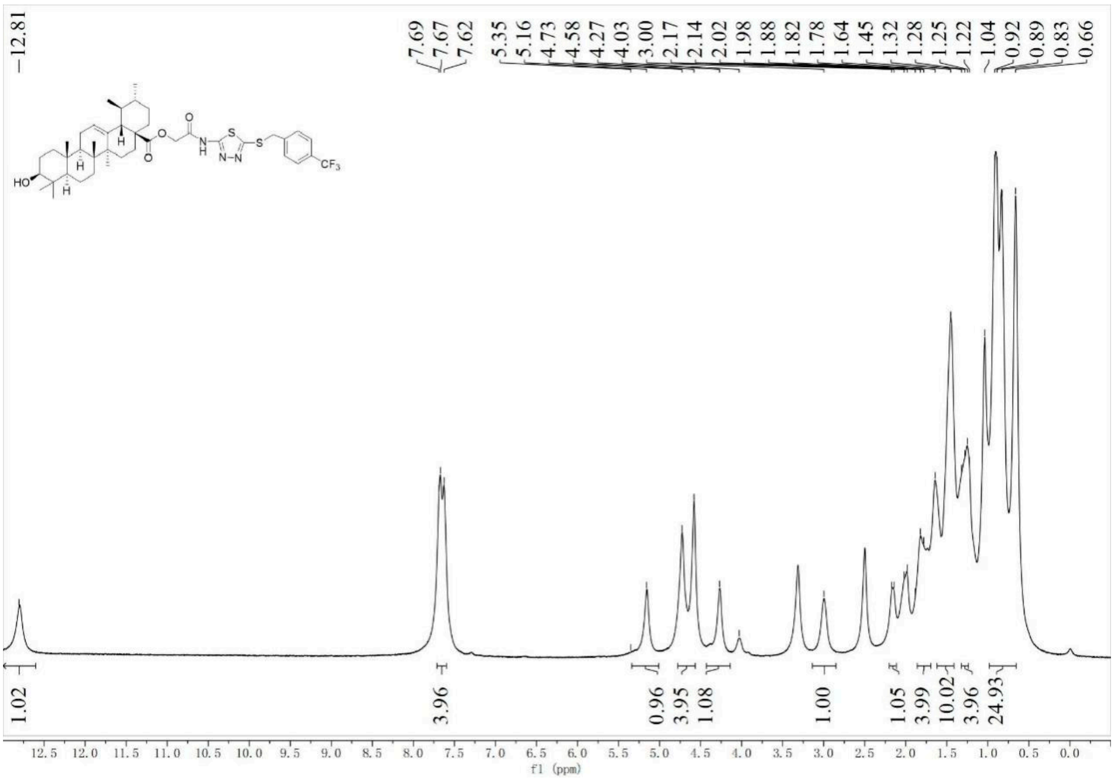

Figure S1-1 ^{1}H NMR spectrum of compound **A1**.

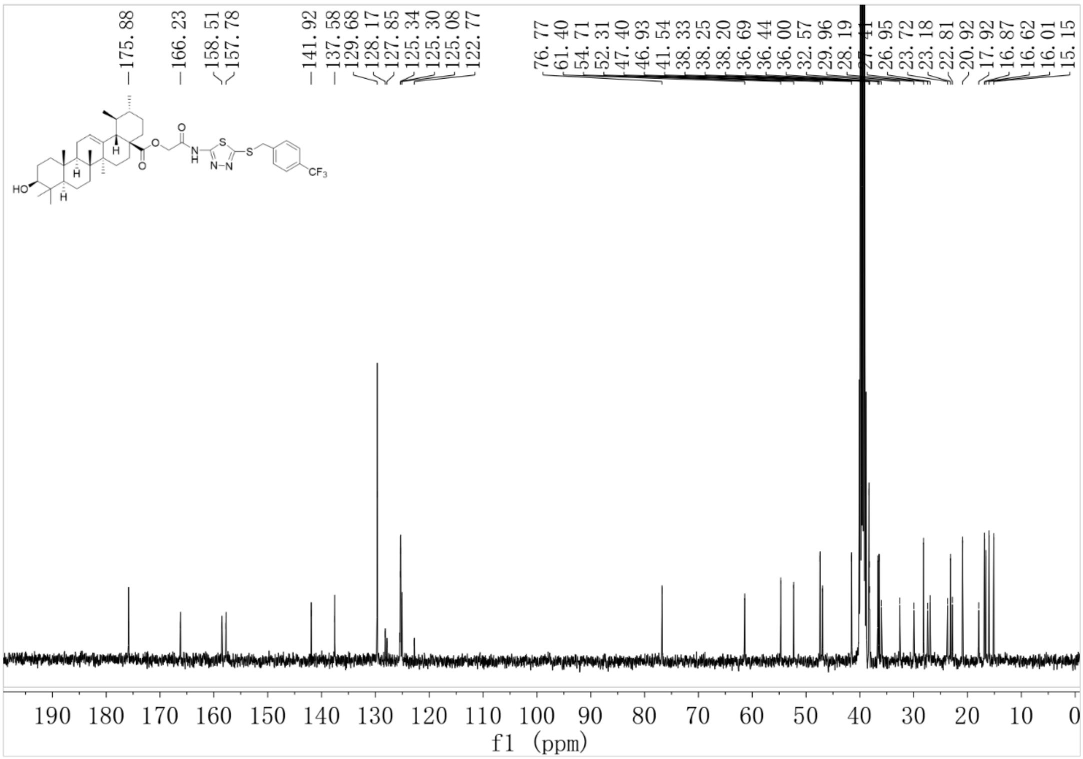

Figure S1-2 ^{13}C NMR spectrum of compound **A1.**

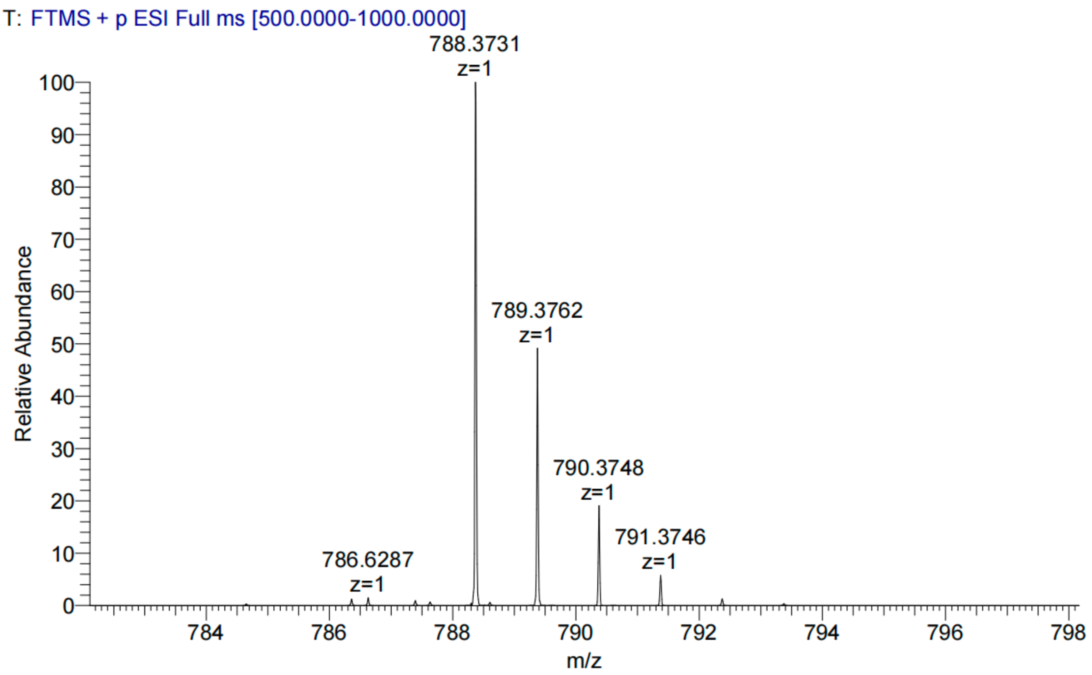

Figure S1-3 HRMS spectrum of compound **A1.**

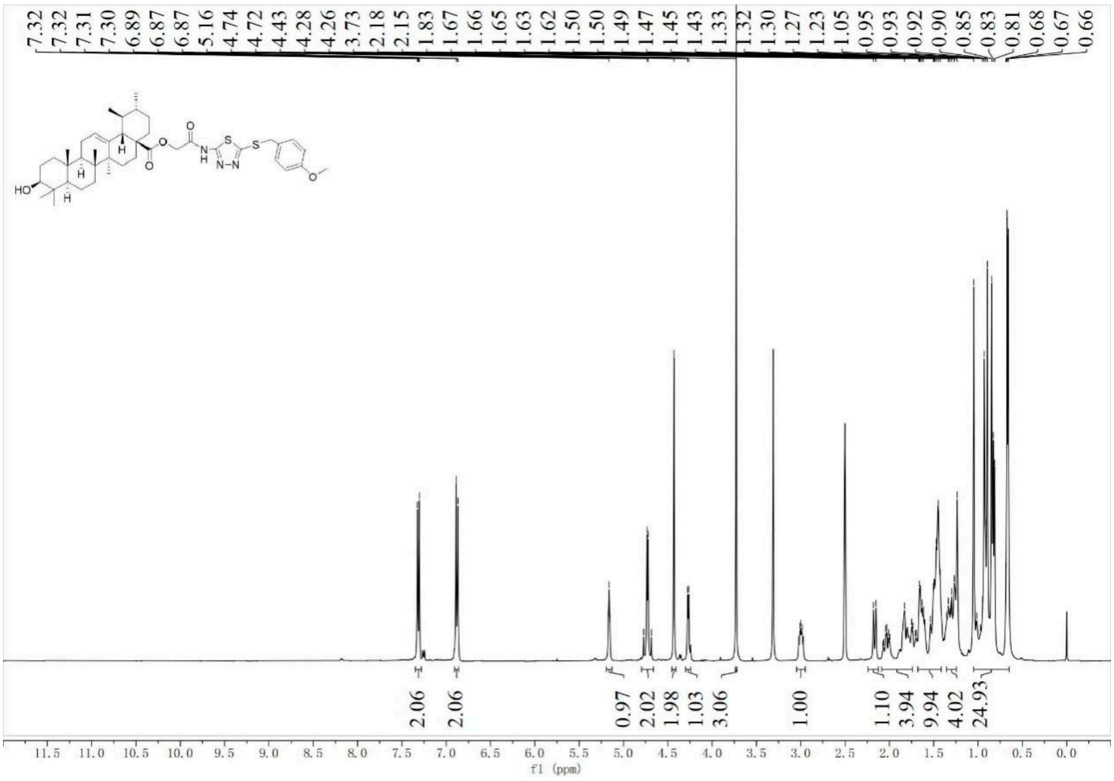

. Figure S2-1 ^{1}H NMR spectrum of compound **A2.**

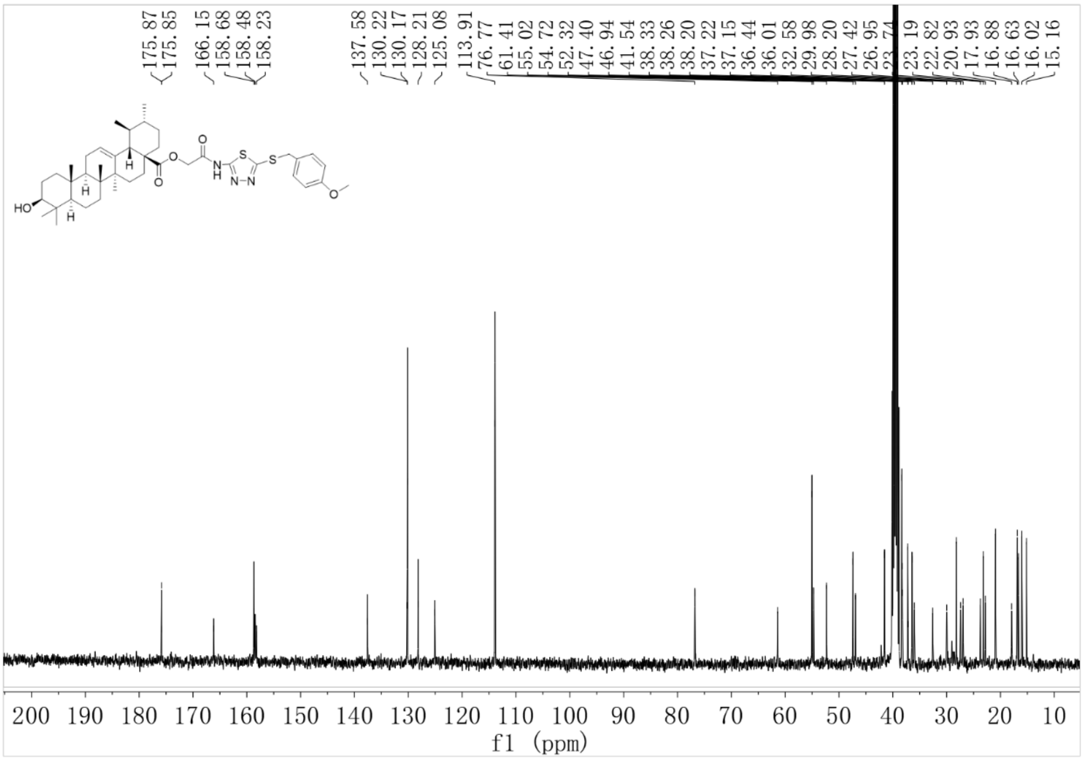

Figure S2-2 ^{13}C NMR spectrum of compound **A2.**

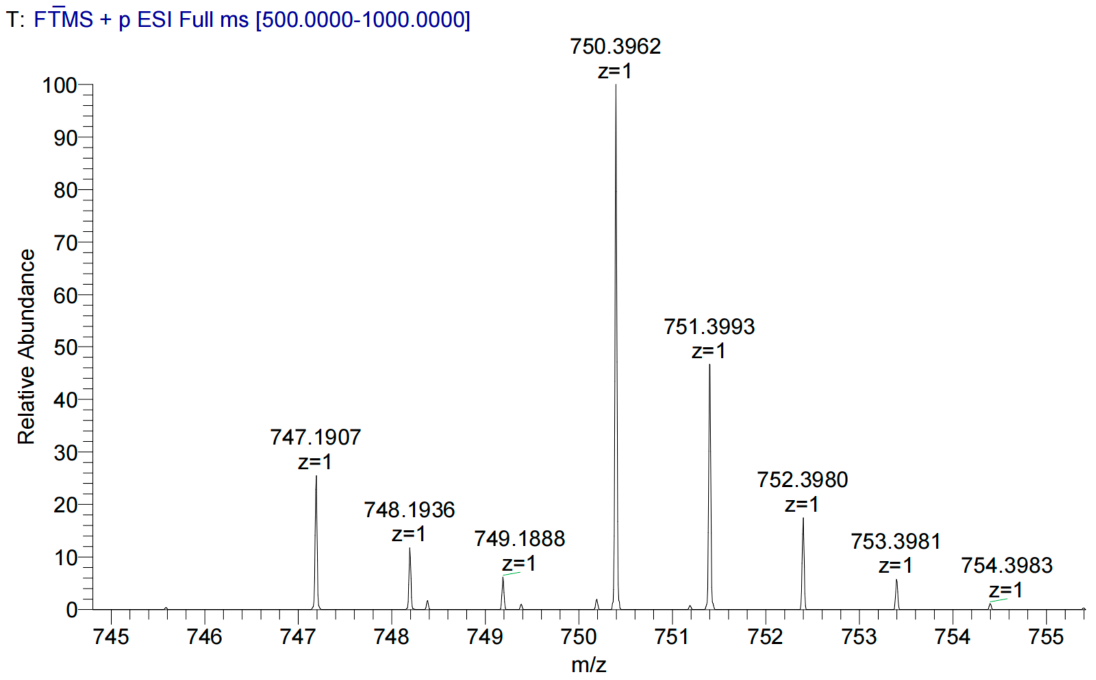

Figure S2-3 HRMS spectrum of compound **A2.**

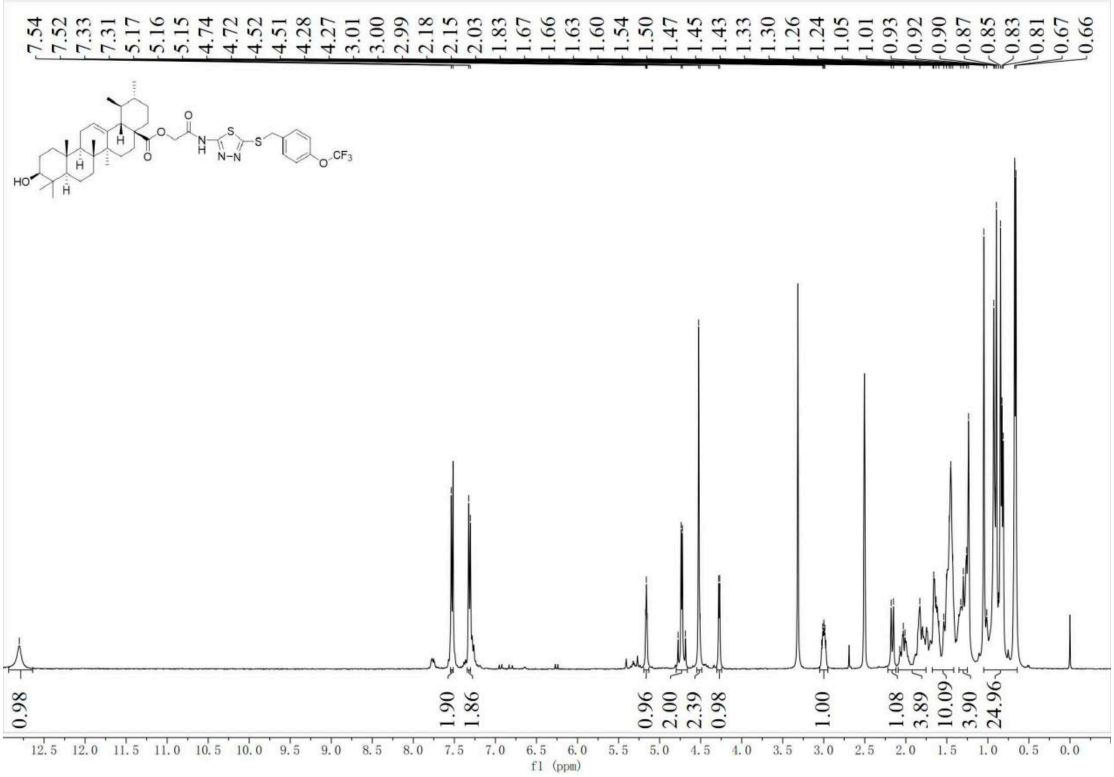

Figure S3-1 ^{1}H NMR spectrum of compound **A3.**

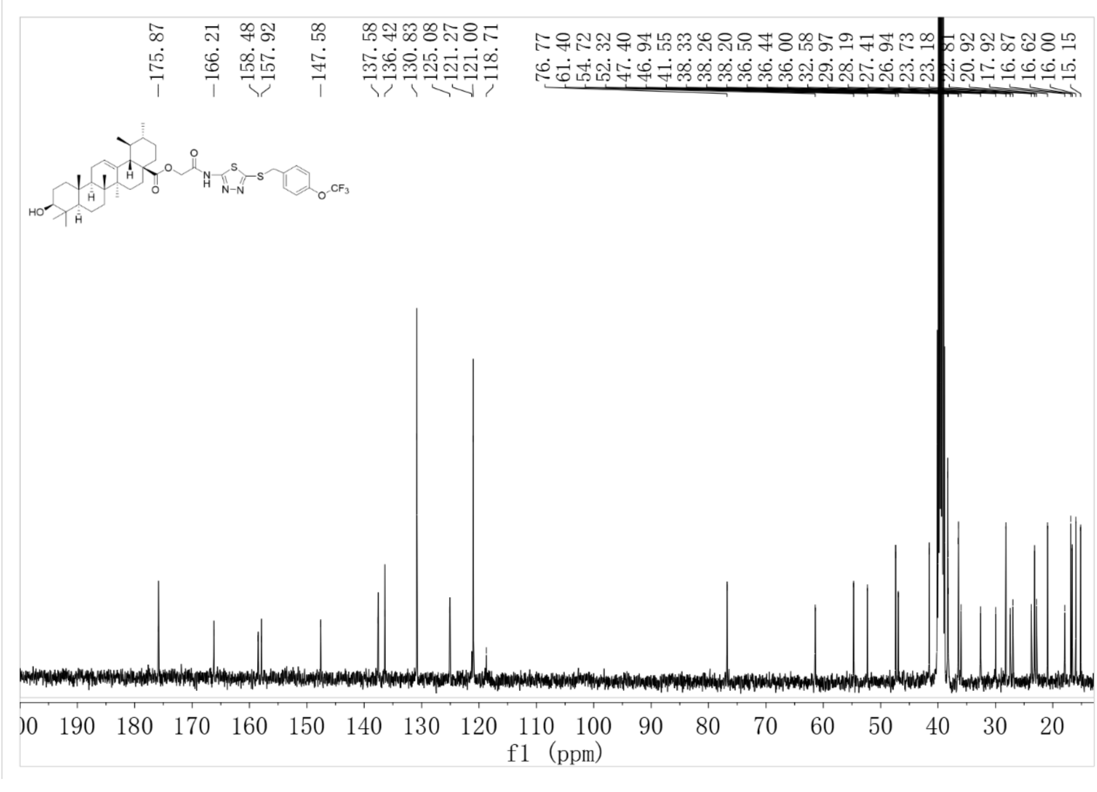

Figure S3-2 ^{13}C NMR spectrum of compound **A3.**

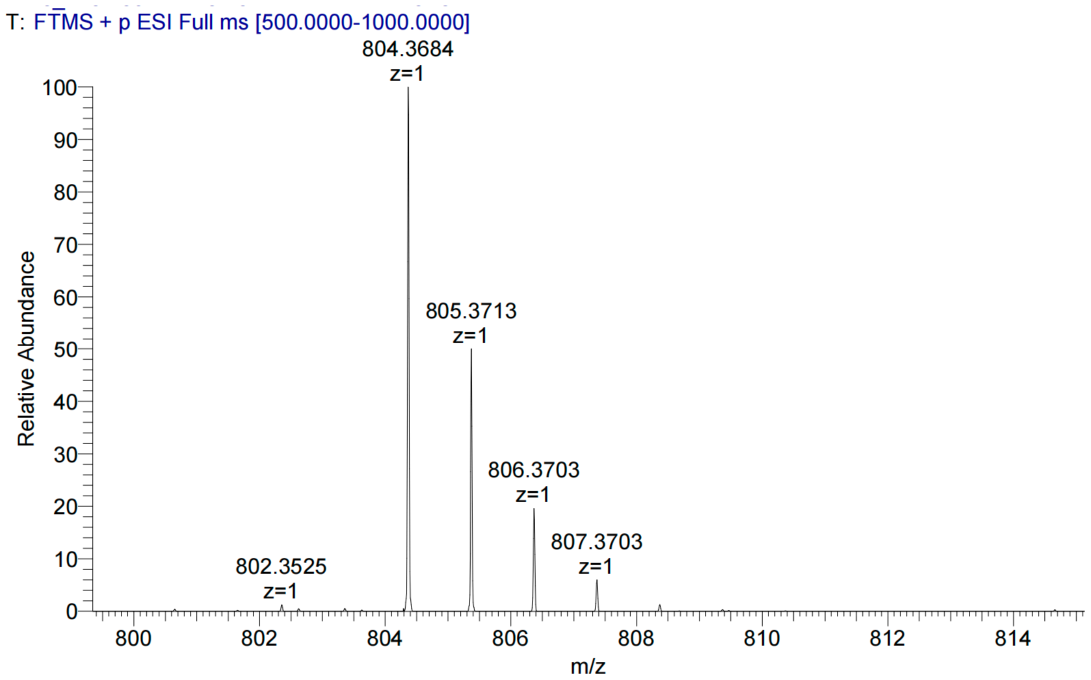

Figure S3-3 HRMS spectrum of compound **A3.**

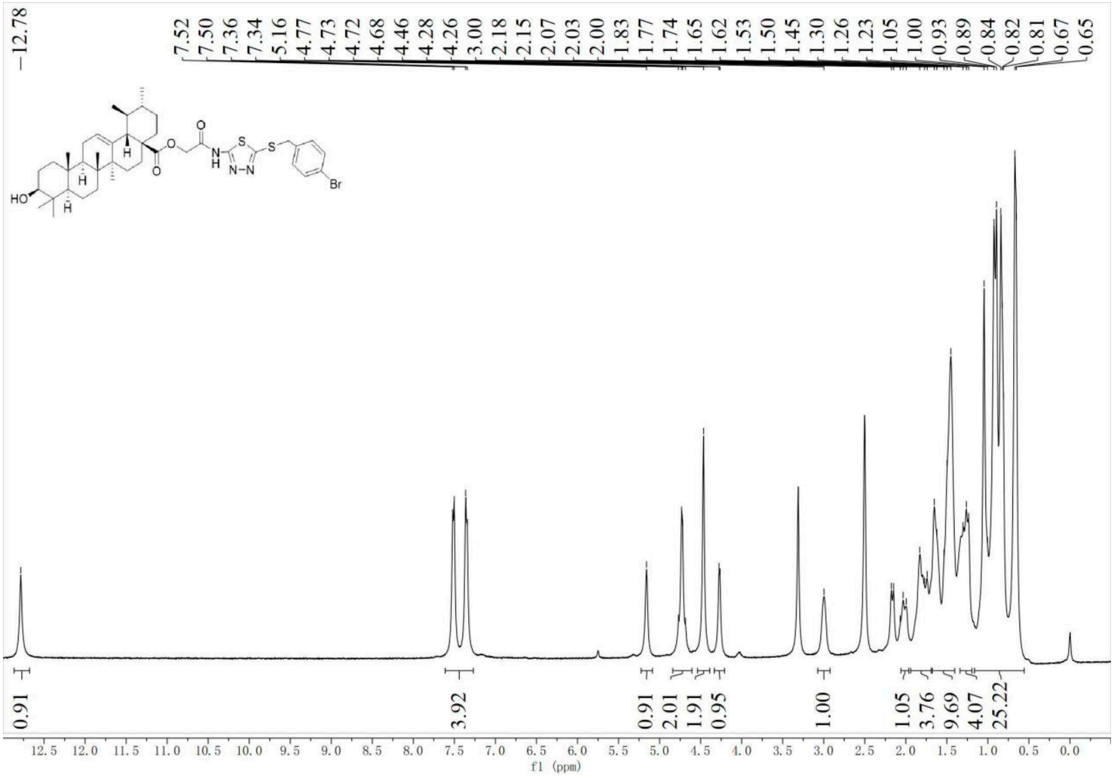

Figure S4-1 ^{1}H NMR spectrum of compound **A4.**

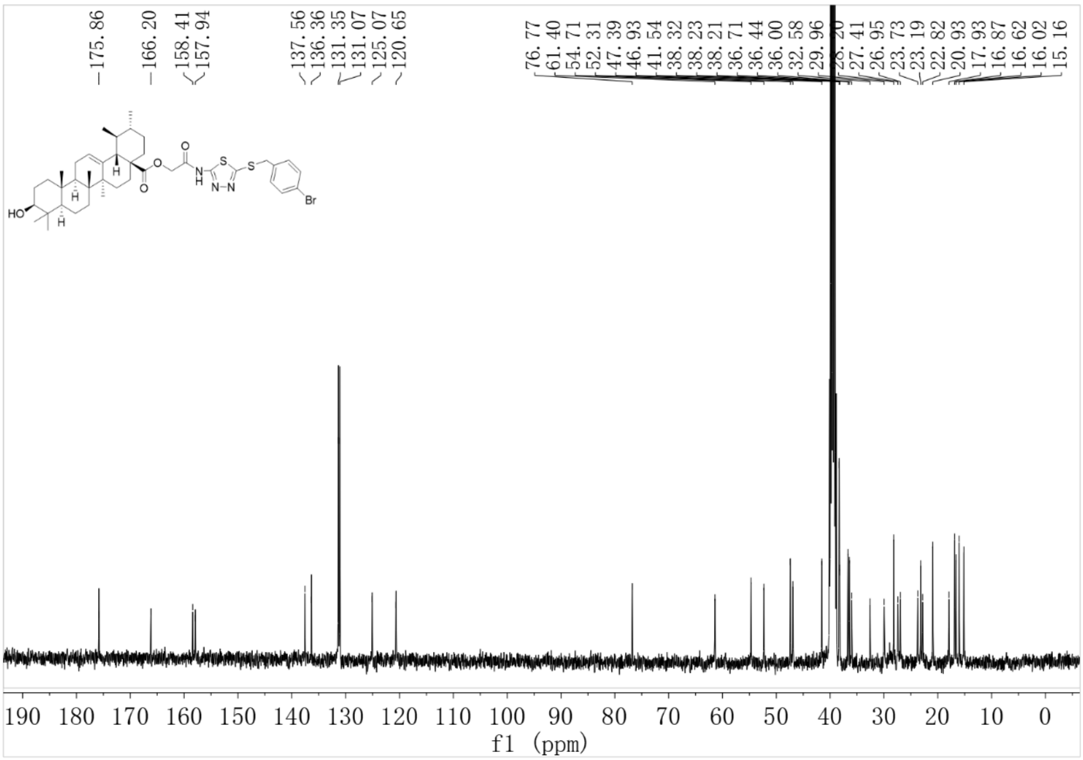

Figure S4-2 ^{13}C NMR spectrum of compound **A4.**

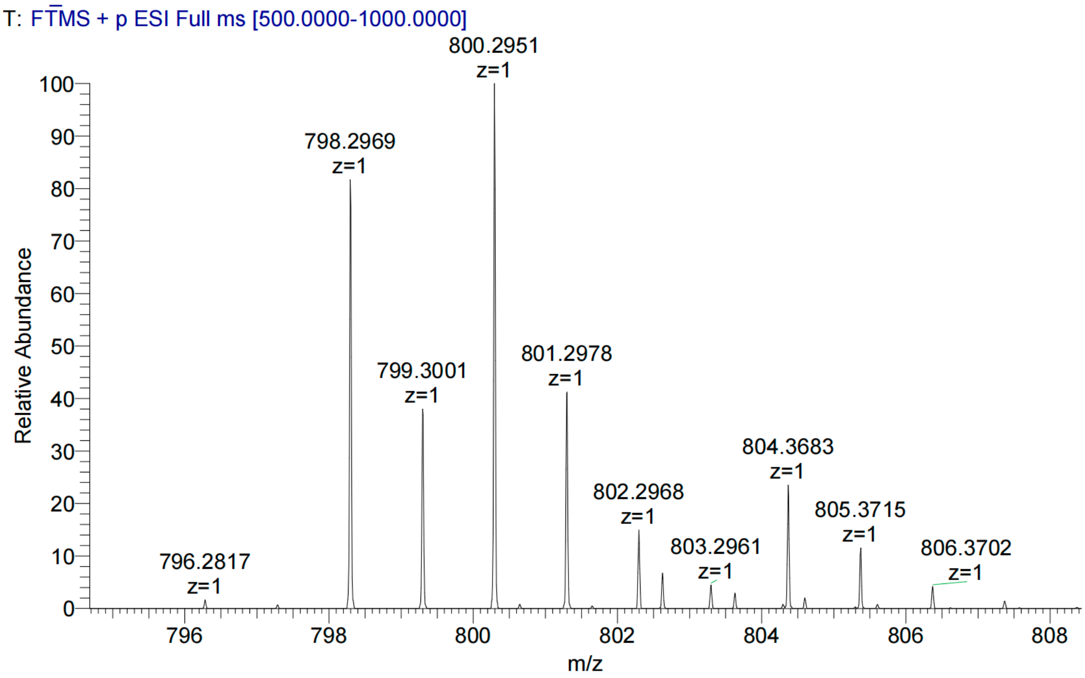

Figure S4-3 HRMS spectrum of compound **A4.**

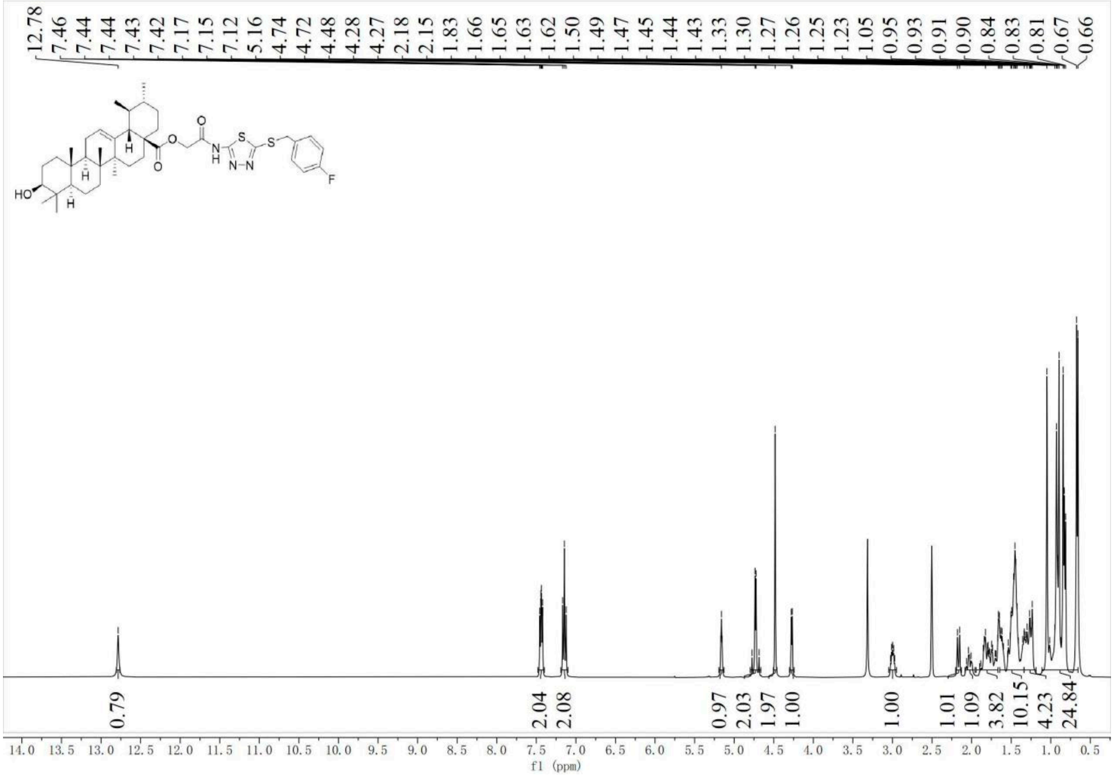

Figure S5-1 ^{1}H NMR spectrum of compound **A5.**

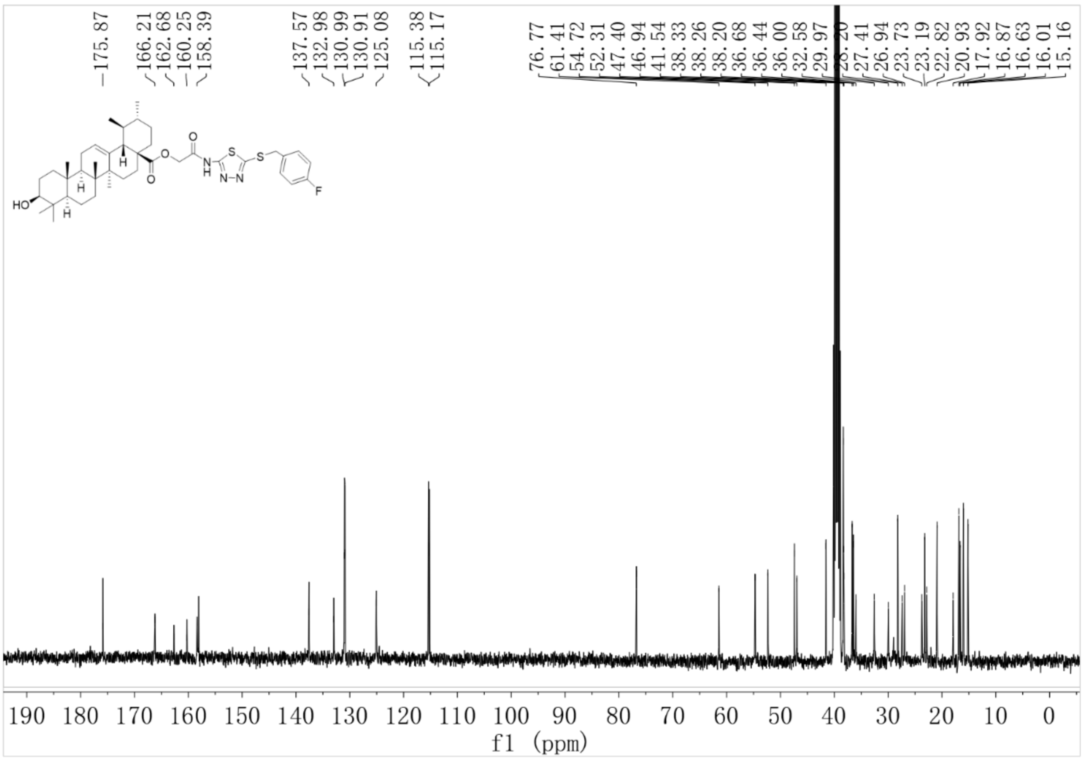

Figure S5-2 ^{13}C NMR spectrum of compound **A5.**

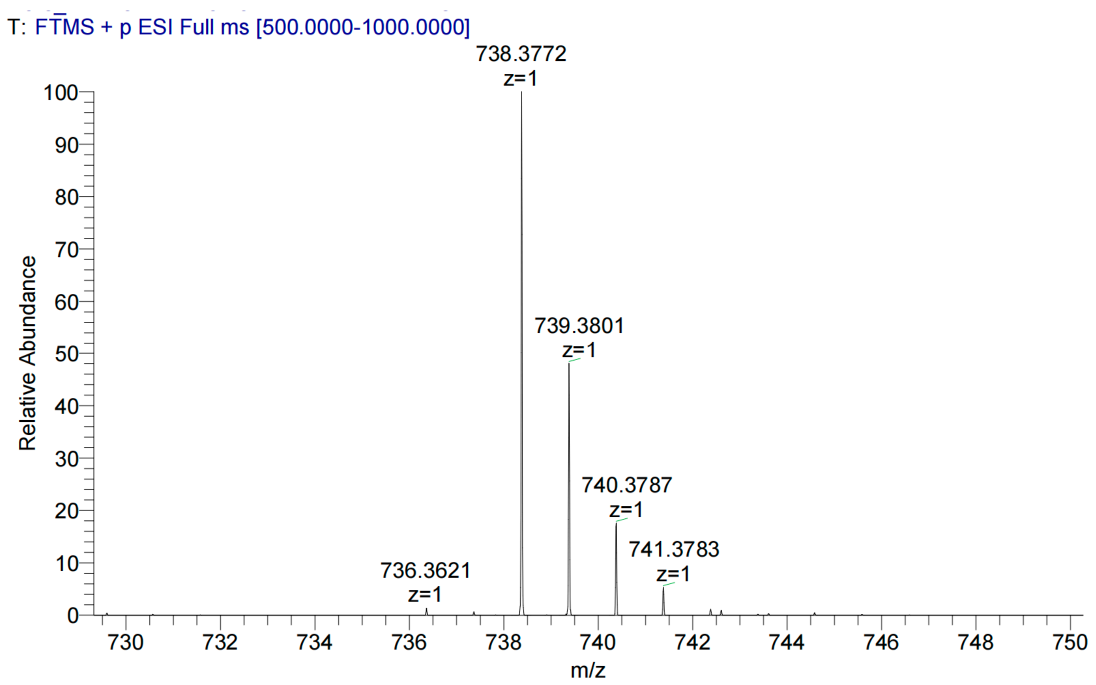

Figure S5-3 HRMS spectrum of compound **A5.**

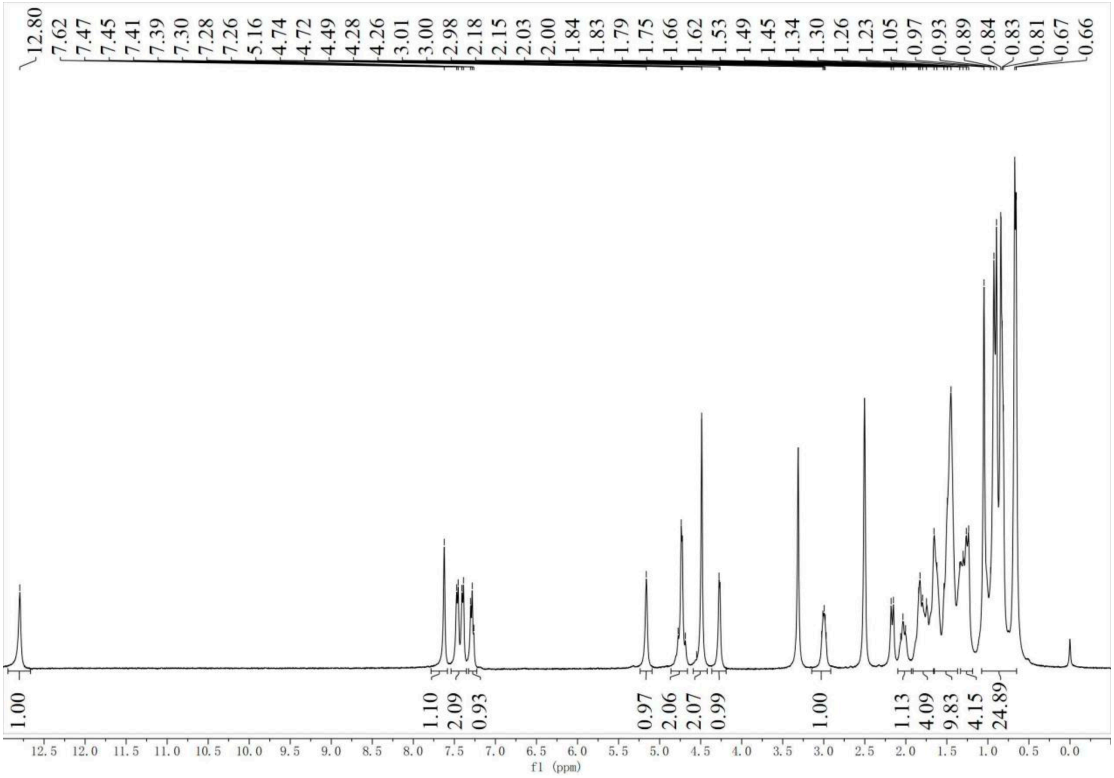

Figure S6-1 ^{1}H NMR spectrum of compound **A6.**

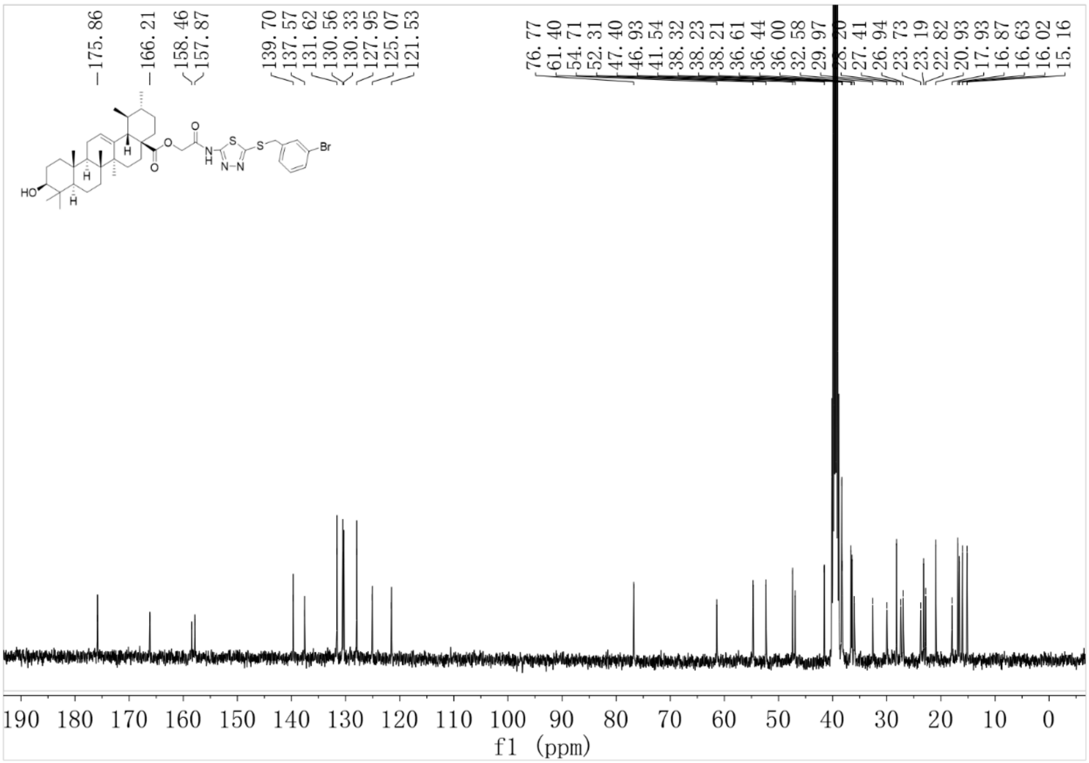

Figure S6-2 ^{13}C NMR spectrum of compound **A6.**

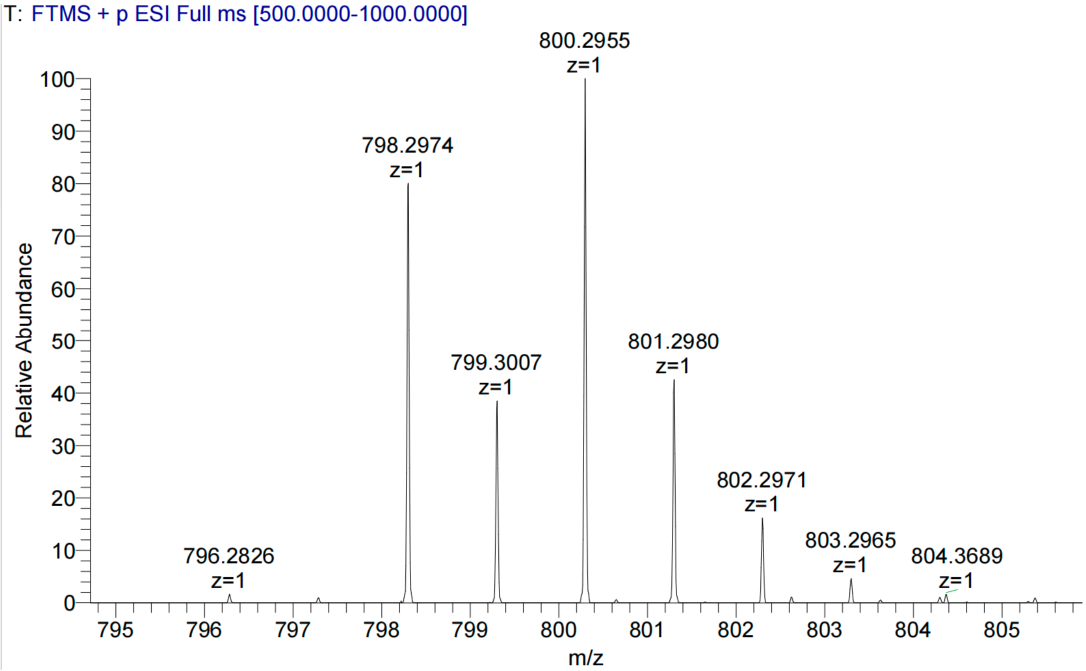

Figure S6-3 HRMS spectrum of compound **A6.**

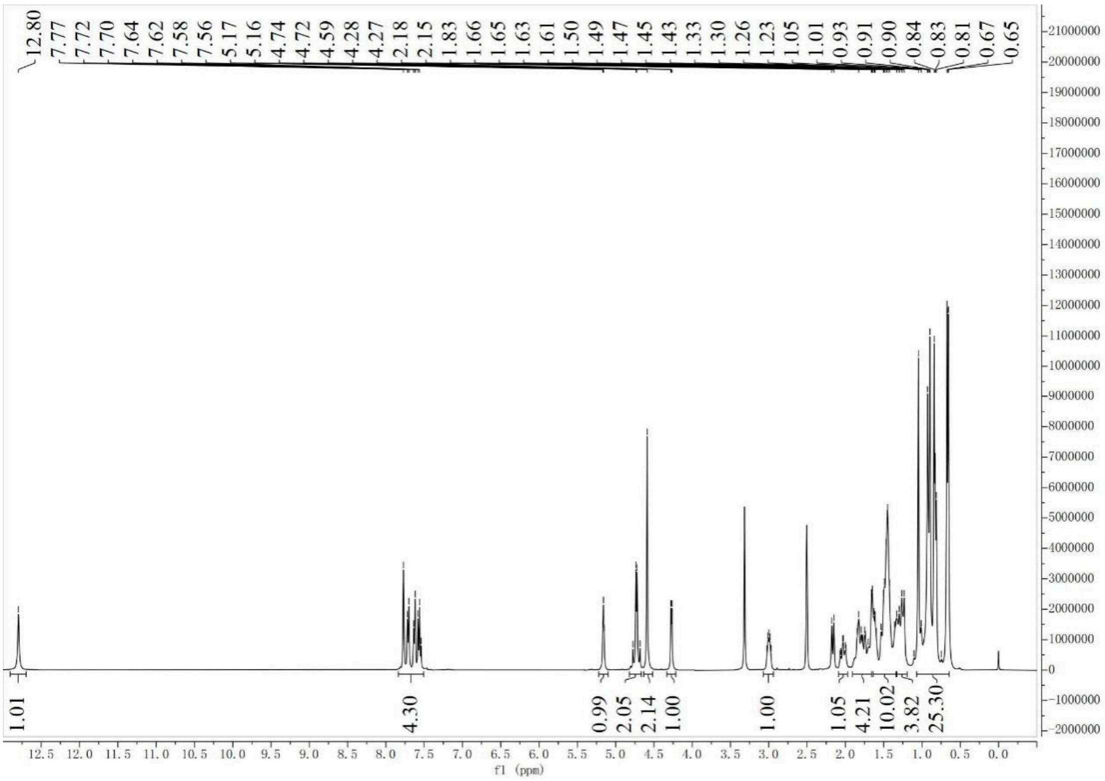

Figure S7-1 ^{1}H NMR spectrum of compound **A7.**

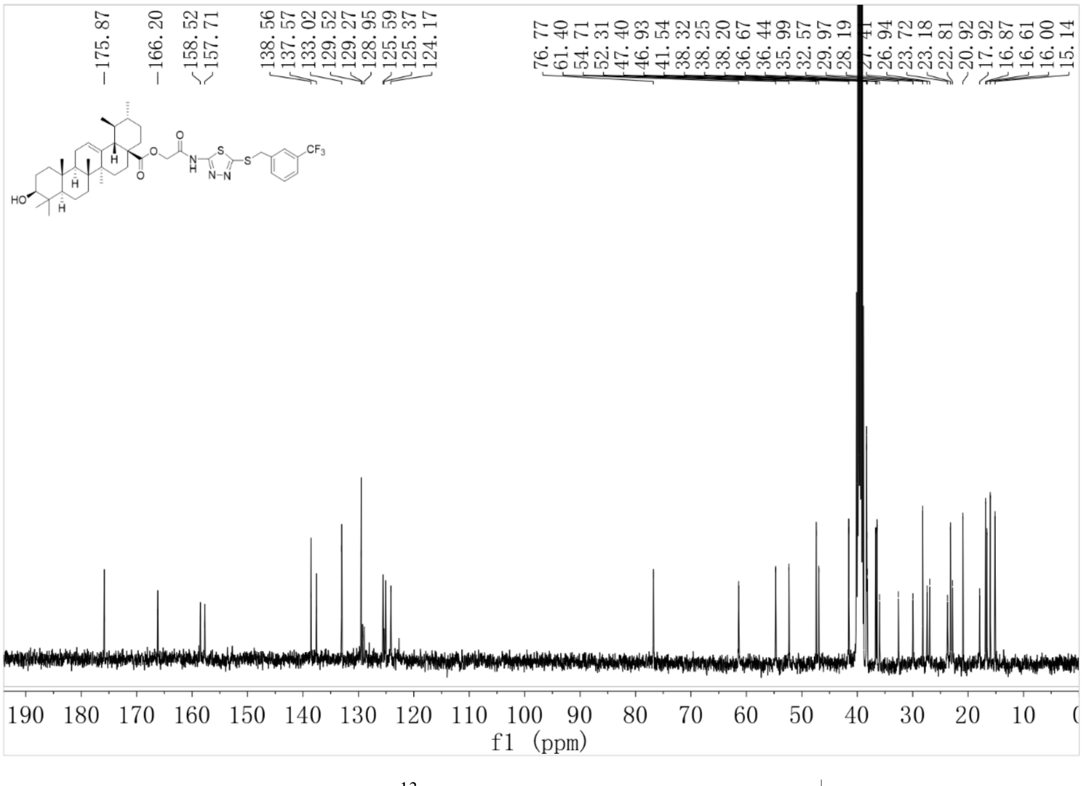

Figure S7-2 ^{13}C NMR spectrum of compound **A7.**

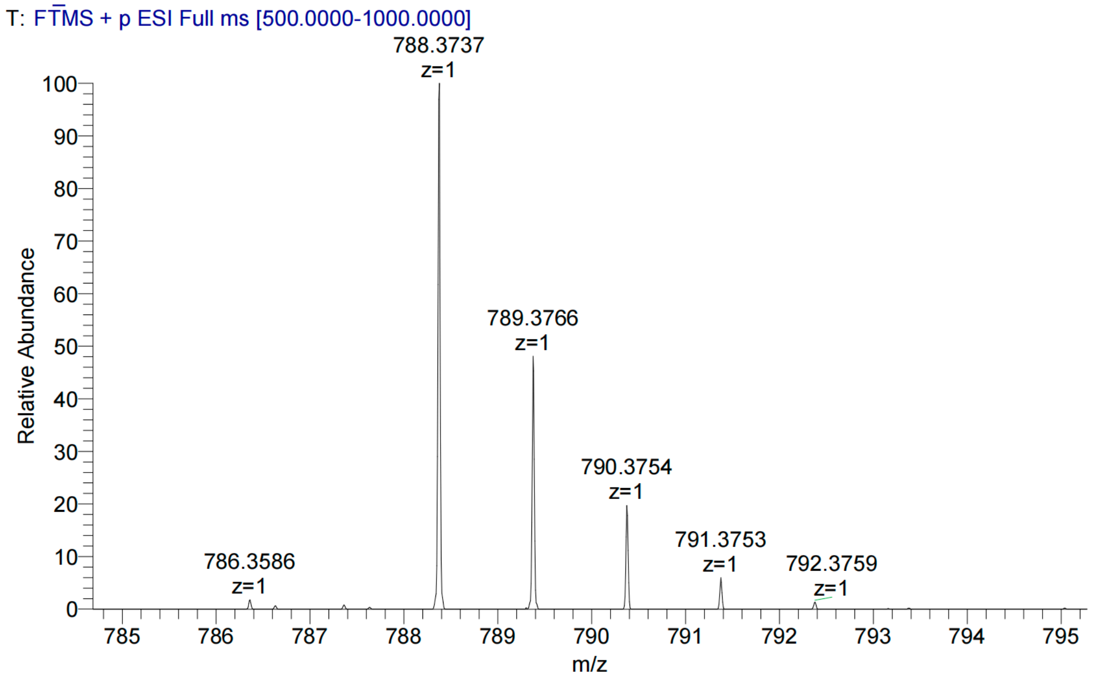

Figure S7-3 HRMS spectrum of compound **A7.**

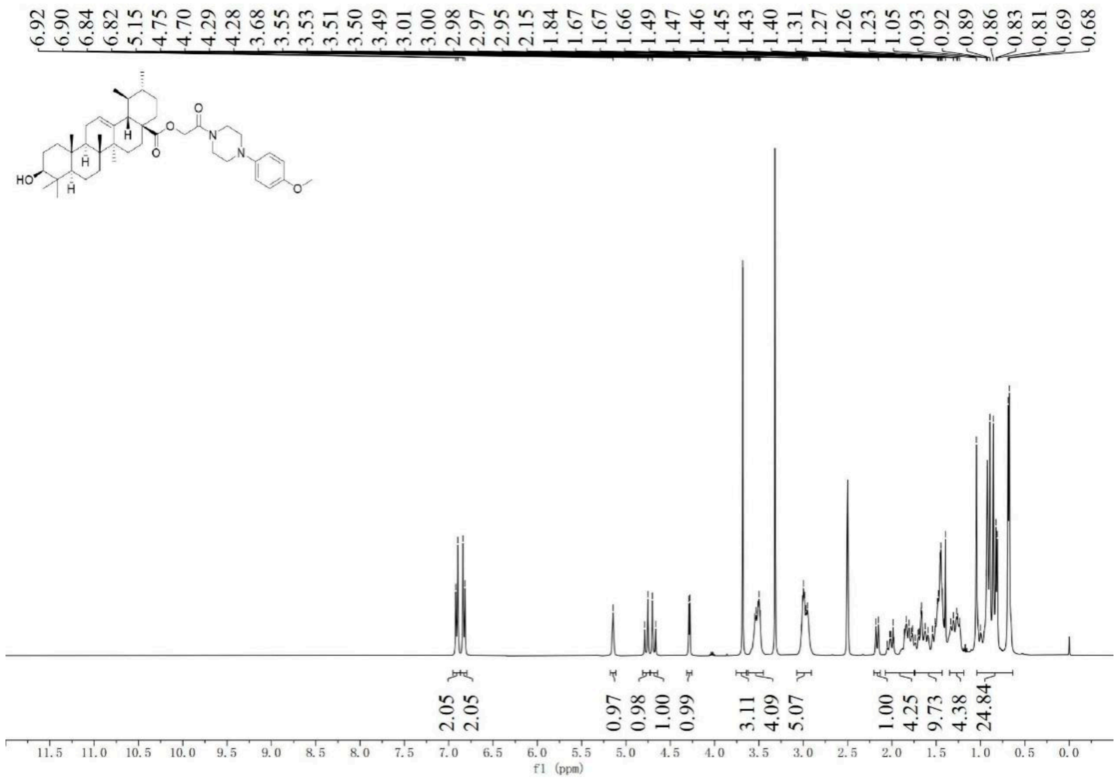

Figure S8-1 ^{1}H NMR spectrum of compound **B1.**

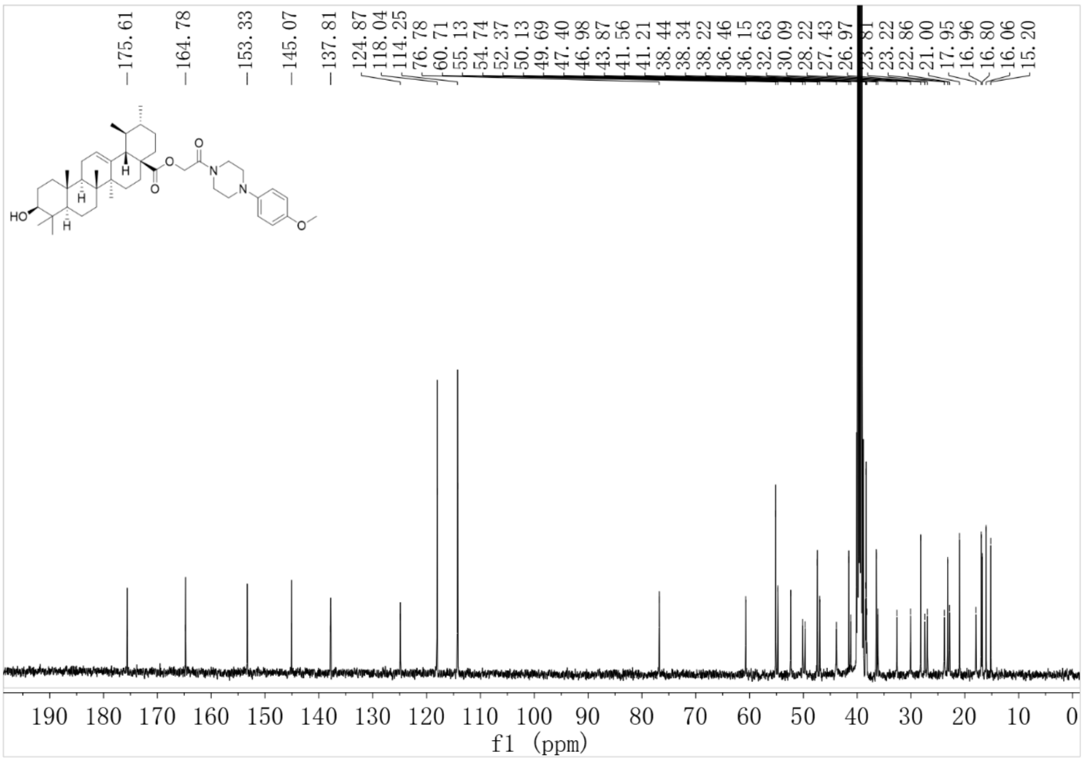

Figure S8-2 ^{13}C NMR spectrum of compound **B1.**

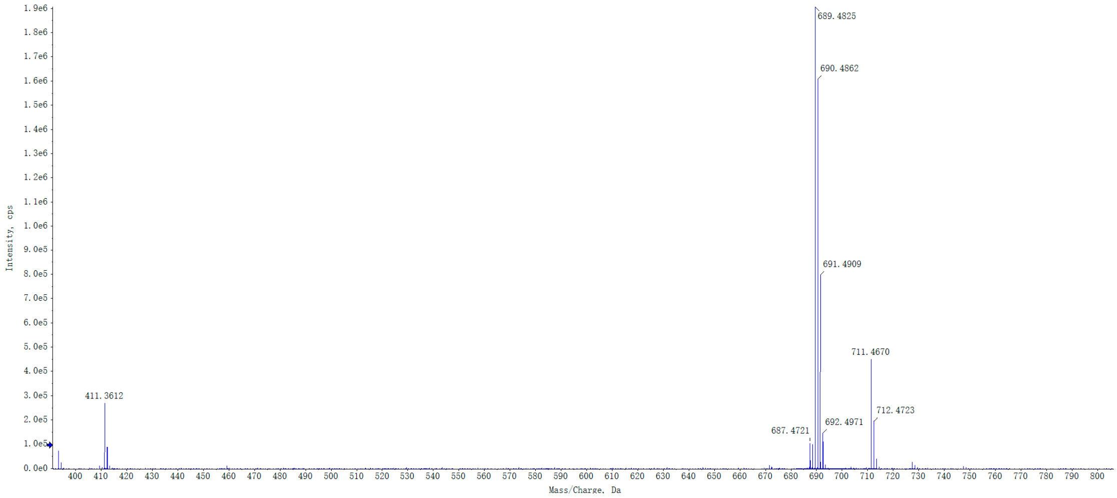

Figure S8-3 HRMS spectrum of compound **B1.**

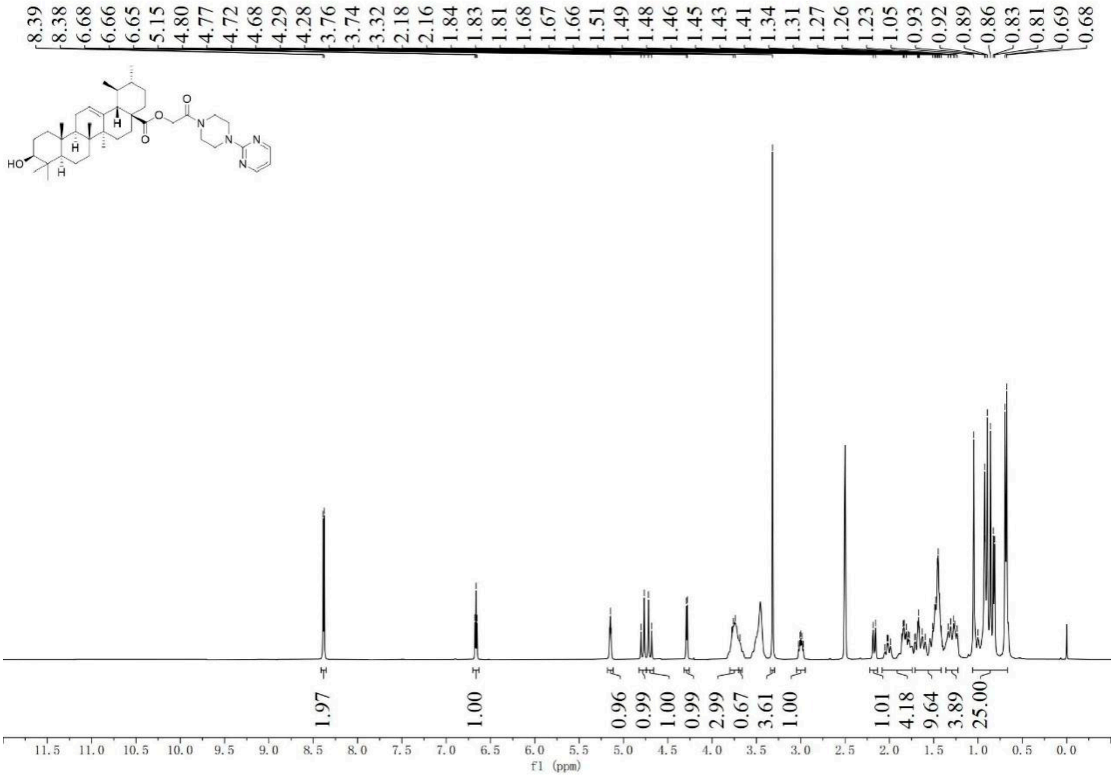

Figure S9-1 ^{1}H NMR spectrum of compound **B2.**

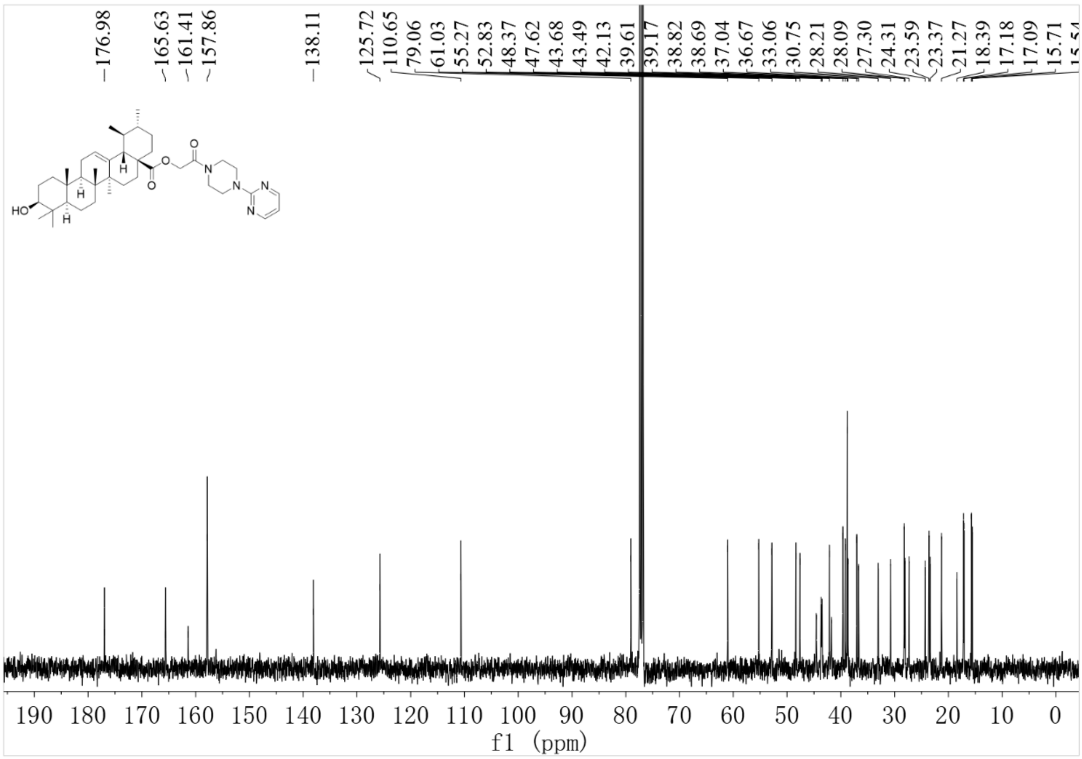

Figure S9-2 ^{13}C NMR spectrum of compound **B2.**

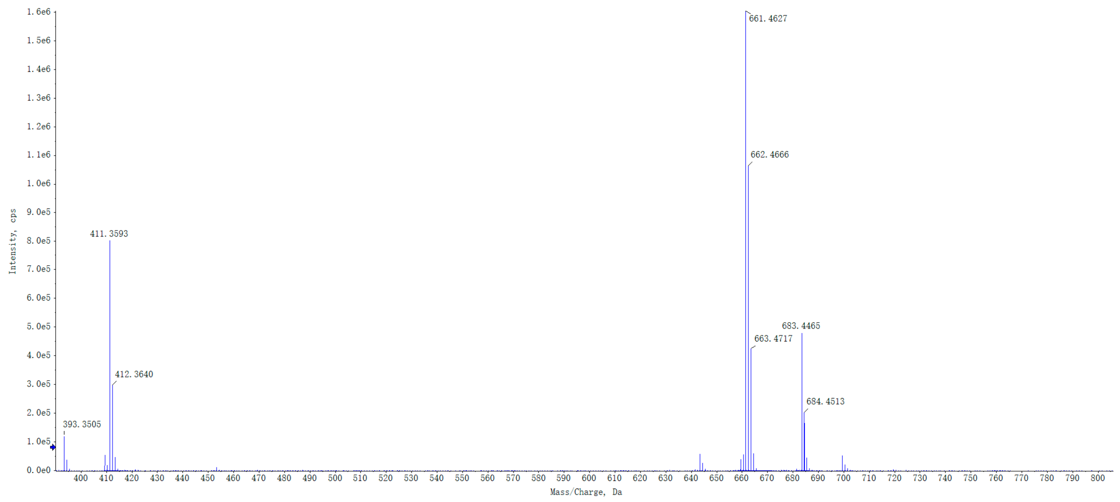

Figure S9-3 HRMS spectrum of compound **B2.**

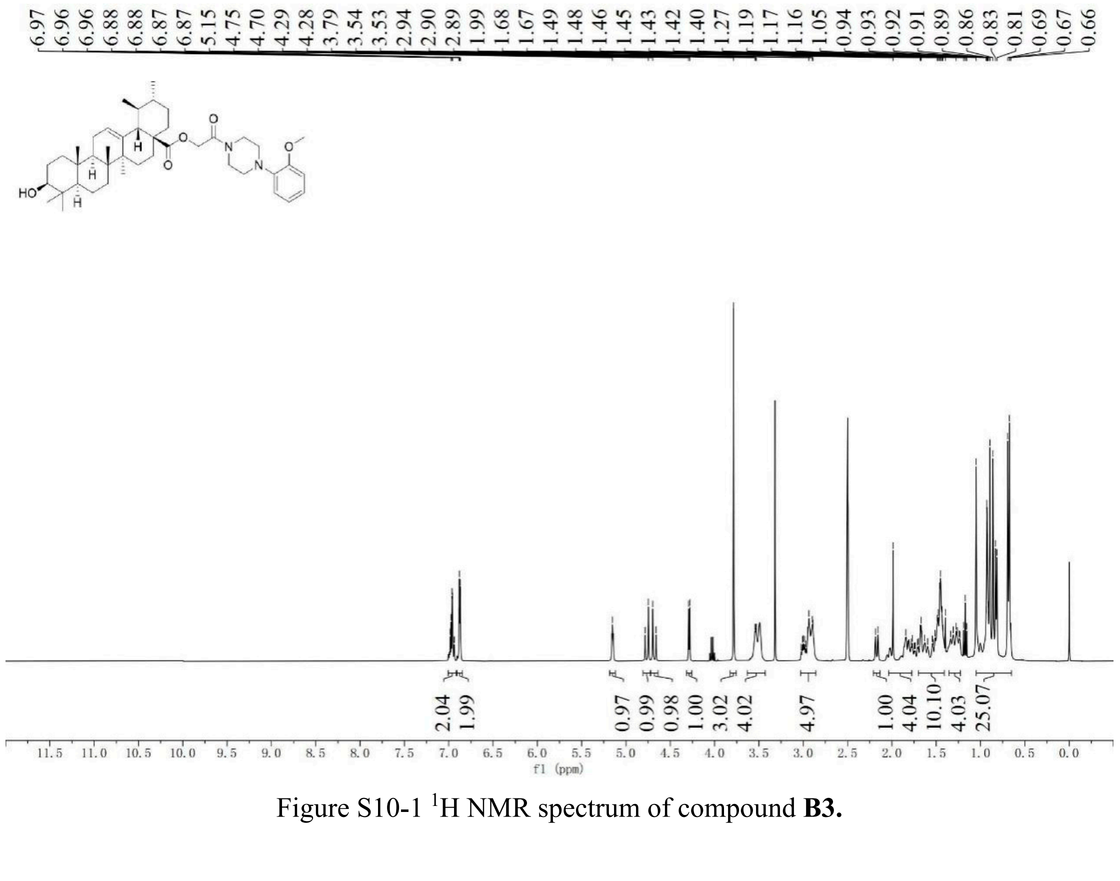

Figure S10-1 ^{1}H NMR spectrum of compound **B3.**

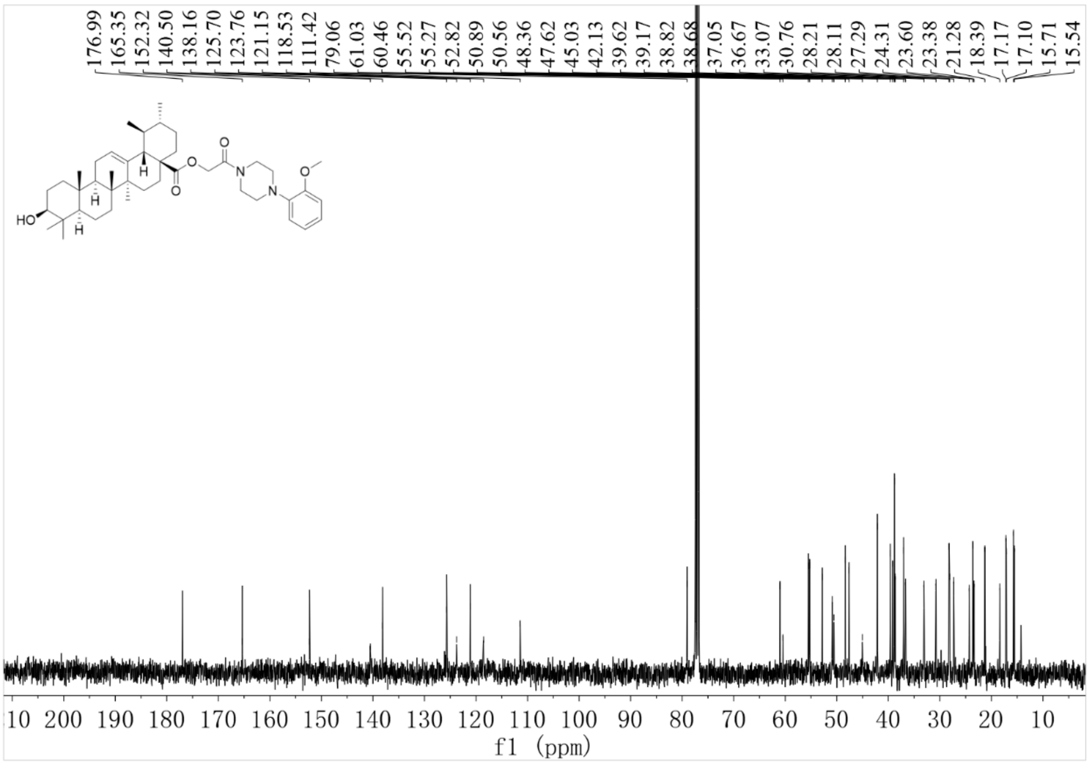

Figure S10-2 ^{13}C NMR spectrum of compound **B3.**

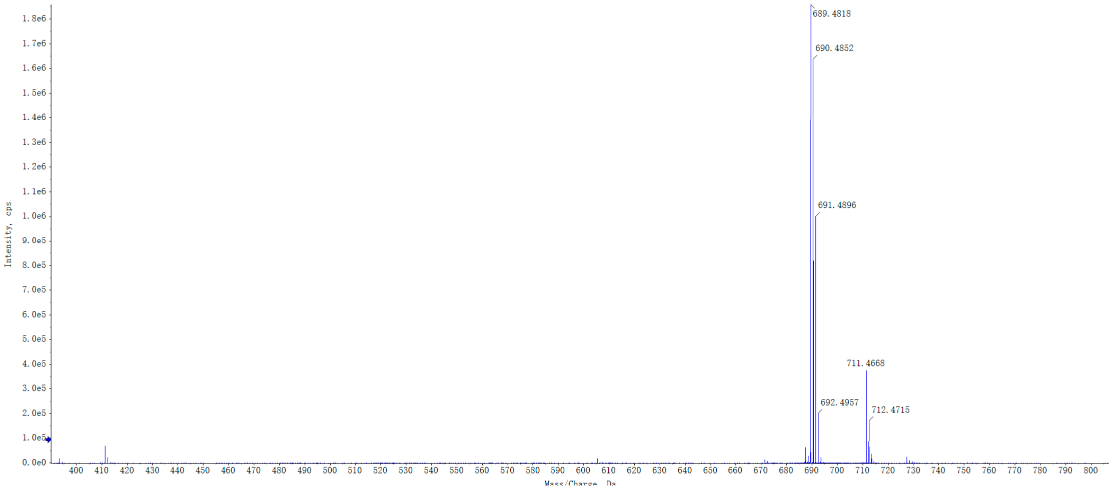

Figure S10-3 HRMS spectrum of compound **B3.**

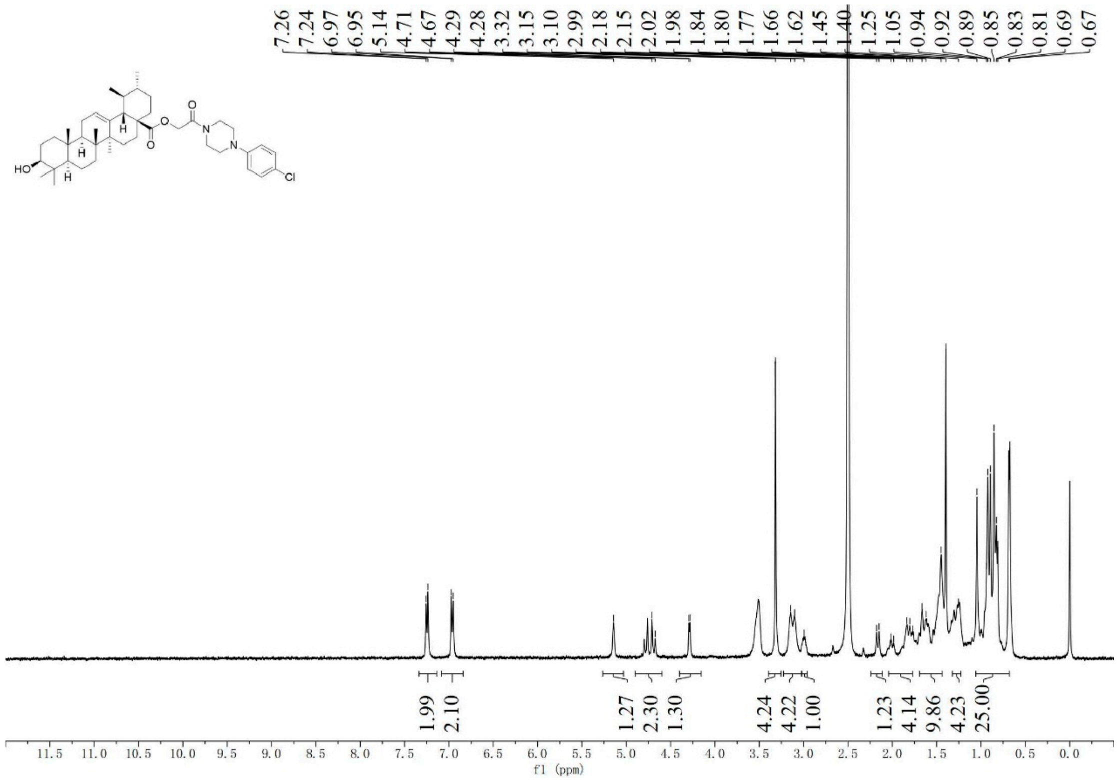

Figure S11-1 ^{1}H NMR spectrum of compound **B4.**

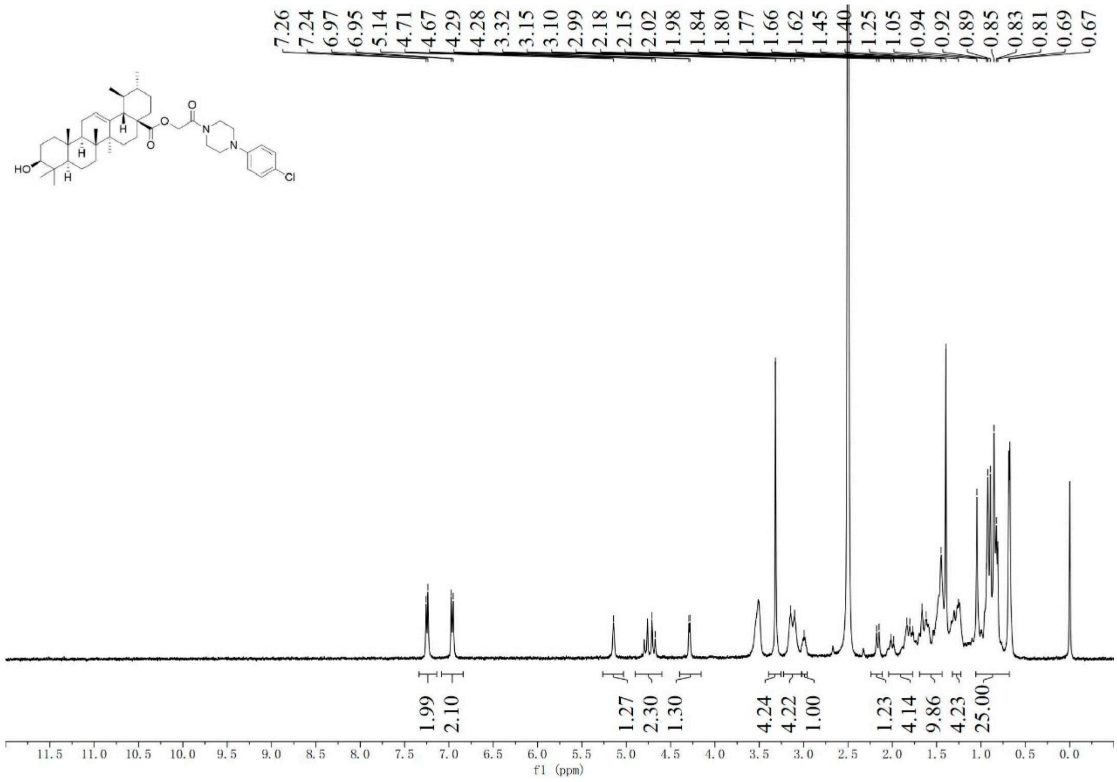

Figure S11-2 ^{13}C NMR spectrum of compound **B4.**

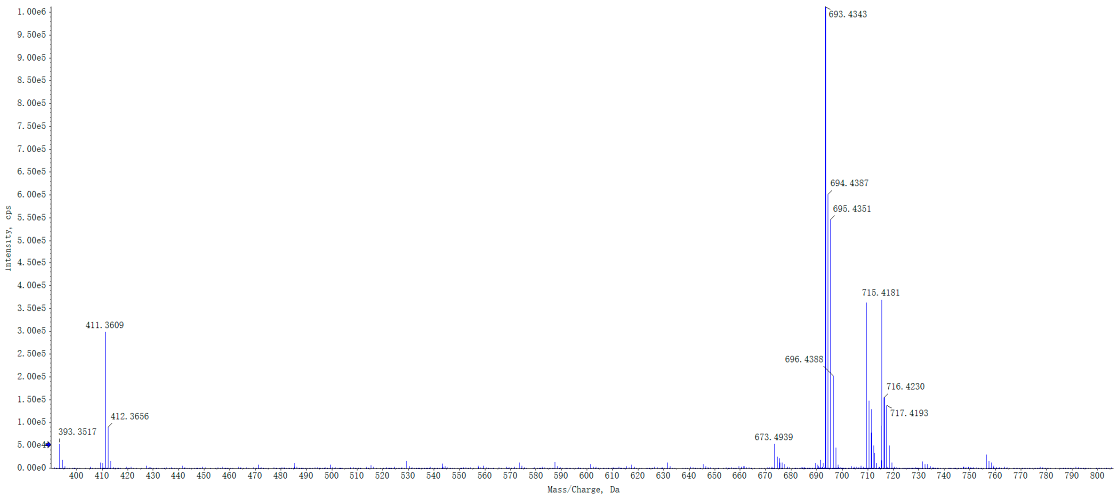

Figure S11-3 HRMS spectrum of compound **B4.**

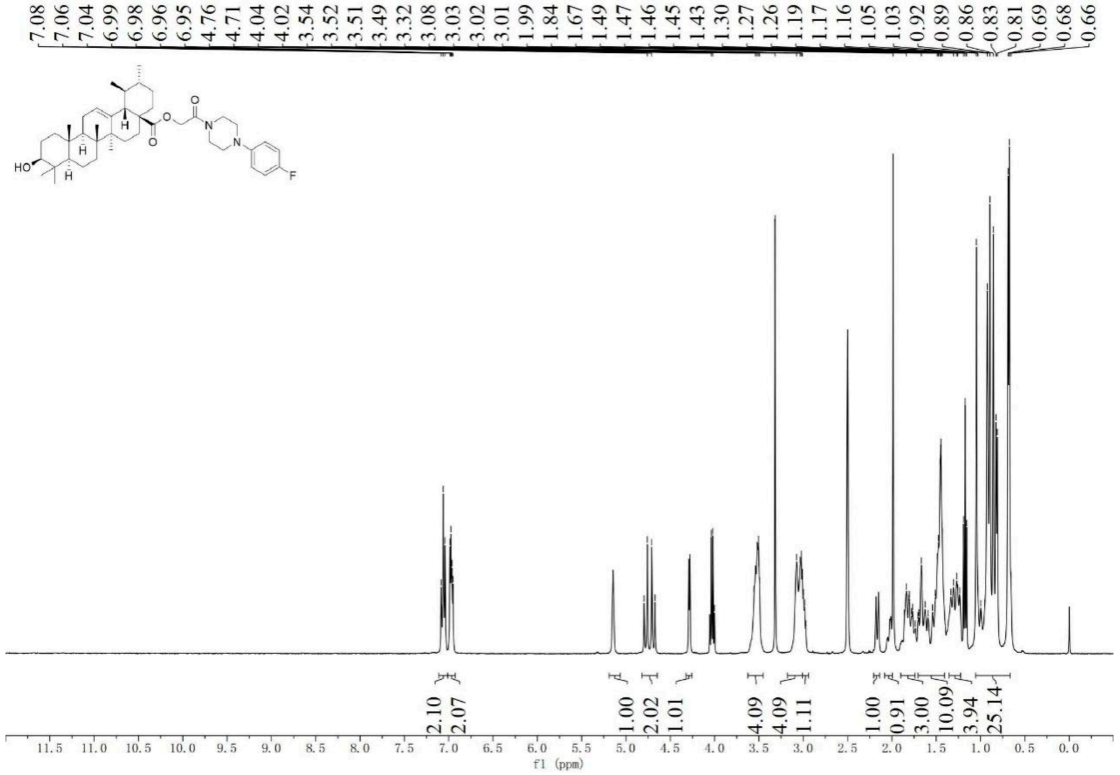

Figure S12-1 ^{1}H NMR spectrum of compound **B5.**

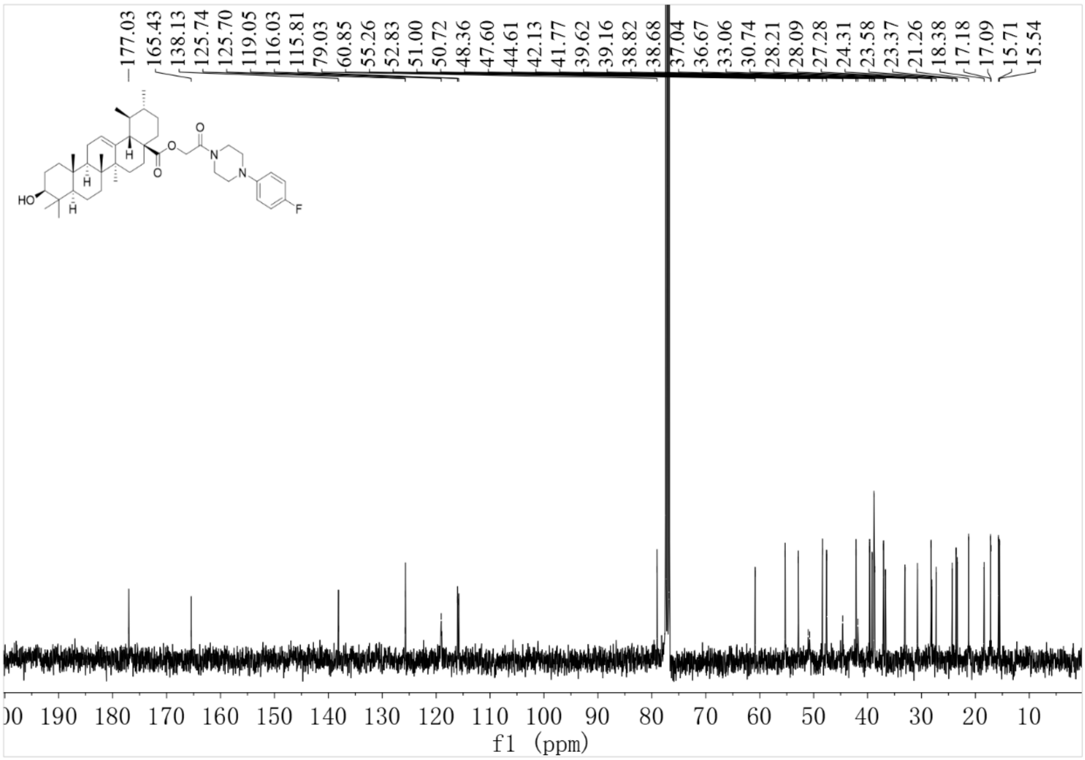

Figure S12-2 ^{13}C NMR spectrum of compound **B5.**

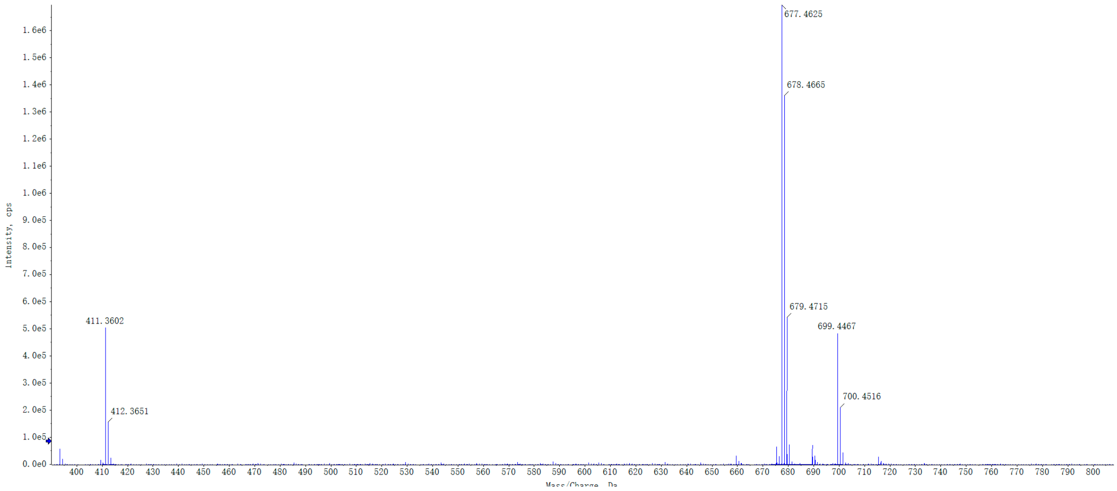

Figure S12-3 HRMS spectrum of compound **B5.**

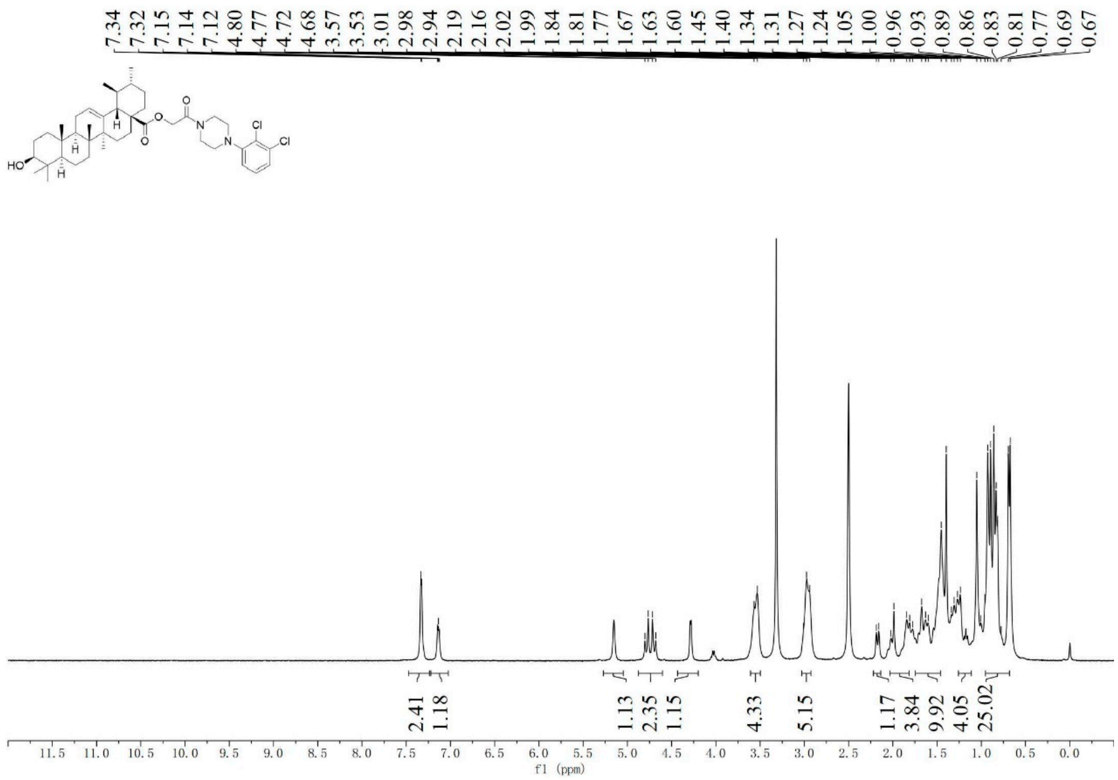

Figure S13-1 ^{1}H NMR spectrum of compound **B6.**

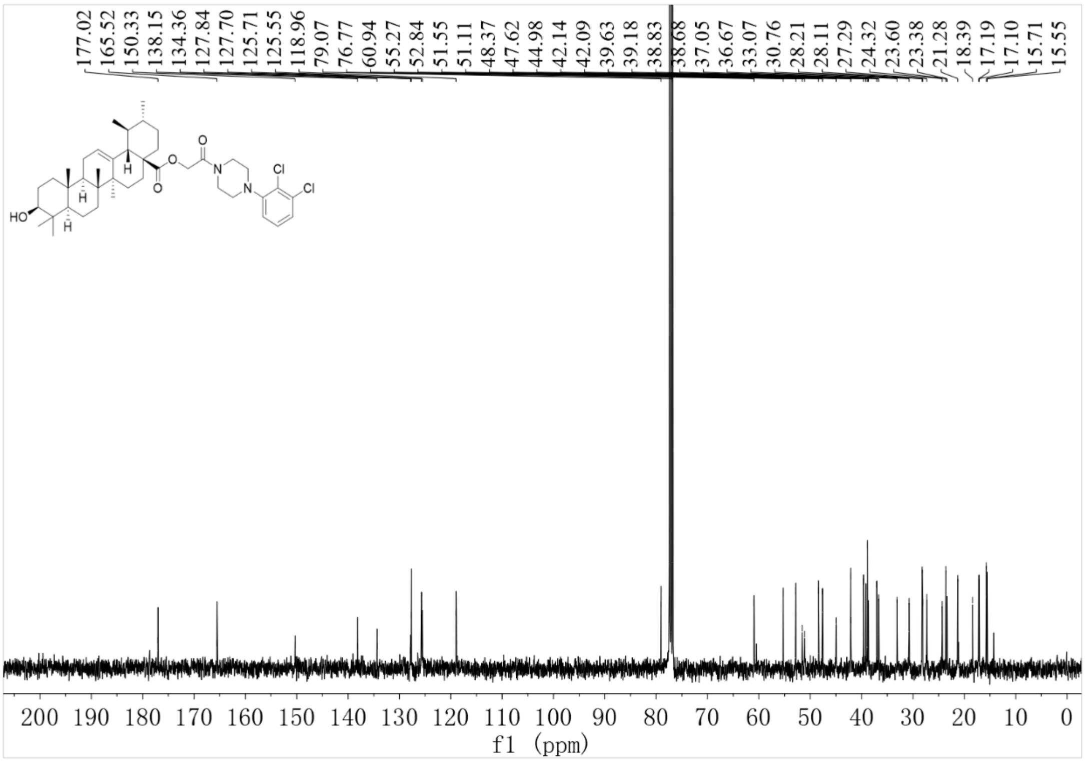

Figure S13-2 ^{13}C NMR spectrum of compound **B6.**

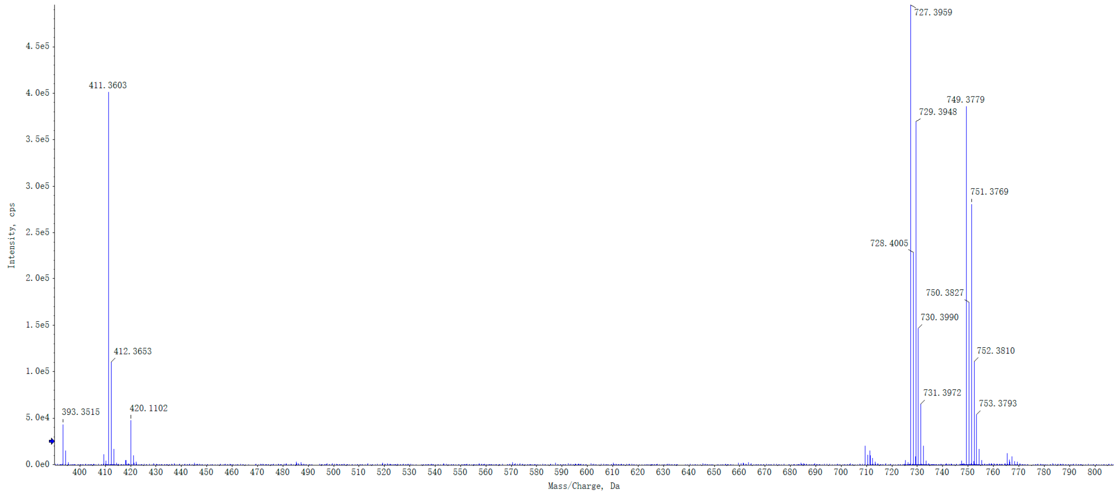

Figure S13-3 HRMS spectrum of compound **B6.**

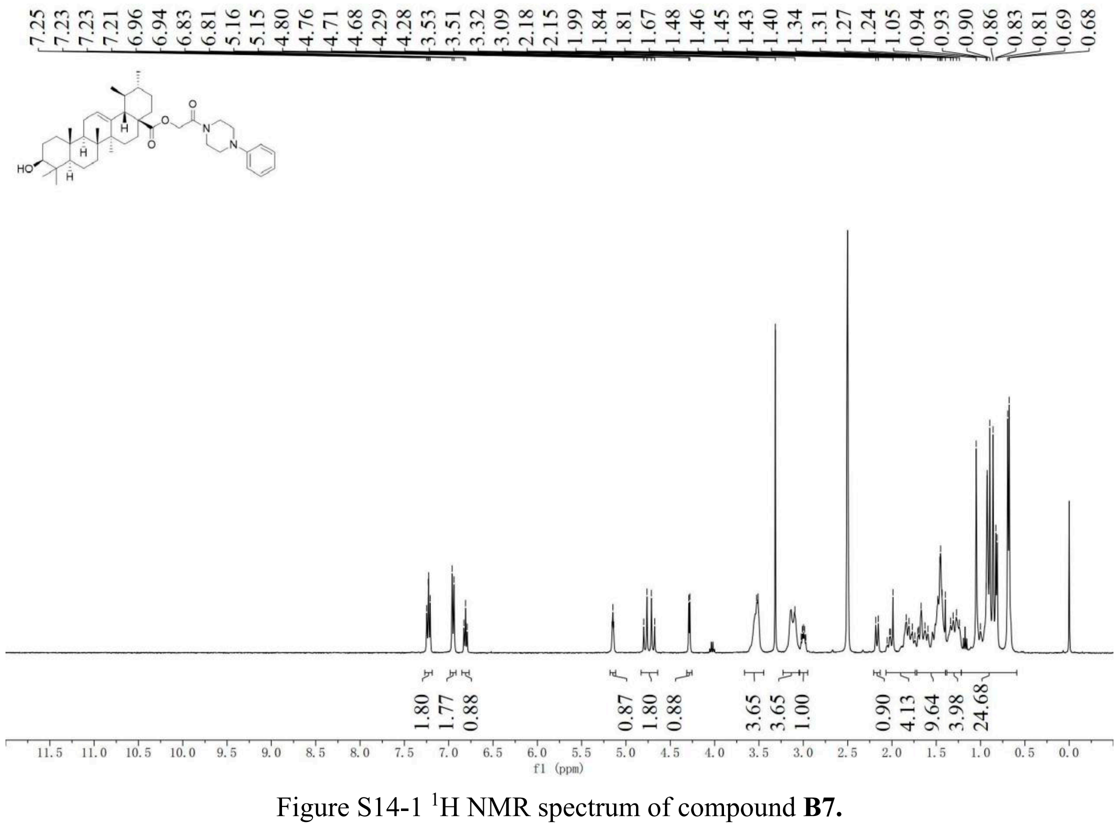

Figure S14-1 ^{1}H NMR spectrum of compound **B7.**

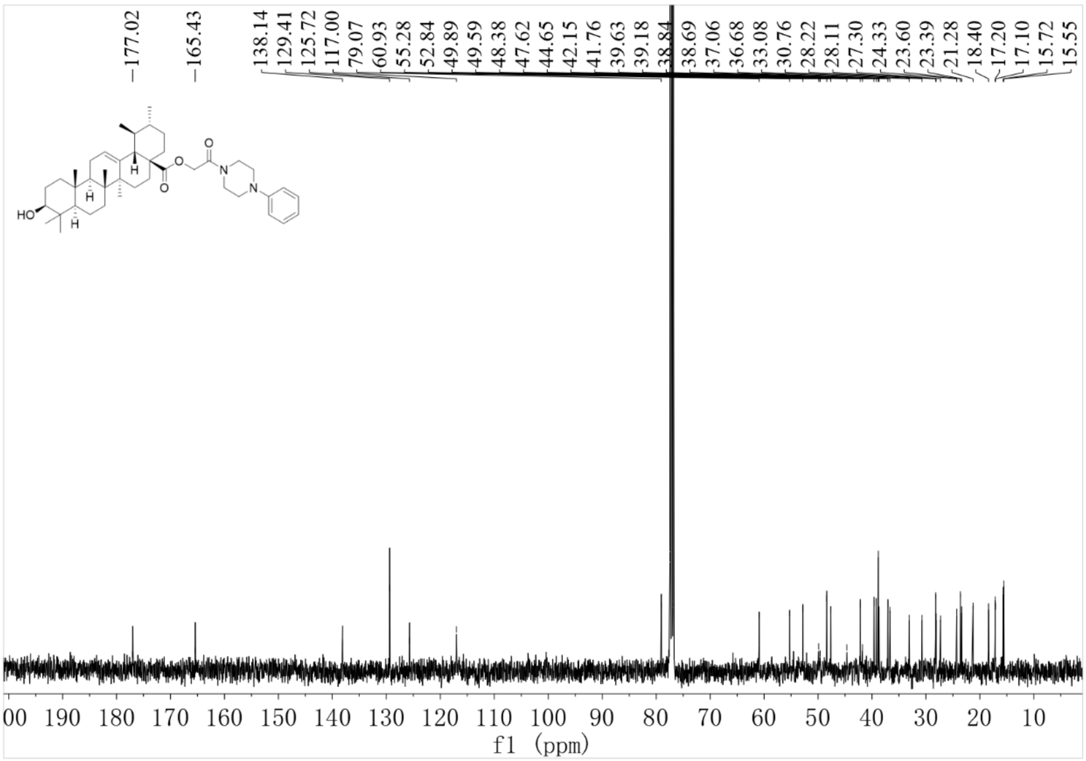

Figure S14-2 ^{13}C NMR spectrum of compound **B7.**

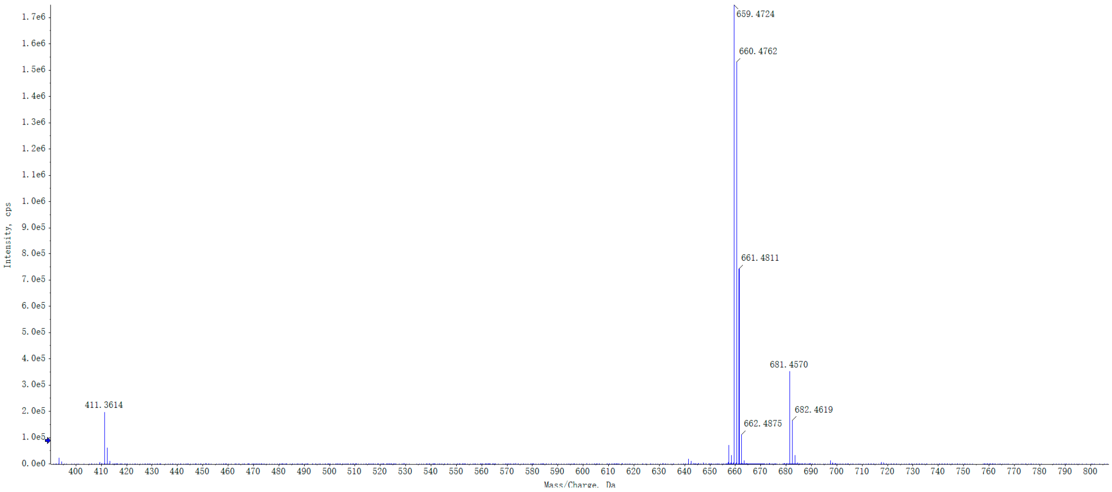

Figure S14-3 HRMS spectrum of compound **B7.**

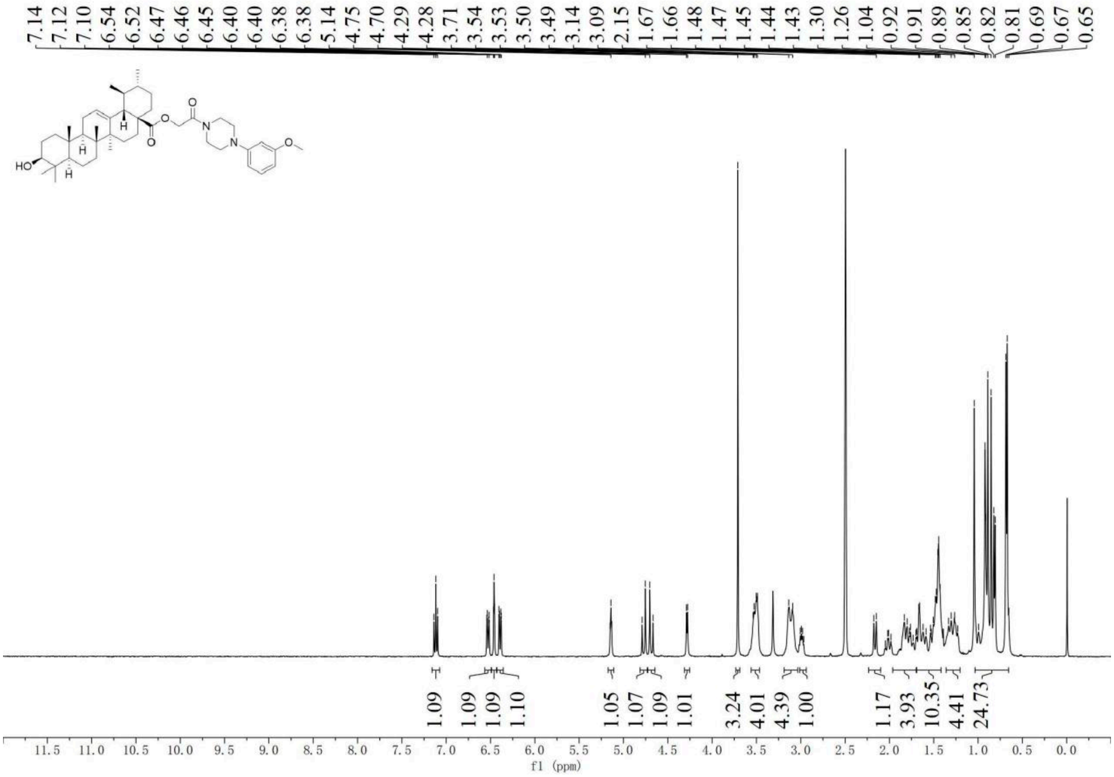

Figure S15-1 ^{1}H NMR spectrum of compound **B8.**

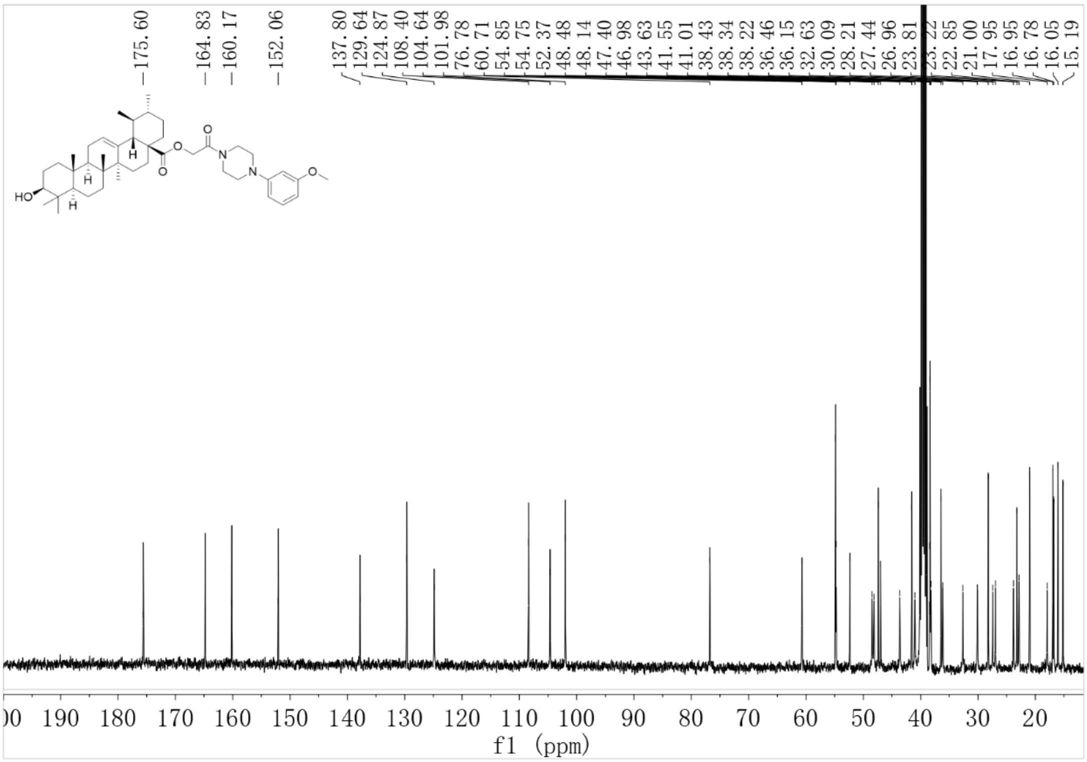

Figure S15-2 ^{13}C NMR spectrum of compound **B8.**

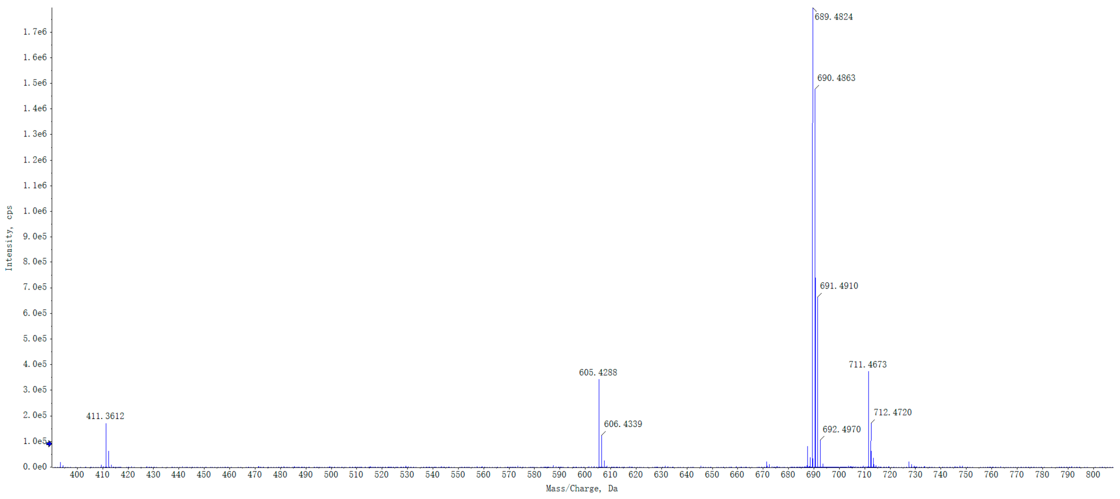

Figure S15-3 HRMS spectrum of compound **B8.**

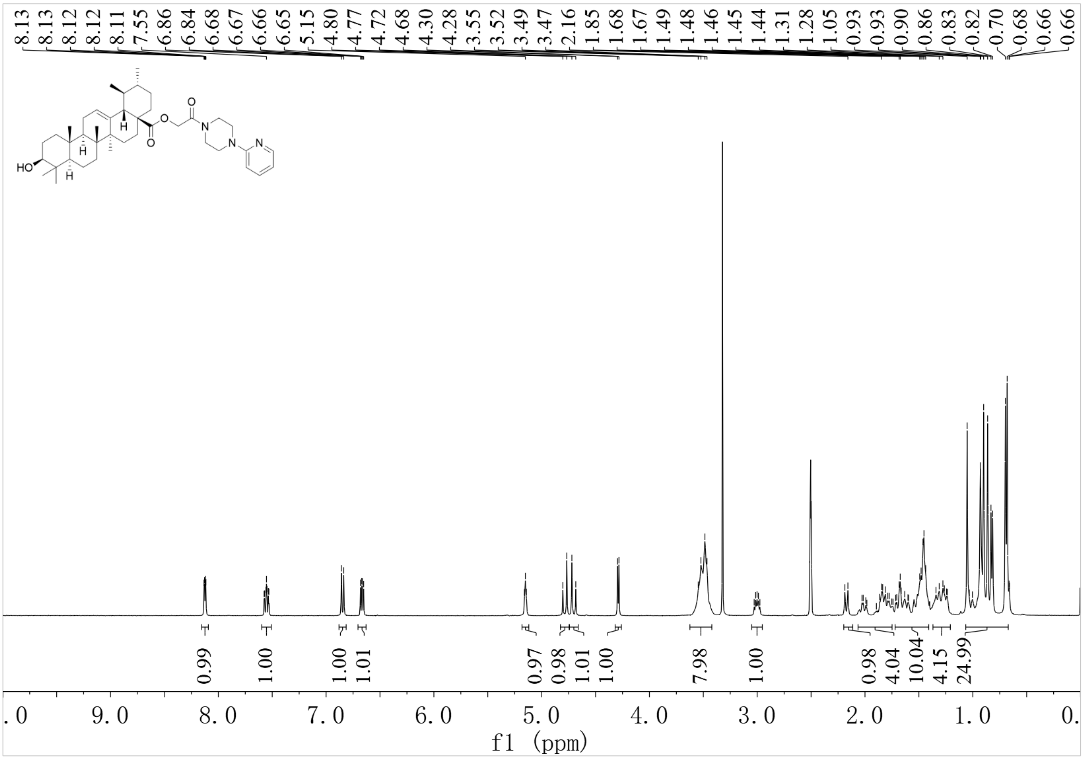

Figure S16-1 ^{1}H NMR spectrum of compound **B9.**

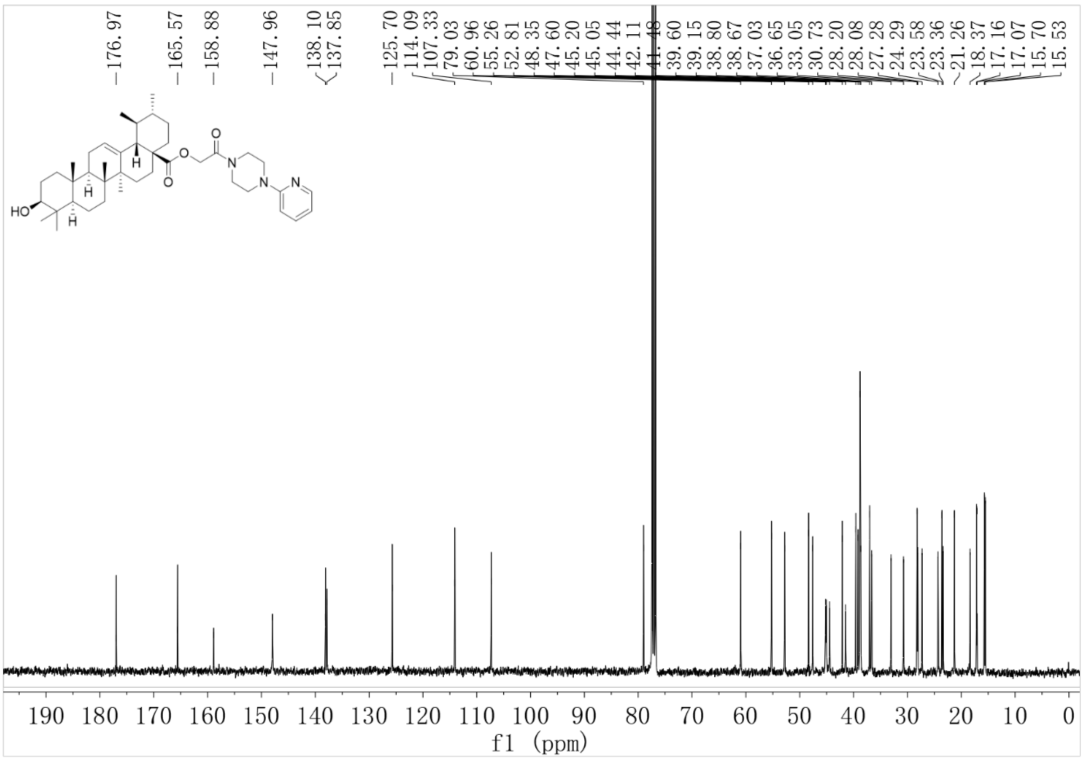

Figure S16-2 ^{13}C NMR spectrum of compound **B9.**

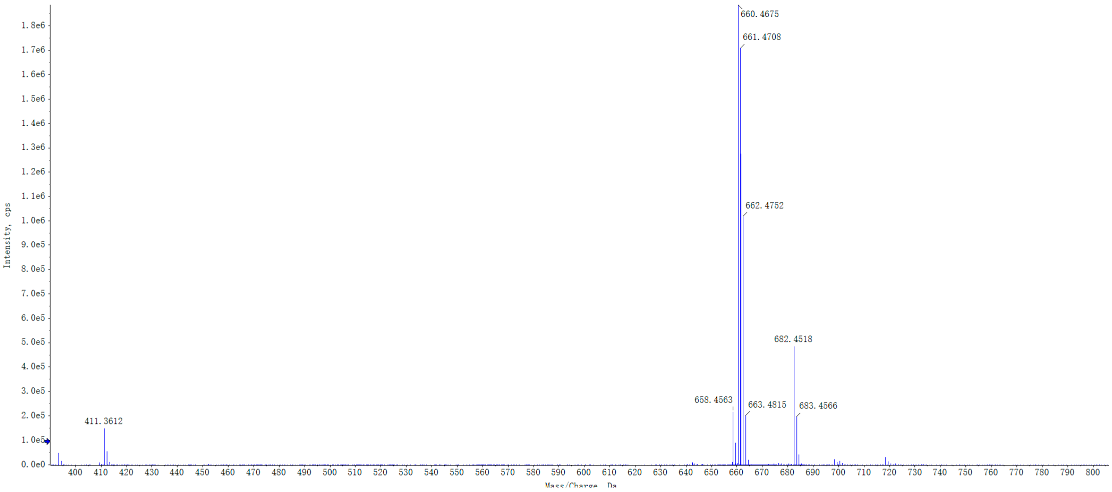

Figure S16-3 HRMS spectrum of compound **B9.**

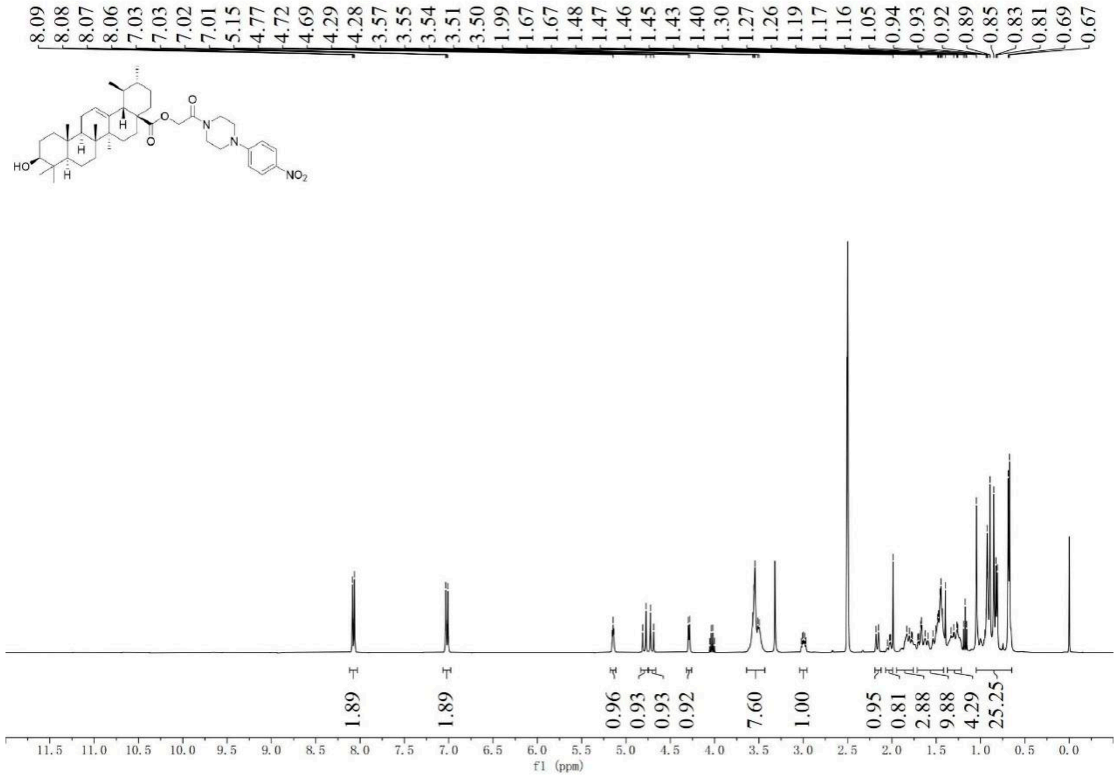

Figure S17-1 ^{1}H NMR spectrum of compound **B10.**

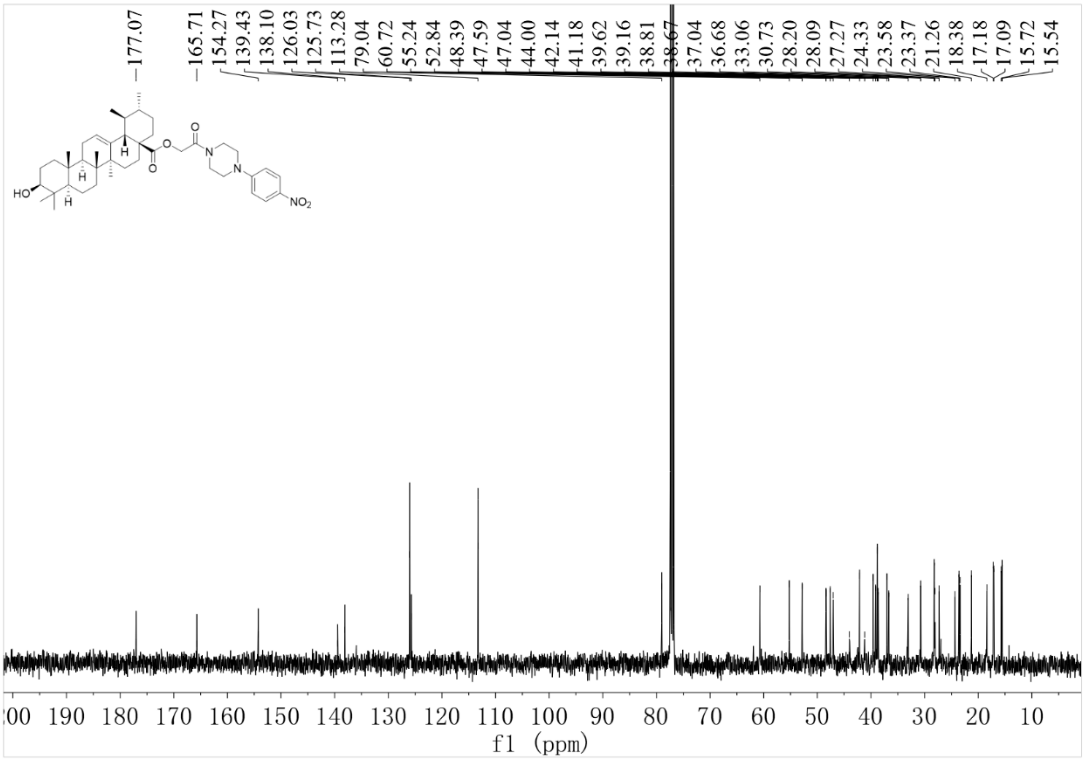

Figure S17-2 ^{13}C NMR spectrum of compound **B10.**

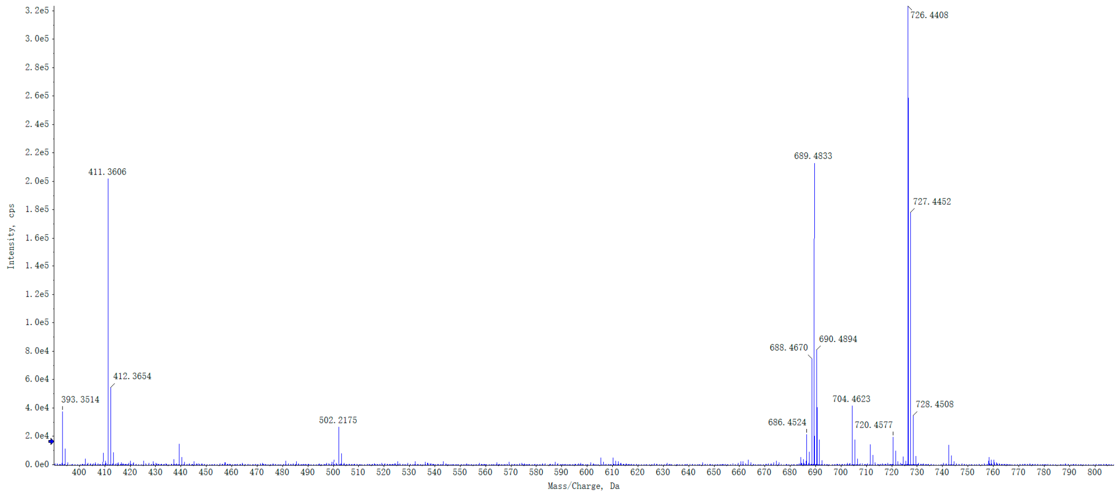

Figure S17-3 HRMS spectrum of compound **B10.**

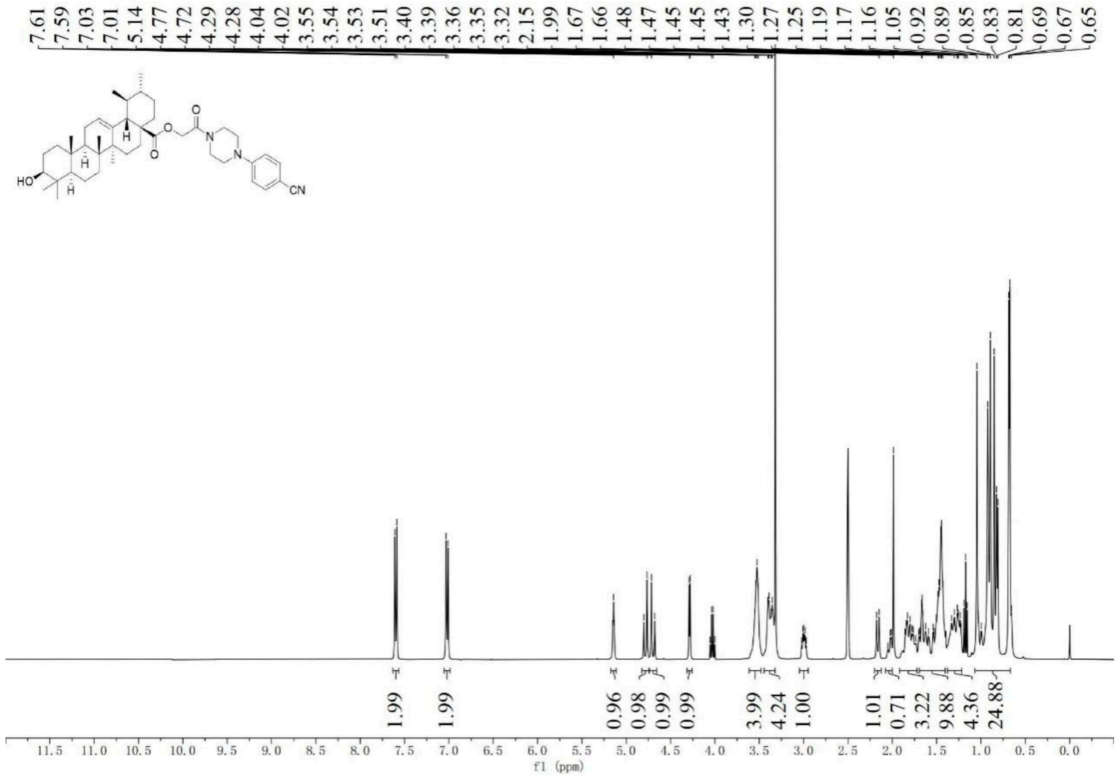

Figure S18-1 ^{1}H NMR spectrum of compound **B11.**

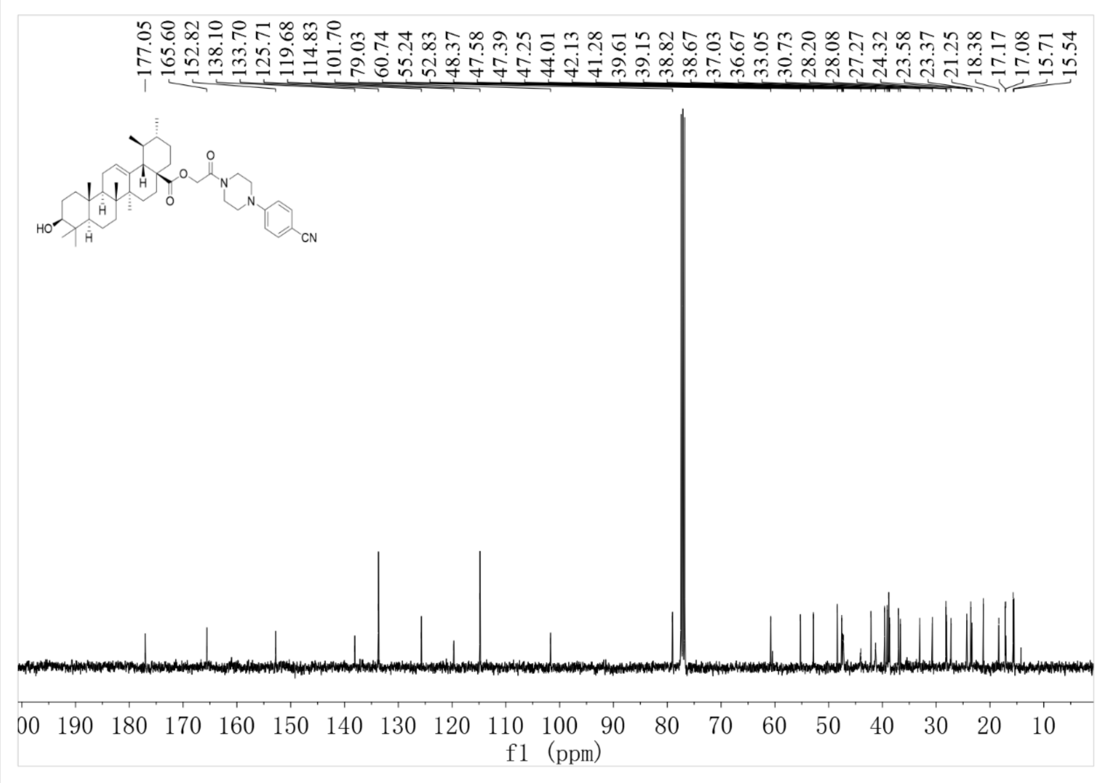

Figure S18-2 ^{13}C NMR spectrum of compound **B11.**

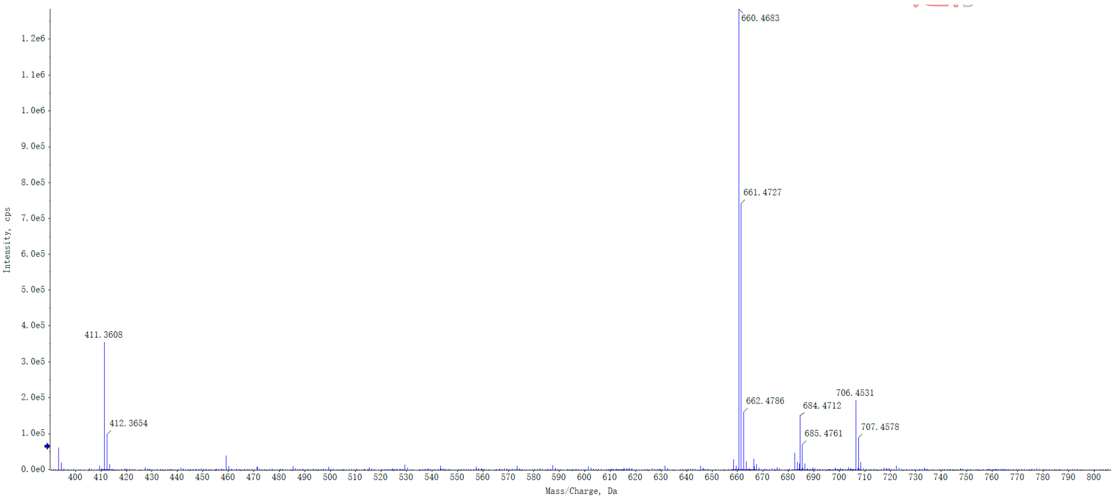

Figure S18-3 HRMS spectrum of compound **B11.**

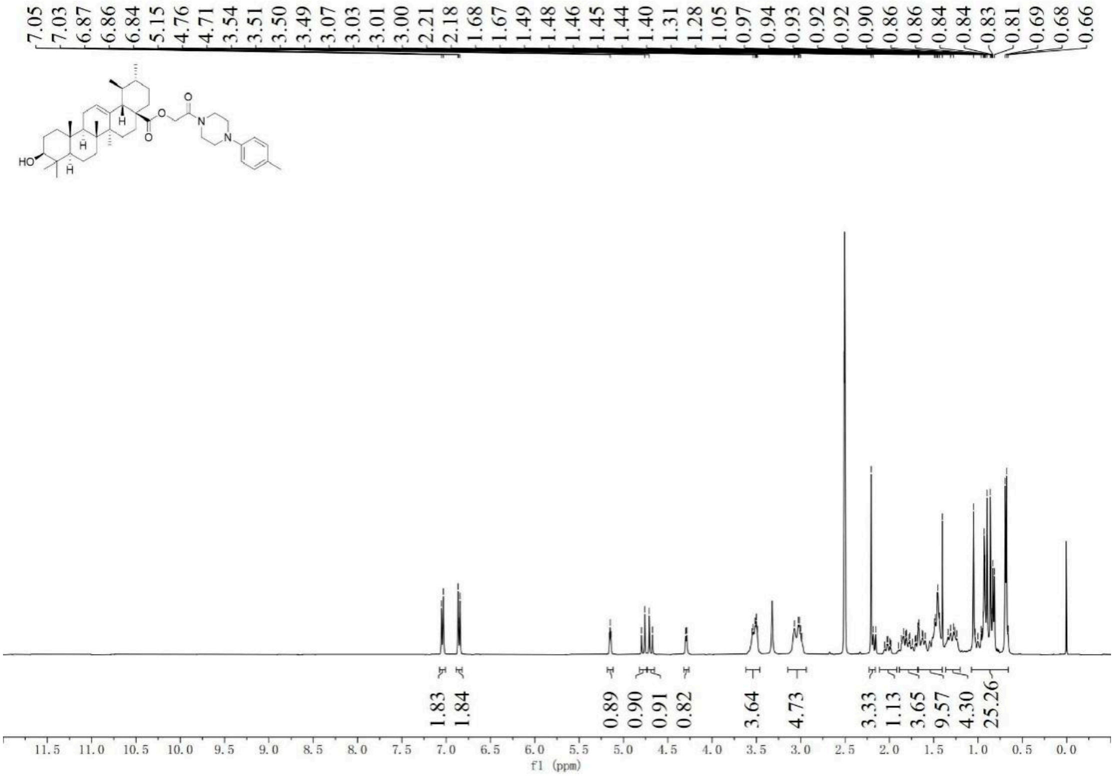

Figure S19-1 ^{1}H NMR spectrum of compound **B12.**

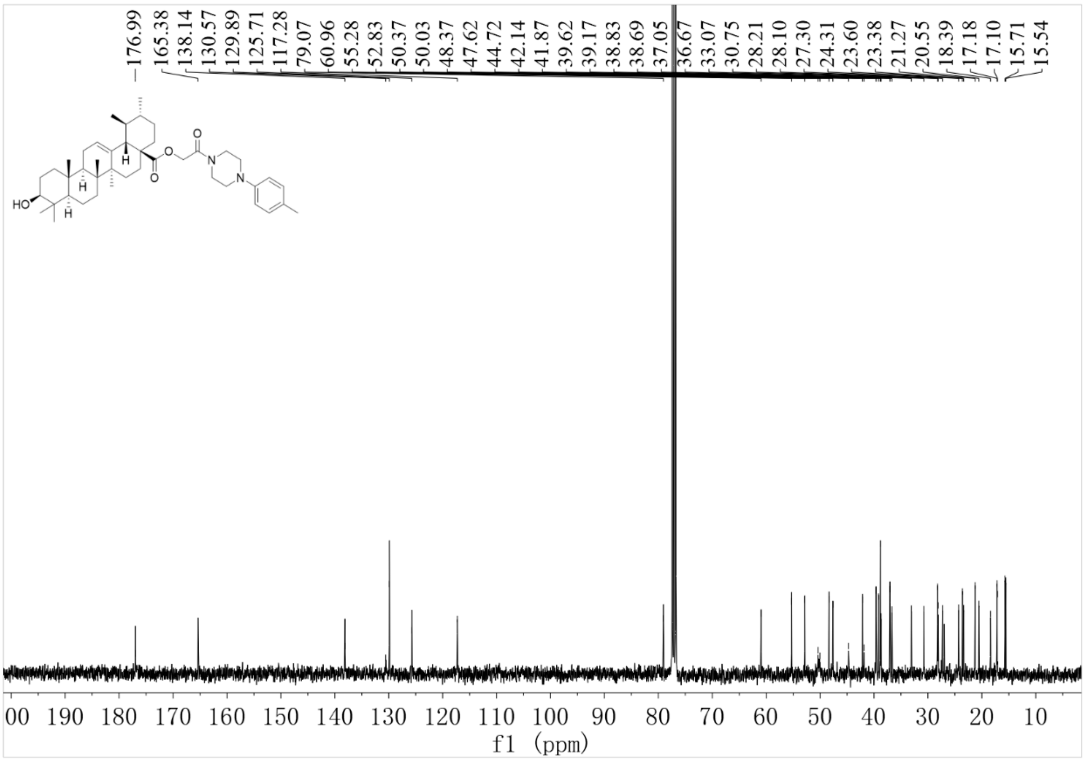

Figure S19-2 ^{13}C NMR spectrum of compound **B12.**

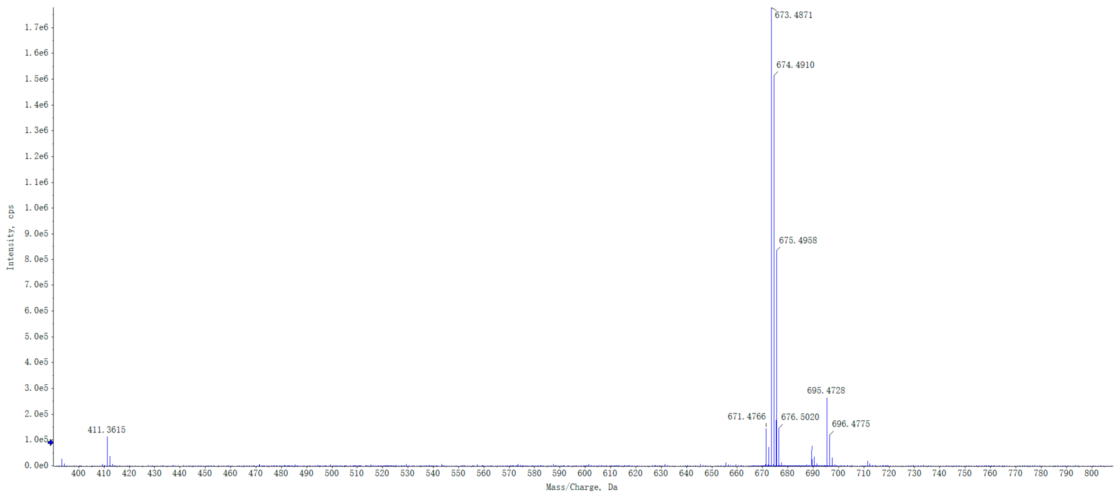

Figure S19-3 HRMS spectrum of compound **B12.**

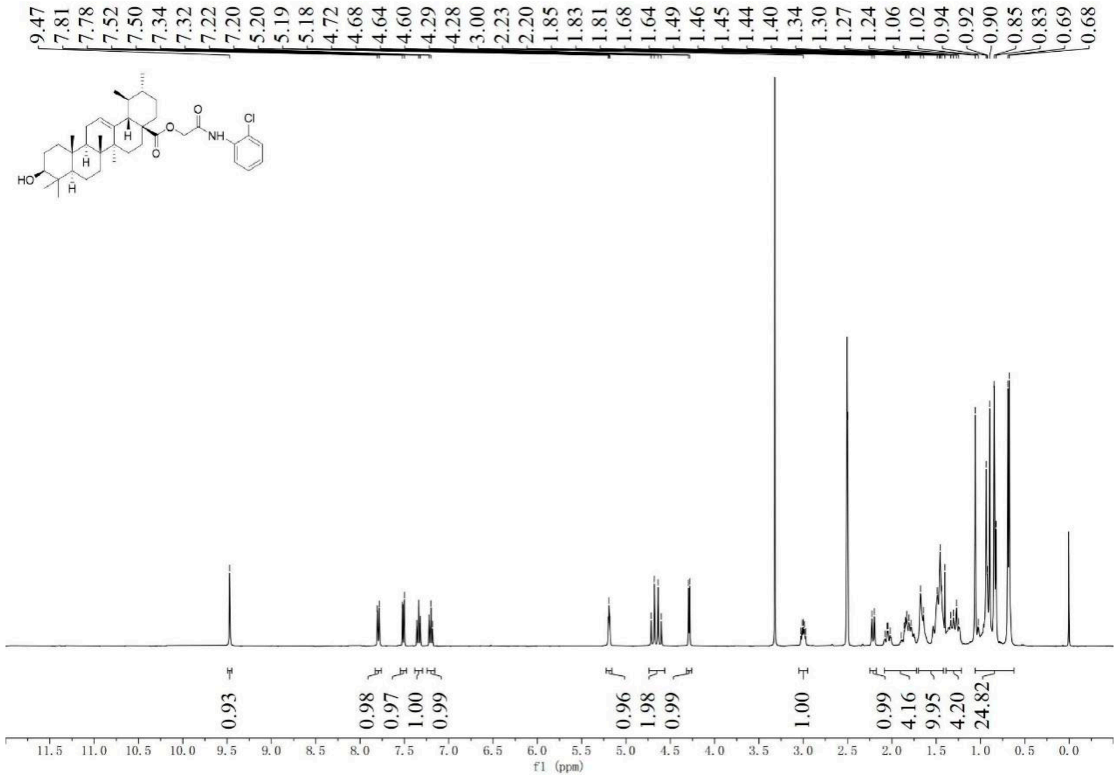

Figure S20-1 ^{1}H NMR spectrum of compound **B13.**

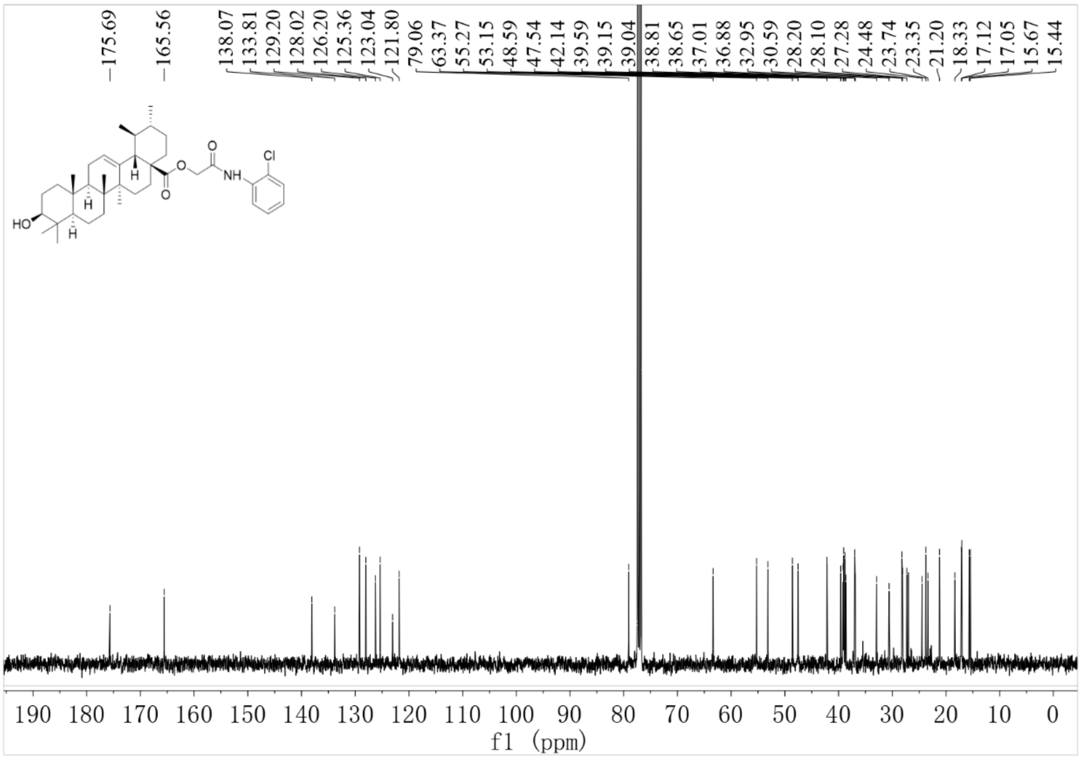

Figure S20-2 ^{13}C NMR spectrum of compound **B13.**

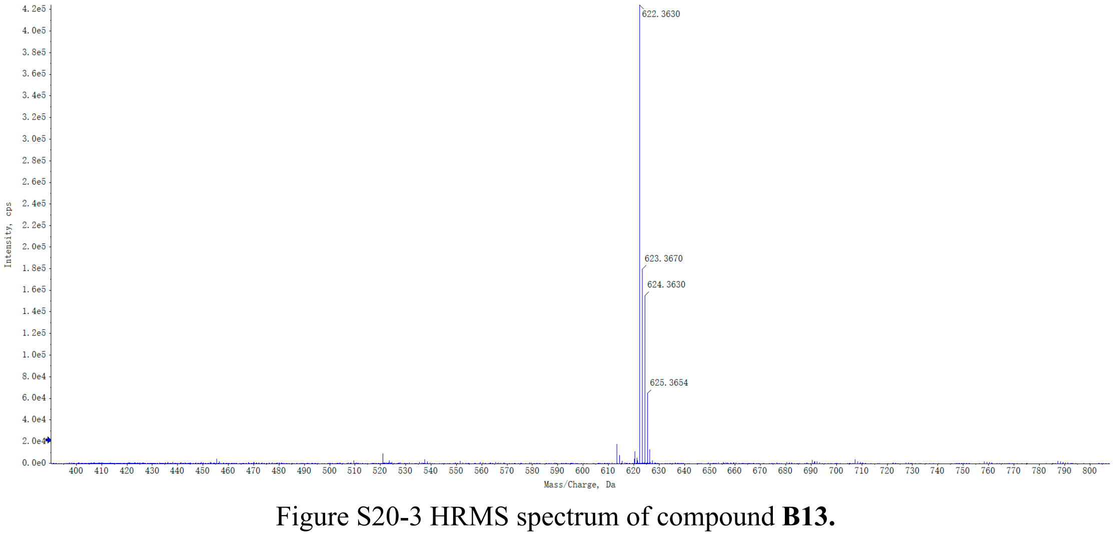

Figure S20-3 HRMS spectrum of compound **B13.**

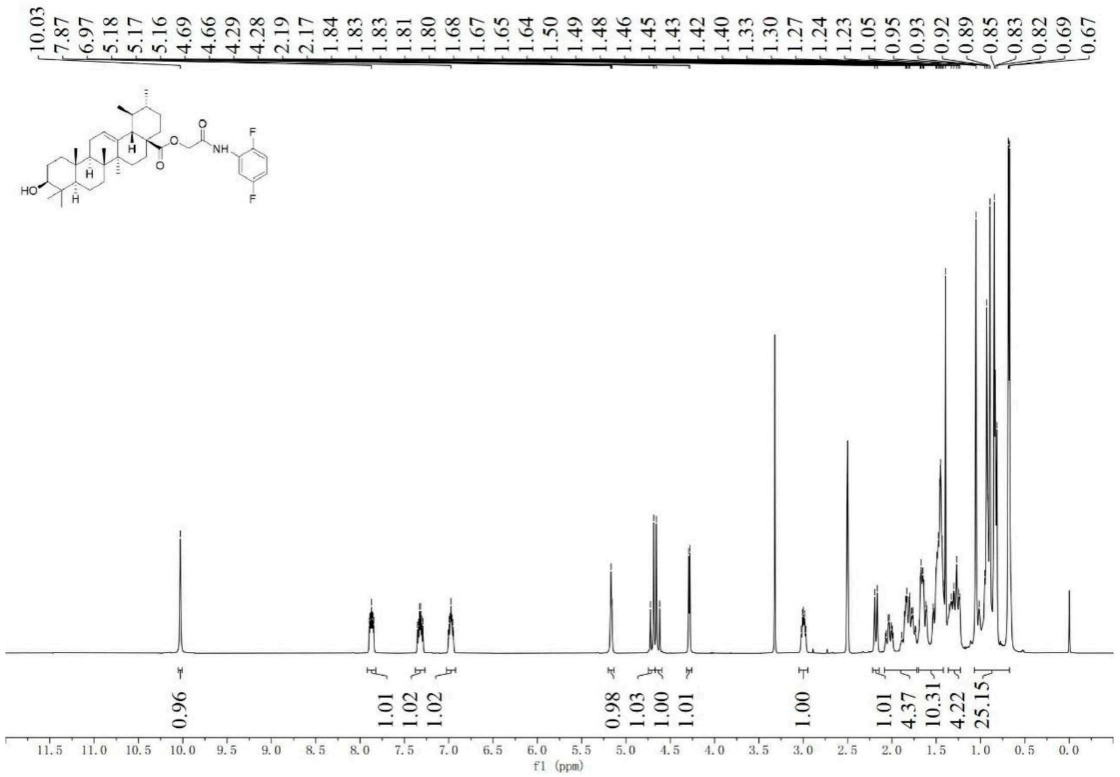

Figure S21-1 ^{1}H NMR spectrum of compound **B14.**

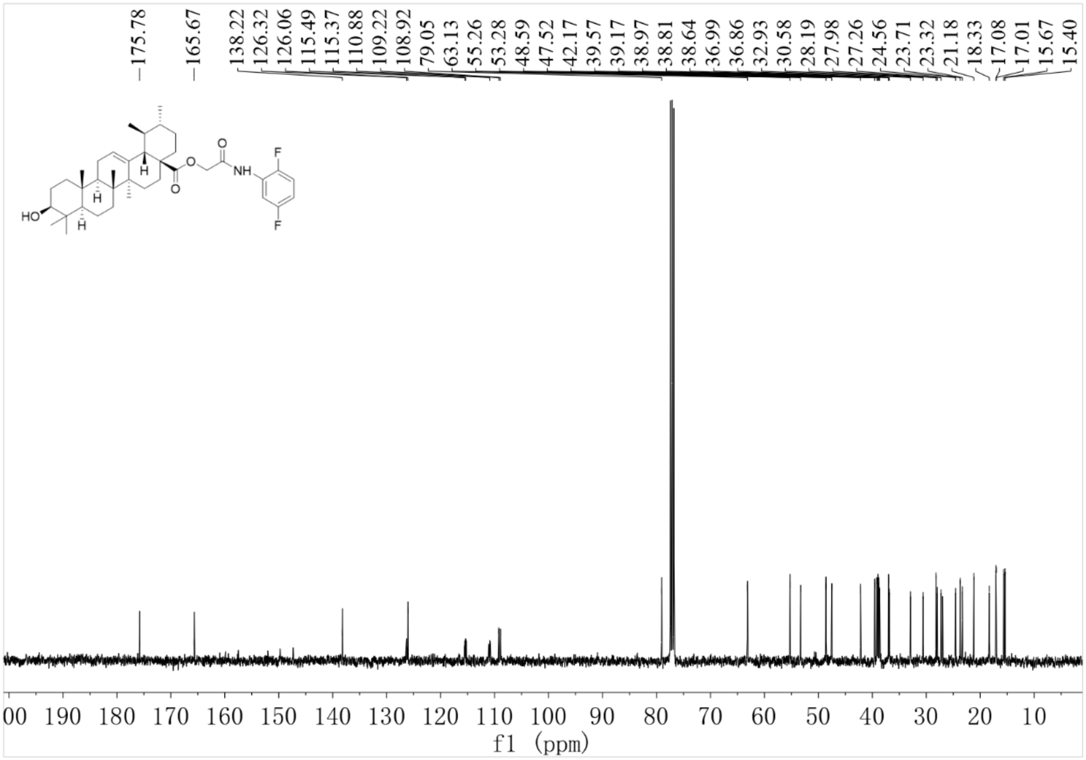

Figure S21-2 ^{13}C NMR spectrum of compound **B14.**

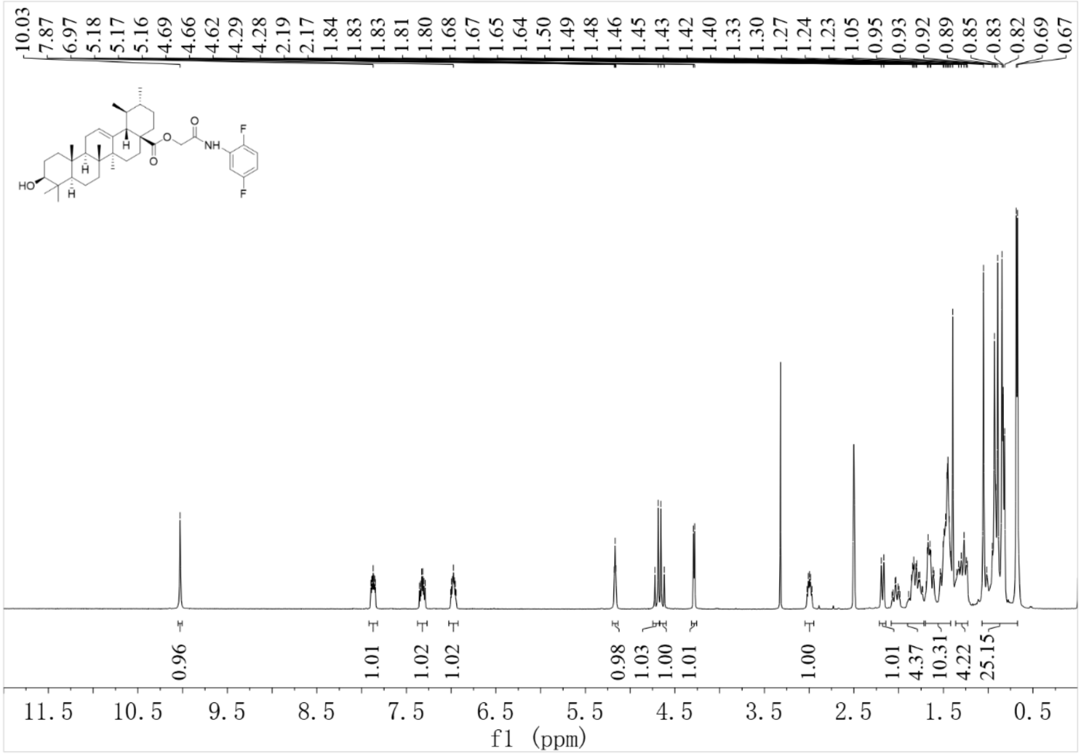

Figure S21-3 HRMS spectrum of compound **B14.**

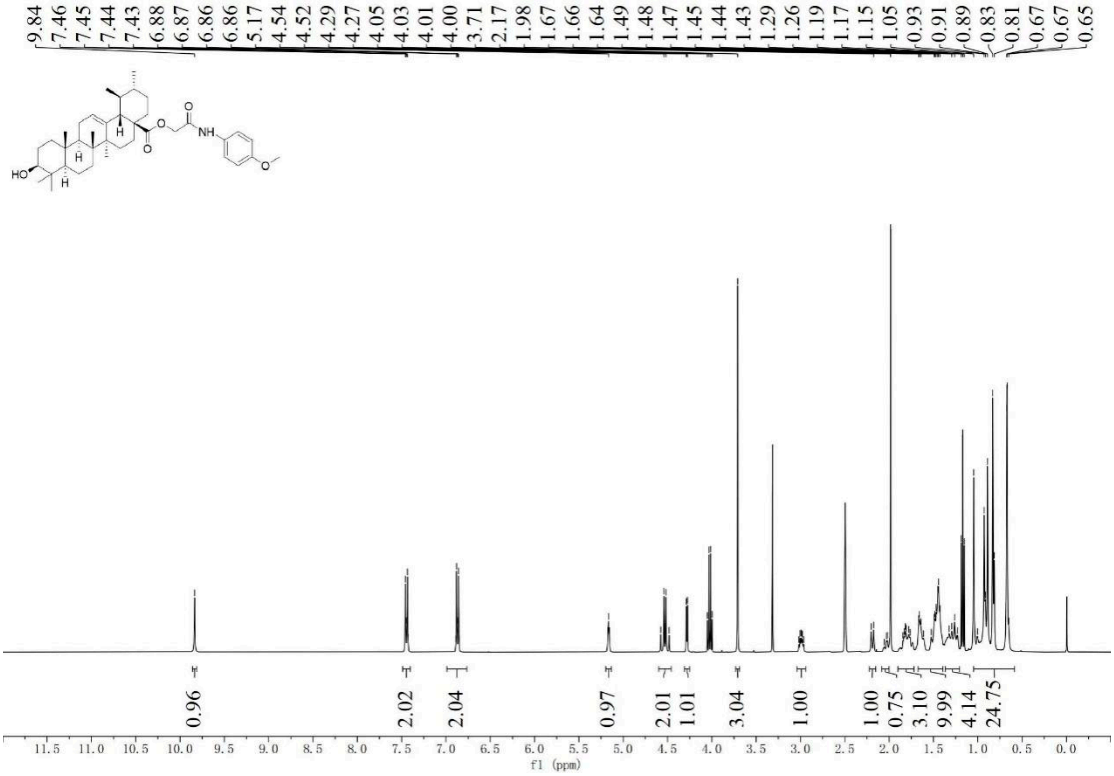

Figure S22-1 ^{1}H NMR spectrum of compound **B15.**

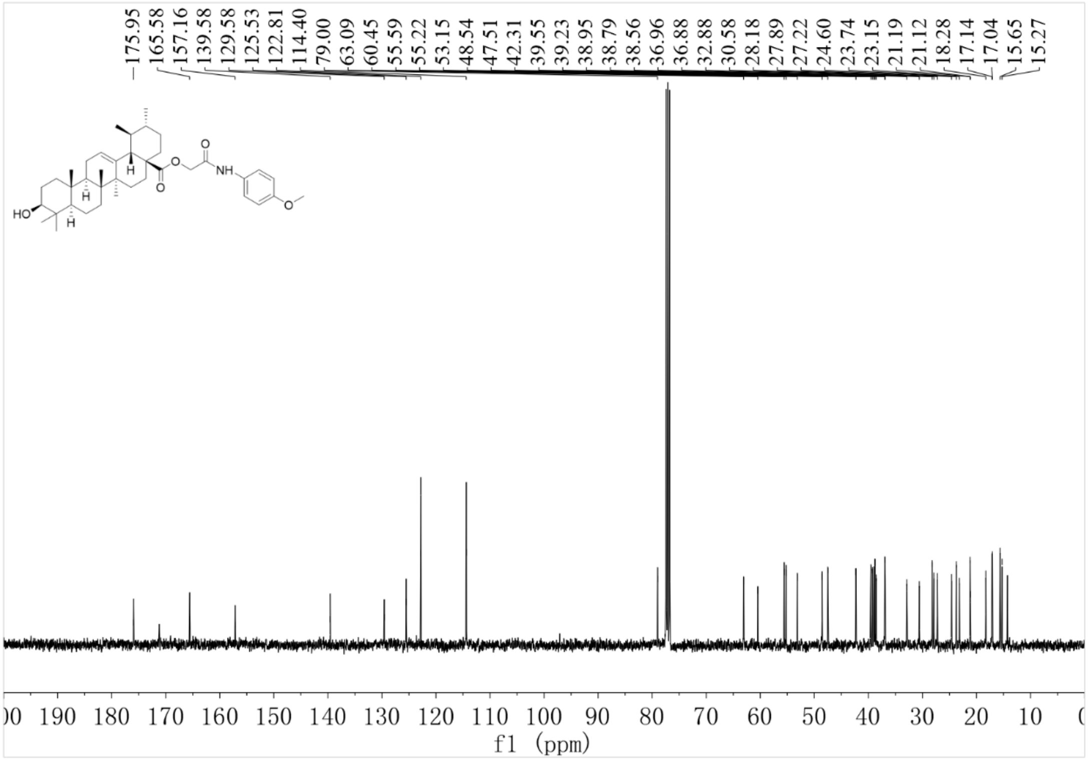

Figure S22-2 ^{13}C NMR spectrum of compound **B15.**

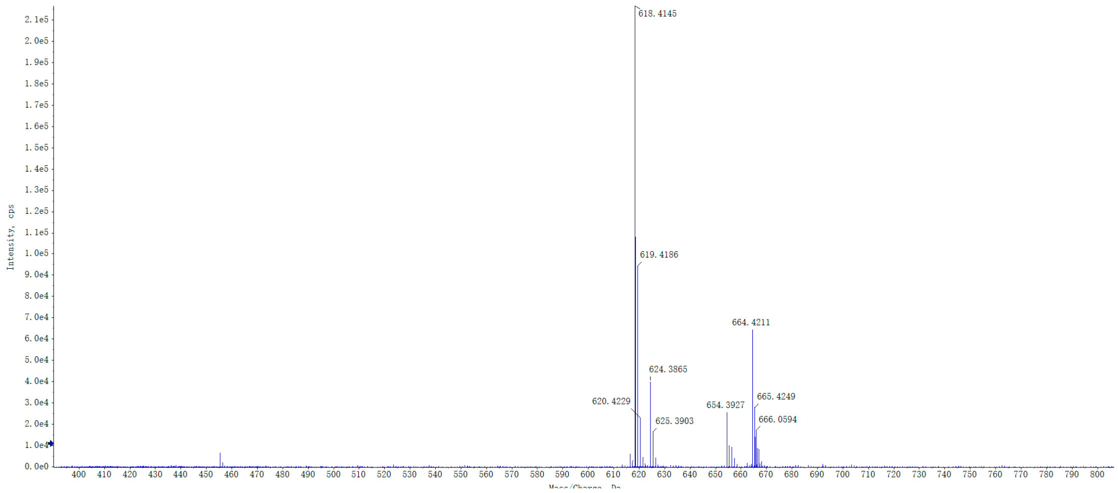

Figure S22-3 HRMS spectrum of compound **B15.**

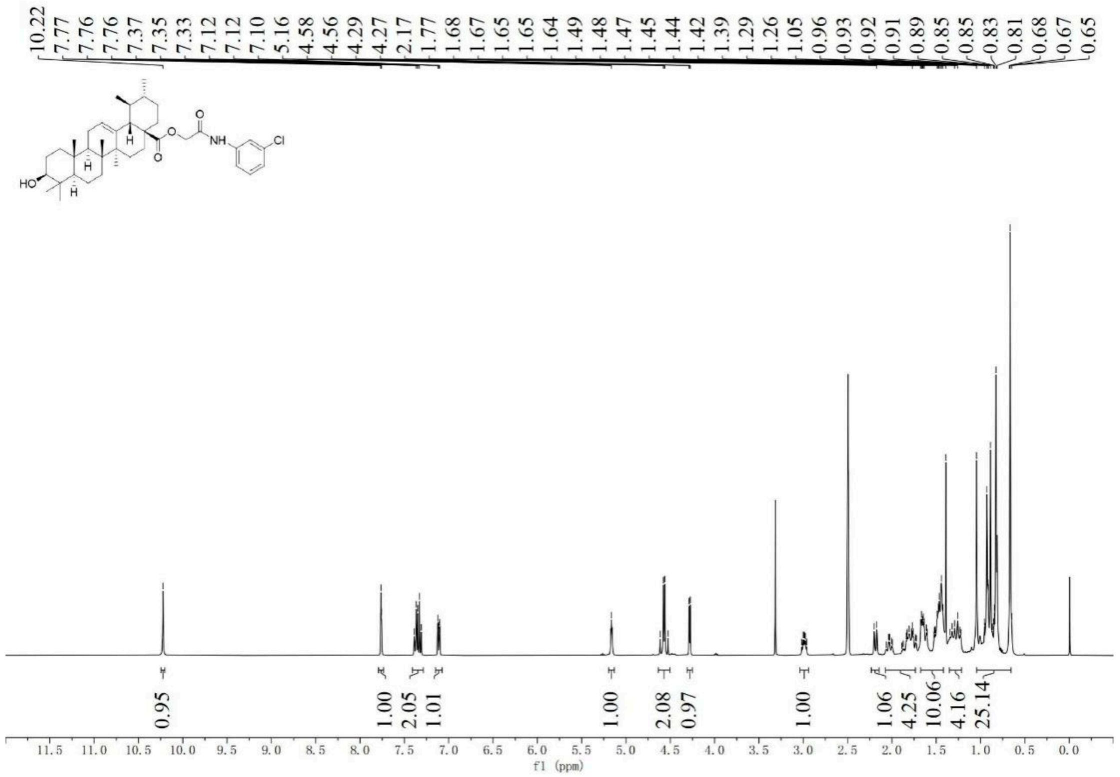

Figure S23-1 ^{1}H NMR spectrum of compound **B16.**

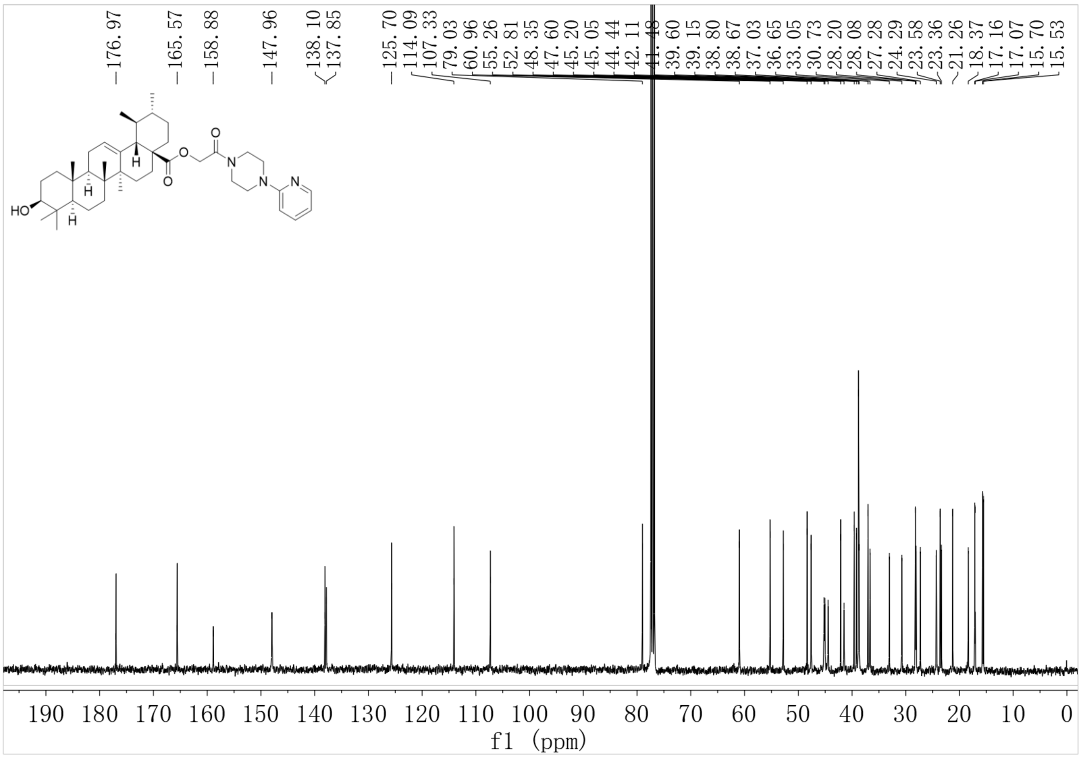

Figure S23-2 ^{13}C NMR spectrum of compound **B16.**

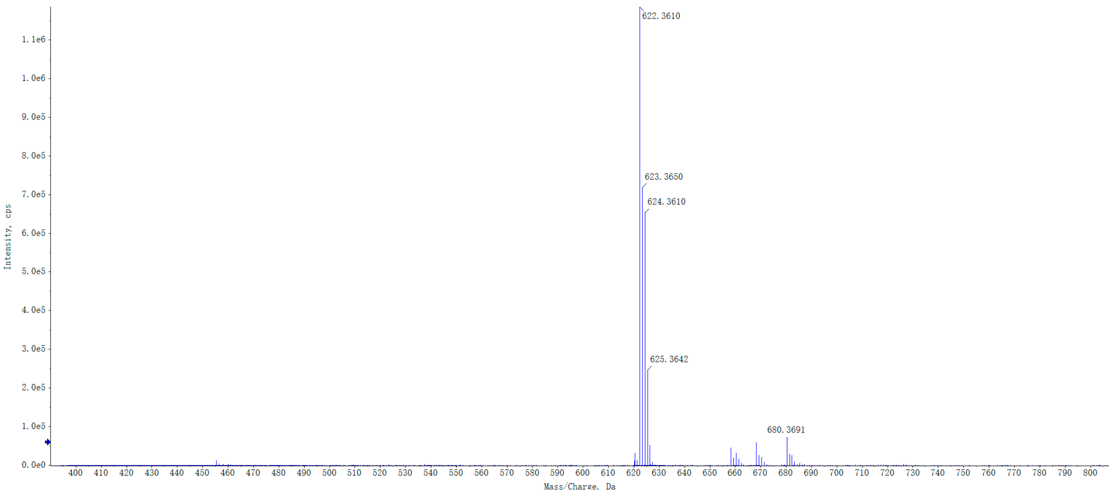

Figure S23-3 HRMS spectrum of compound **B16.**

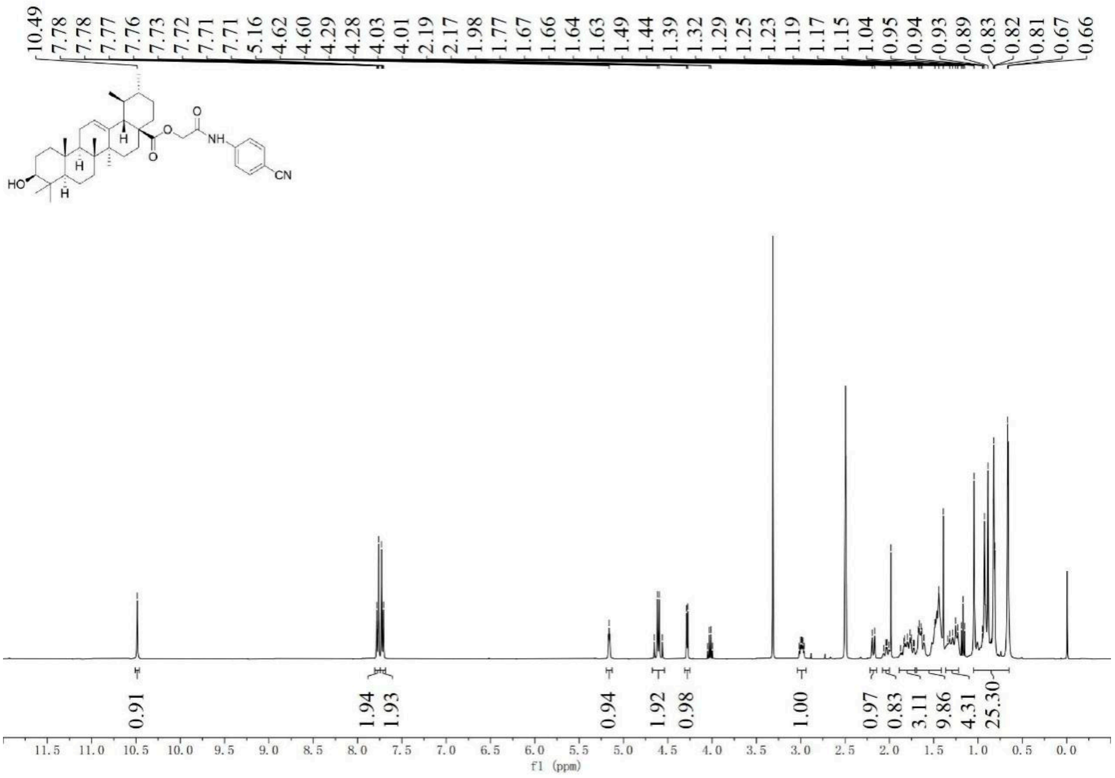

Figure S24-1 ^{1}H NMR spectrum of compound **B17.**

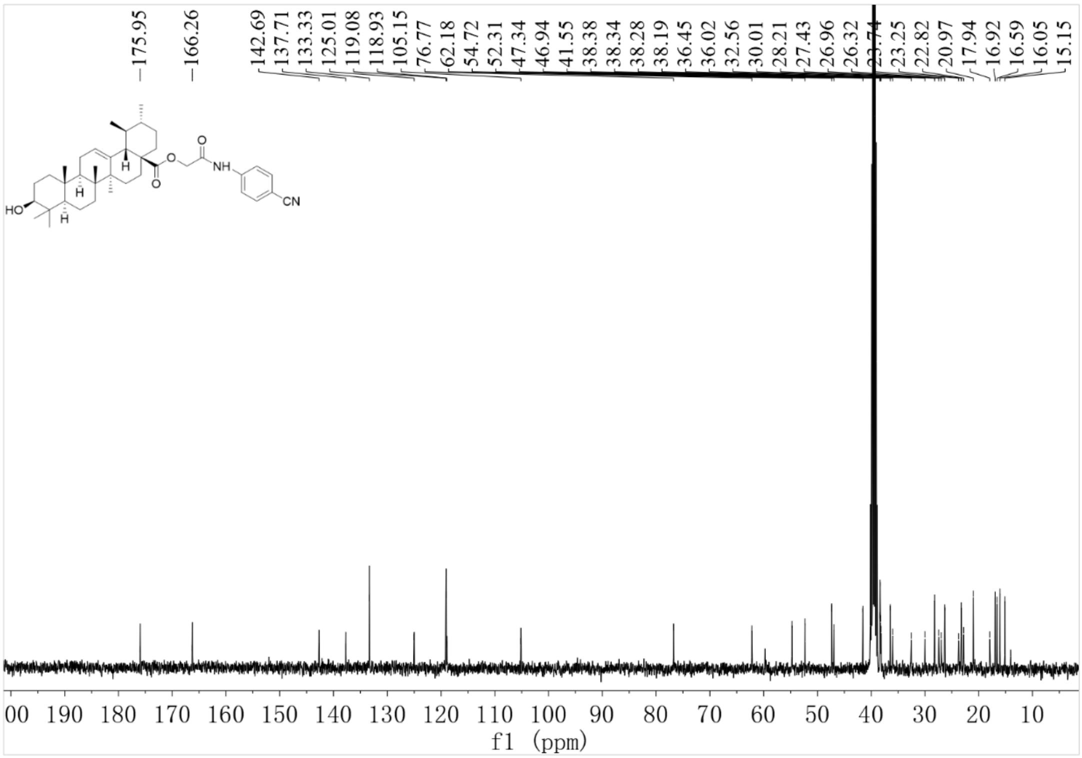

Figure S24-2 ^{13}C NMR spectrum of compound **B17.**

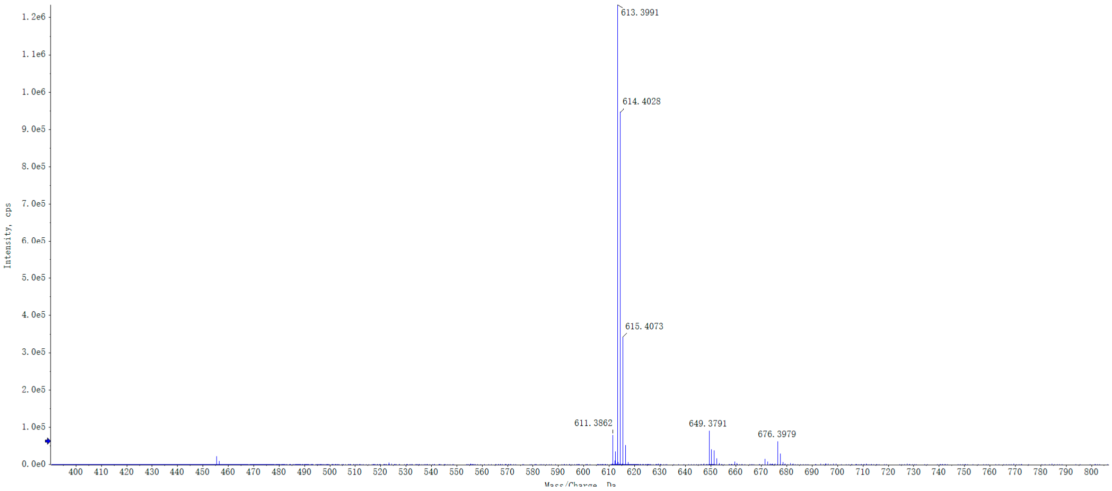

Figure S24-3 HRMS spectrum of compound **B17.**

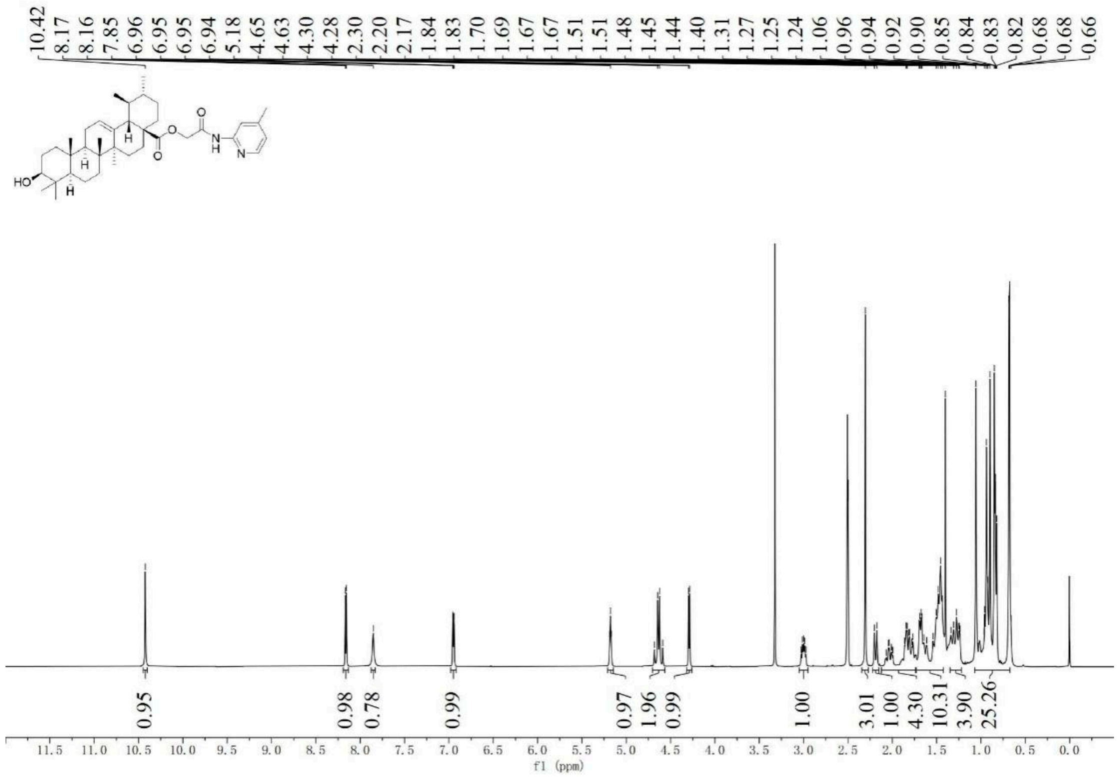

Figure S25-1 ^{1}H NMR spectrum of compound **B18.**

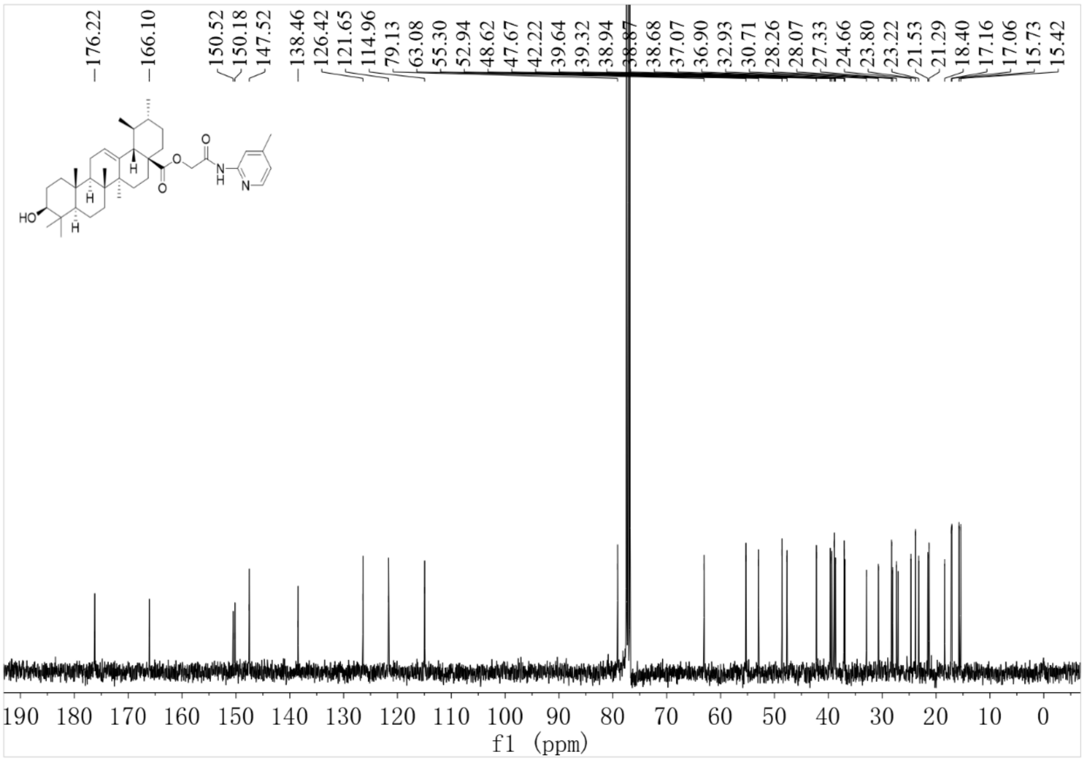

Figure S25-2 ^{13}C NMR spectrum of compound **B18.**

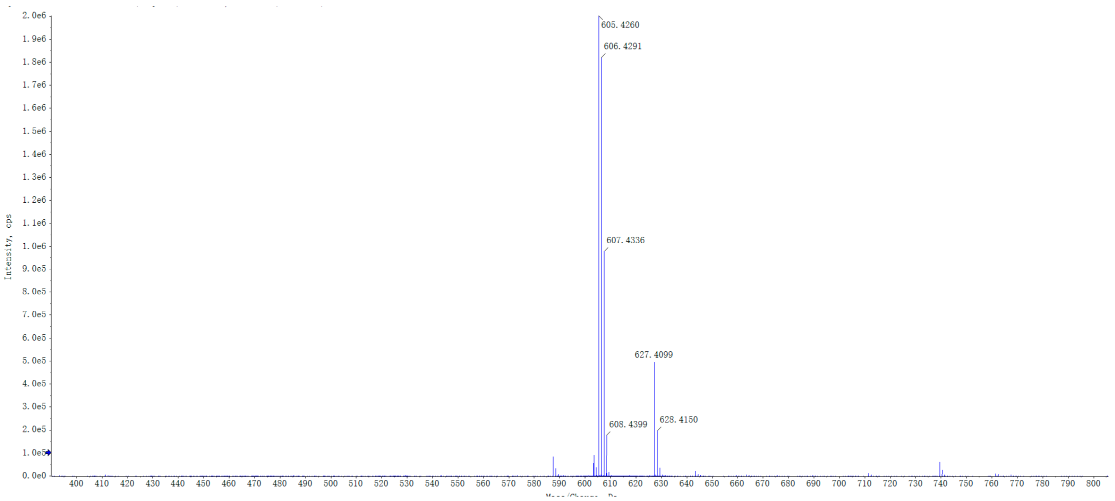

Figure S25-3 HRMS spectrum of compound **B18.**

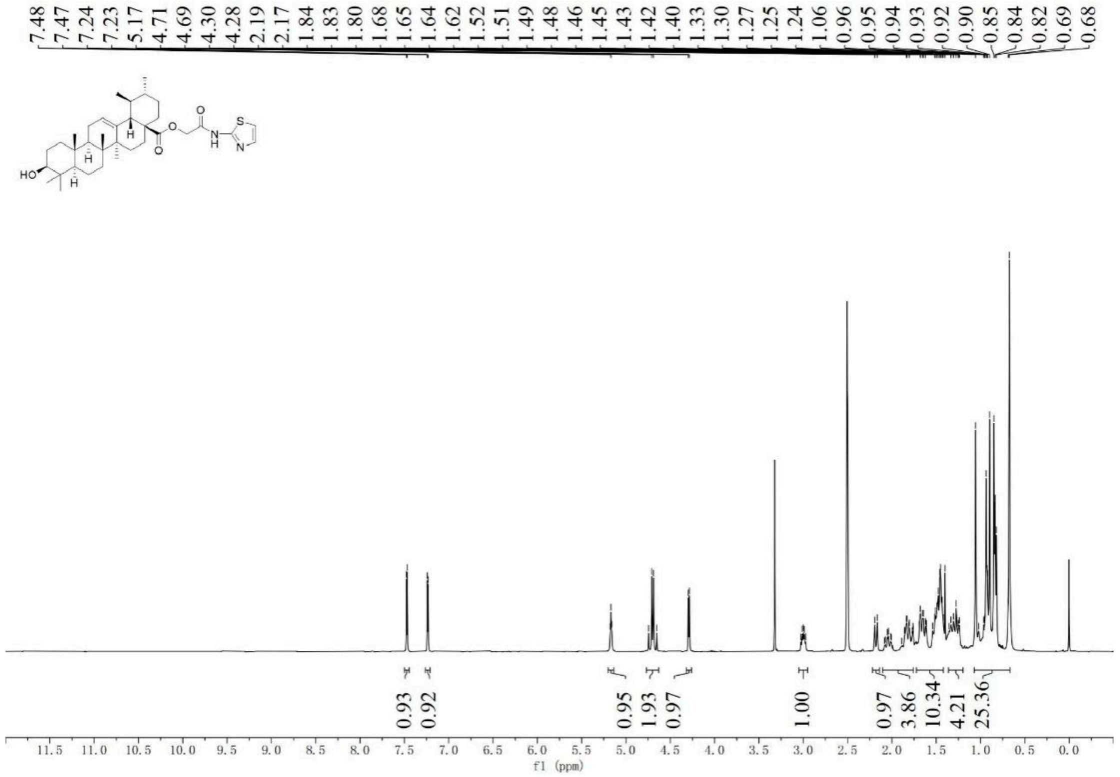

Figure S26-1 ^{1}H NMR spectrum of compound **B19.**

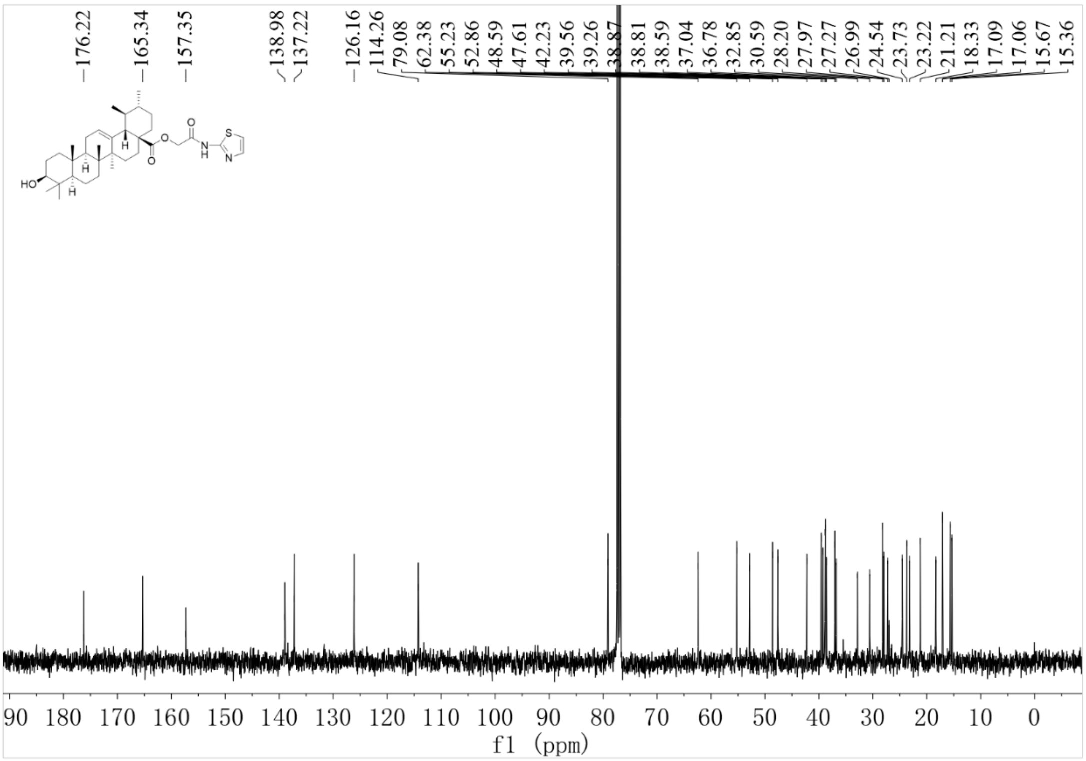

Figure S26-2 ^{13}C NMR spectrum of compound **B19.**

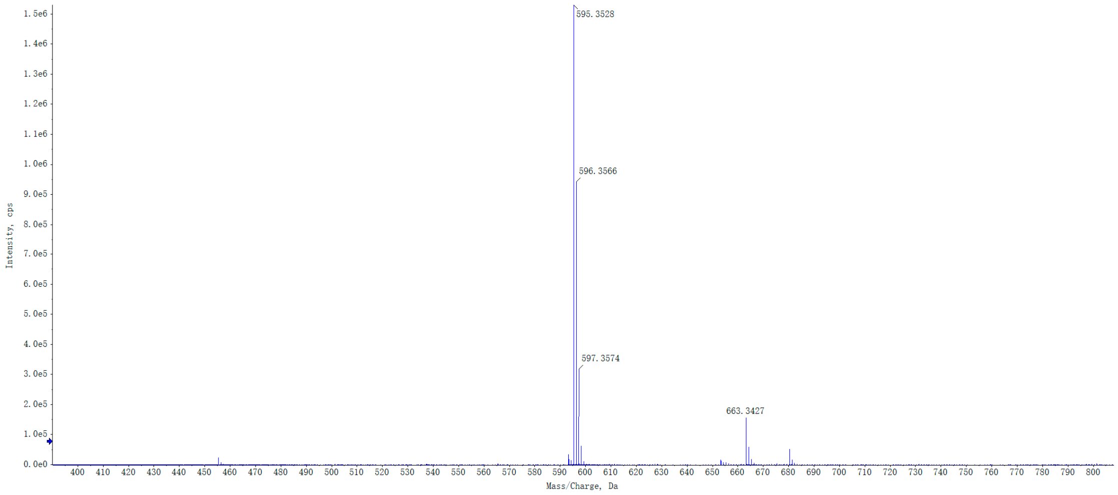

Figure S26-3 HRMS spectrum of compound **B19.**
